# Ethylenediamine Derived Carboxamides of Betulinic and Ursolic Acid as Potential Cytotoxic Agents

**DOI:** 10.3390/molecules23102558

**Published:** 2018-10-08

**Authors:** Michael Kahnt, Lucie Fischer (née Heller), Ahmed Al-Harrasi, René Csuk

**Affiliations:** 1 Division of Organic Chemistry, Martin-Luther-University Halle-Wittenberg, Kurt-Mothes-Str. 2, D-06120 Halle (Saale), Germany; michael.kahnt@chemie.uni-halle.de (M.K.); lucie.heller@chemie.uni-halle.de (L.F.); 2 Chair of Oman’s Medicinal Plants and Marine Natural Products, University of Nizwa, P.O. Box 33, Birkat Al-Mauz, Nizwa 611, Oman; aharrasi@unizwa.edu.om

**Keywords:** ursolic acid, betulinic acid, cytotoxicity, triterpenoids

## Abstract

Two easily accessible, natural occurring triterpenoids, betulinic and ursolic acid, were used as starting materials for the synthesis of novel cytotoxic agents. A set of 28 ethylenediamine-spacered carboxamides was prepared holding an additional substituent connected to the ethylenediamine group. The compounds were screened in SRB assays to evaluate their cytotoxic activity employing several human tumor cell lines. Betulinic acid-derived carboxamides **17**–**30** showed significantly higher cytotoxicity than their ursolic acid analogs **3**–**16**. In particular, compounds **25** and **26** were highly cytotoxic, as indicated by EC_50_ values lower than 1 μM.

## 1. Introduction

During the last decade, significant progress has been made in the therapy of cancer, with several major breakthroughs recorded in recent years. Two examples illustrate today’s medical advances in fighting cancer: In August 2017, the first adoptive cell immunotherapy (chimeric antigen receptor T-cell therapy) and the first gene therapy (tisagenlecleucel) were officially approved by the FDA [1]. However, the battle against cancer is far from being won, as the prognosis for many types of cancer remains poor. Despite the promising therapeutic benefits of immuno- or gene therapy, chemotherapy nonetheless remains a key means of cancer treatment. Therefore, research is and should still be focused on the development of new bioactive drugs applicable to chemotherapy. 

Many natural products show a wide range of pharmacological properties, including antiviral, antimalarial, anti-inflammatory and antitumor activities. Thus, they are considered as ideal lead structures for the development of new bioactive substances. Triterpenes are a class of pharmacologically interesting natural products that also exhibit cytotoxic properties among several other biological activities [2]. Our own work in recent years has been focused on modifications of naturally occurring triterpenes, such as ursolic, oleanolic, glycyrrhetinic, betulinic, boswellic, platanic and maslinic acid, in which cytotoxic agents were synthesized for their cancer-fighting properties. 

**Figure 1:**
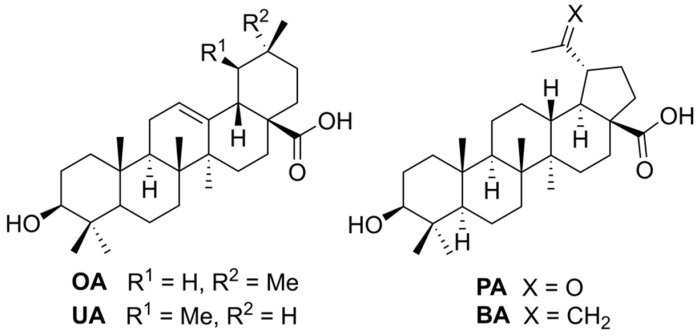
Structures of oleanolic (**OA**), ursolic (**UA**), platanic (**PA**) and betulinic acid (**BA**).

The objective of this work was to improve the cytotoxic properties of the two triterpenoids, ursolic (**UA**) and betulinic acid (**BA**), by structural modification at the C-28 carbonyl moiety. Many modifications of UA and BA have been reported [3,4,5], and the cytotoxicity of these analogs was determined. As a result, two major features seem important for obtaining compounds of high cytotoxicity: First, an intact carbonyl group at C-28 has to be present; reduction or removal of this moiety led to compounds of low cytotoxicity. Second, previous findings [6,7,8,9,10,11] reported an increased cytotoxicity for amino-substituted triterpenes, regardless of the position of this moiety. Hence, we introduced an amino function into the skeleton of the triterpene, and carboxamides with α,ω-diamines, such as ethylenediamine, were prepared. Furthermore, ethylenediamine-spacered carboxamides of oleanolic (**OA**) or platanic acid (**PA**) have previously been shown to be highly cytotoxic for human tumor cell lines [10,12]. Ursolic and betulinic acid were chosen as starting compounds: they are easily accessible and structurally related to **OA** and **PA**, respectively (Figure 1).

## 2. Results and Discussion

### 2.1. Chemistry

**Scheme 1:**
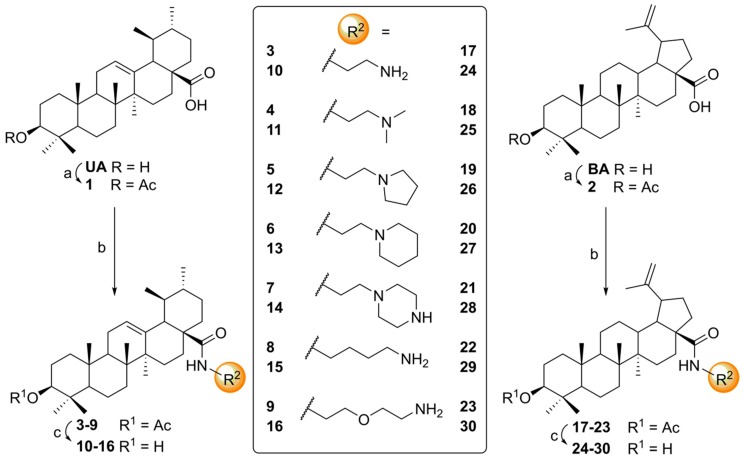
Synthesis of ursolic and betulinic carboxamides **3**–**30**: (**a**) Ac_2_O, NEt_3_, DCM, 25 °C, 2 days, yielding **1** (96%) and **2** (93%); (**b**) oxalyl chloride, DCM, DMF, 0–25 °C, 1 h, then amine, 25 °C, 2 h, yielding **3**–**9** and **17**–**23**; (**c**) MeOH/KOH, 25 °C, 2–3 days, yielding **10**–**16** and **24**–**30**.

Acetylation of betulinic (**BA**) and ursolic acid (**UA**) yielded acetates **1** and **2**. The carboxamides **3**–**30** were prepared in two steps (Scheme 1). Thus, the acetates **1** and **2** were treated with oxalyl chloride in dry dichloromethane in the presence of a catalytic amount of *N*,*N*-dimethylformamide to yield the corresponding acyl chlorides, which were used without further purification. Subsequent treatment of the crude acyl chlorides with various amines in dry dichloromethane provided carboxamides **3**–**9** and **17**–**23**. Their deacetylation yielded compounds **10**–**16** and **24**–**30**, respectively.

### 2.2. Biology

The cytotoxicity of ursolic and betulinic carboxamides **3**–**30** was determined in sulforhodamine B (SRB) assays [13]. The results of this screening are compiled in Table 1. 

**Figure 2:**
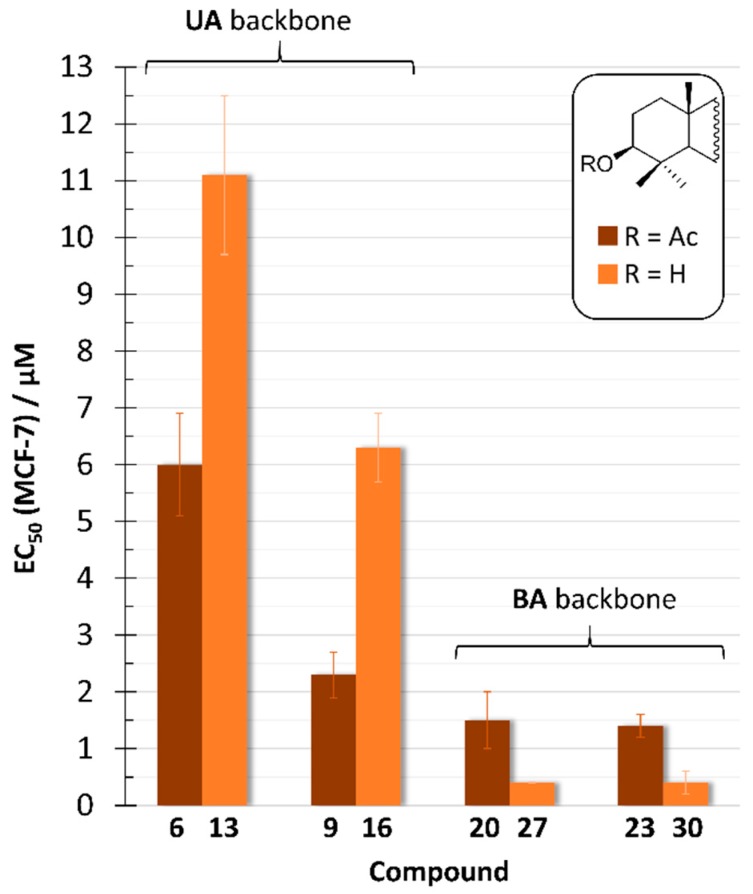
Comparison of EC_50_ values of selected ursolic and betulinic carboxamides for MCF-7 tumor cells.

Ursolic acid-derived amides **3**–**16** were found cytotoxic; their EC_50_ values were between 15 µM and 1 µM. For HT29 tumor cells and compound **9**, the most potent ursolic acid derivative, an EC_50_ value of 1.6 µM ± 0.10 µM was observed. Variation of the alkyl substituents at the terminal amino group of the ethylenediamine spacer (as in compounds **3**–**6** or **10**–**13**) did not influence the cytotoxic properties at all. Exceptions, however, are the compounds **6** and **13**: their EC_50_ values are significantly higher as compared to other ursolic carboxamides. For ethylenediamine-spacered carboxamides, substitution at position 3 with an acetyl group did not affect EC_50_ values. Close inspection of the results, however, revealed a small influence of the acetyl moiety at position C-3. Thus, 3-*O*-acetyl-derivatives **6**–**9** show significantly lower EC_50_ values than their deacetylated analogues **13**–**16** (Figure 2). 

Betulinic acid-derived carboxamides **17**–**30** were more cytotoxic than their corresponding ursolic acid derived analogs (**3**–**16**), showing EC_50_ values ranging between 5 µM and 0.2 µM. This is particularly evident for compounds **13** and **27** or **16** and **30** (Figure 2). The most active compounds, however, **25** and **26,** showed EC_50_ values of 0.2 µM ± 0.01 µM (**25**) and 0.3 µM ± 0.1 µM (**26**) for A2780 tumor cells. Compared to the known chemotherapeutic agent doxorubicin hydrochloride (**DRC**, EC_50_ = 0.01 ± 0.01 µM), the activities of **25** and **26** are reduced by factors of 20 and 30, respectively. With respect to the MCF-7 human tumor cell line, however, **25** (EC_50_ = 0.3 µM ± 0.07 µM) and **26** (EC_50_ = 0.2 µM ± 0.05 µM) are even more cytotoxic than the reference compound (**DRC**, EC_50_ = 1.1 ± 0.3 µM). Calculations using the SwissTargetPrediction suggest that **25** and **26** may be possible inhibitors of tyrosine phosphates [14].

Moreover, substituents exerted similar influence for betulinic acid as well as for ursolic acid-derived carboxamides. The presence of extra substituents at the ethylenediamine spacer also had no influence onto the cytotoxicity of the compounds. However, differences between 3-*O*-acetyl-derivatives **17**–**23** and deacetylated analogs **24**–**30** were observed. Interestingly, a different trend becomes visible for most of the compounds as compared to the carboxamides from ursolic acid. Thereby, compounds holding an acetyl moiety at position 3 are significantly less cytotoxic than the corresponding 3-hydroxy-derivatives. This trend is illustrated in Figure 2 for compounds **20** and **27** and **23** and **30**, respectively.

In addition to EC_50_ values, the selectivity (which is defined as selectivity index (SI); SI = EC_50_ (NIH 3T3)/EC_50_ (tumor cell line)) is an important parameter to characterize a compound’s cytotoxic activity. Therefore, the SI of each substance with respect to the individual tumor cell lines was determined (Table 2). Unfortunately, for most of the compounds, selectivity towards cancerous cells is quite poor, as indicated by SI factors close to 1. Some compounds even showed reverse selectivity behavior (SI < 1). Selectivity trends are best seen by close inspection of the SI factors for HT29 tumor cells. For ursolic acid derivatives **3**–**16**, selectivity towards HT29 cells is increased by removing the 3-*O*-acetyl moiety. The most selective ursolic acid derivative is compound **15,** with an SI of 2.67 for HT29 cells. Its acetylated analogue **8** only shows an SI of 0.76 for the same cancerous cell line. Similar to the trends observed for EC_50_ values, a different trend with regard to selectivity becomes visible for betulinic carboxamides **17**–**30** as compared to ursolic analogs. Removal of the 3-*O*-acetyl moiety seems to result in a loss of selectivity. The highest selectivity was observed for compound **18** with SI = 3.33 (NIH 3T3/HT29). Hence it is three times more cytotoxic towards HT29 tumor cells than towards nonmalignant mouse fibroblasts. Thus, its selectivity is only slightly smaller than that of the known chemotherapeutic agent doxorubicin hydrochloride (**DRC**), which shows an SI of 6 towards A2780 tumor cells. Interestingly, compound **25**, the deacetylated analog of **18**, shows no selectivity for HT29 tumor cells at all (SI = 1.00). For the two most active compounds **25** and **26**, decreased selectivity was observed. SI factors are close to 1 for most of the cancerous cell lines. However, both compounds are slightly selective towards A2780 tumor cells (**25**: SI = 1.5; **26**: SI = 1.33) and in case of compound **26**, selectivity for MCF-7 tumor cells was also observed (SI = 2.00). 

**Figure 3:**
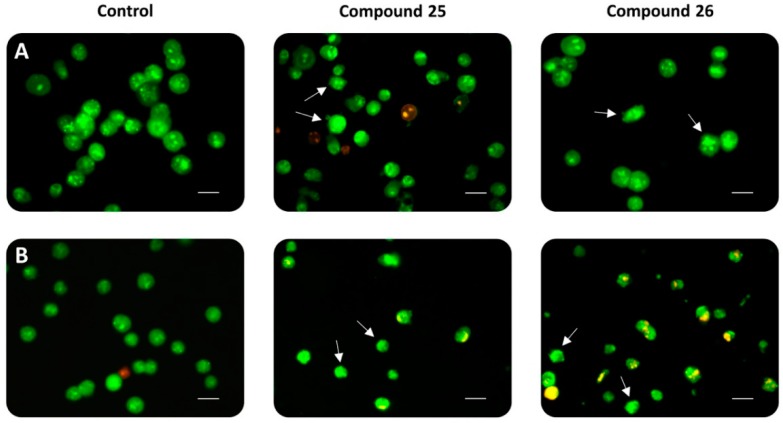
Fluorescence microscopic images: (**A**) treatment of MCF-7 cells with **25** (1 µM) and **26** (0.75 µM) for 24 h; (**B**) treatment of A2780 cells with **25** (1 µM) and **26** (1 µM) for 24 h; scale bar = 10 µm, AO and PI were used.

Extended biological investigations were performed for compounds **25** and **26** using dye exclusion acridine orange (AO)/propidium iodide (PI) assays by treating MCF-7 or A2780 tumor cells with **25** and **26**, respectively for 24 h (Figure 3). Fluorescence microscopic images of MCF-7 cells treated with compound **25** showed, in addition to many vital cells, both apoptotic (membrane blebbing, marked by white arrows in Figure 3) and necrotic cells (red staining). After treatment of MCF-7 cells with **26,** intact cell membranes (green staining) were observed. Close inspection revealed the presence of protrusions in the plasma membrane (blebbing) of some cells, which can be considered a feature of apoptosis. A2780 human tumor cells treated with **25** (1 µM) or **26** (1 µM) showed both green colored cells as well as late-stage apoptotic cells recognizable by orange stained nuclei. For both compounds, membrane blebbing of some cells could also be observed. These observations can be regarded as indications of apoptosis. This parallels previous findings for related oleanolic [12] and platanic carboxamides [10], whose cytotoxicity mechanisms were investigated using Annexin V-FITC/PI assays and cell cycle evaluation, and which showed them to trigger cell death by apoptosis.

## 3. Materials and Methods

### 3.1. General

NMR spectra were recorded using the Varian spectrometers Gemini 2000 or Unity 500 (δ given in ppm, *J* in Hz; typical experiments: APT, H-H-COSY, HMBC, HSQC, NOESY), MS spectra were recorded on a Finnigan MAT LCQ 7000 (electrospray, voltage 4.1 kV, sheath gas nitrogen) instrument. The optical rotations were measured on a Perkin-Elmer polarimeter at 20 °C; TLC was performed on silica gel (Merck 5554, detection with cerium molybdate reagent); melting points are uncorrected (Leica hot stage microscope), and elemental analyses were performed on a Foss-Heraeus Vario EL (CHNS) unit. IR spectra were recorded on a Perkin Elmer FT-IR spectrometer Spectrum 1000. The solvents were dried according to usual procedures. The purity of the compounds was determined by HPLC and found to be >96%. Ursolic (**UA**) and betulinic acids (**BA**) were obtained from betulinines (Stříbrná Skalice, Czech Republic) in bulk quantities. Fluorescence microscopic images were recorded on an Axioskop 20 with an AxioCam MR3 (Carl Zeiss AG, Oberkochen, Germany). 

### 3.2. Biology

#### 3.2.1. Cell Lines and Culture Conditions

The cell lines used were human cancer cell lines: 518A2 (melanoma), A2780 (ovarian carcinoma), HT29 (colon adenocarcinoma), MCF-7 (breast adenocarcinoma), 8505C (thyroid carcinoma) and non-malignant mouse fibroblasts NIH 3T3. Cultures were maintained as monolayers in RPMI 1640 medium with l-glutamine (Capricorn Scientific GmbH, Ebsdorfergrund, Germany) supplemented with 10% heat inactivated fetal bovineserum (Sigma-Aldrich Chemie GmbH, Steinheim, Germany) and penicillin/streptomycin (Capricorn Scientific GmbH, Ebsdorfergrund, Germany) at 37 °C in a humidified atmosphere with 5% CO_2_.

#### 3.2.2. Cytotoxic Assay (SRB)

The cytotoxicity of the compounds was evaluated using the sulforhodamine-B (Kiton-Red S, ABCR) micro culture colorimetric assay. Cells were seeded into 96-well plates on day 0 at appropriate cell densities to prevent confluence of the cells during the period of experiment. After 24 h, the cells were treated with 6 different concentrations (1, 3, 7, 12, 20 and 30 µM) minimum. The final concentration of DMSO/DMF never exceeded 0.5%, which was non-toxic to the cells. After a 96 h treatment, the supernatant medium from the 96-well plates was discarded, the cells were fixed with 10% trichloroacetic acid (TCA) and allowed to rest at 4 °C. After 24 h fixation, the cells were washed in a strip washer and dyed with SRB solution (100 µL, 0.4% in 1% acetic acid) for about 20 min. After dying, the plates were washed four times with 1% acetic acid to remove the excess of the dye and allowed to air-dry overnight. Tris base solution (200 µL, 10 mM) was added to each well and absorbance was measured at λ = 570 nm using a 96-well plate reader (Tecan Spectra, Crailsheim, Germany). The EC_50_ values were averaged from three independent experiments performed each in triplicate calculated from semi logarithmic dose response curves applying a non-linear 4P Hills-slope equation (GraphPad Prism5; variables top and bottom were set to 100 and 0, respectively).

#### 3.2.3. AO/PI Dye Exclusion Test

Morphological characteristics of cell death were analyzed employing an AO/PI assay using human cancer cell lines A2780 and MCF-7. Approximately 8 × 10^5^ cells were seeded in cell culture flasks (25 cm^2^), and the cells were allowed to grow for 24 h. After removing the used medium, fresh medium was reloaded (or a blank new medium was used as a control). After 24 h, the content of the flask was collected and centrifuged (1200 rpm, 4 °C), and the pellet was gently suspended in phosphate-buffered saline (PBS (*w*/*w*), 1 mL) and centrifuged again. The PBS was removed, and the pellet gently suspended in PBS (50 µL) again. The analysis of the cells was performed using a fluorescence microscope after having mixed the cell suspension (10 µL) with a solution of AO/PI (1 µg/mL, 10 µL). 

## 4. Experimental

### 4.1. General Procedure A for the Acetylation of Triterpenoic Acids (***1***–***2***)

To a solution of **UA** or **BA** (5 g, 11 mmol) in dry DCM (150 mL) was added triethylamine (4.6 mL, 33 mmol), acetic anhydride (3.1 mL, 33 mmol) and DMAP (cat.). After stirring for 2 days at 25 °C, a saturated solution of NH_3_ in MeOH was added (3 mL), and the mixture was stirred for another 30 min. Dilution with DCM and subsequent aqueous work-up provided crude 3-*O*-acetyl-UA or 3-*O*-acetyl-BA. Recrystallization from EtOH yielded pure acetates **1** (5.3 g, 96%) and **2** (5.1 g, 93%) both as colorless solids, whose analytical and spectroscopic data were in full agreement with data from the literature. 

### 4.2. General Procedure B for the Synthesis of Triterpenoic Amides (***3***–***9***, ***17***–***23***)

Compounds **1** or **2** (0.8 mmol) were each dissolved in dry DCM (15 mL), cooled to 0 °C, and oxalyl chloride (3.2 mmol) and dry DMF (3 drops) were added. After warming to 25 °C, the mixture was stirred for 1 h. The solvent was removed under reduced pressure, re-evaporated with dry THF (4 × 15 mL), and the residue was immediately resolved in dry DCM (10 mL). This mixture was then added dropwise to a solution of the amine (2.4 mmol) in dry DCM (5 mL) and stirred at 25 °C for 2 h. After usual aqueous work-up, the solvent was removed under reduced pressure, and the crude product was subjected to column chromatography (silica gel, chloroform/methanol mixtures). Compounds **3**–**9** and **17**–**23** were each obtained as colorless solids. 

### 4.3. General Procedure C for Deacetylation of Triterpenoic Amides (***10***–***16***, ***24***–***30***)

To a solution of acetylated amides **3**–**9** or **17**–**23** (0.33 mmol) in methanol (10 mL) was added a solution of potassium hydroxide (1.65 mmol) in methanol (2 mL). The mixture was stirred at 25 °C for 2 or 3 days. After completion of the reaction (as indicated by TLC), aq. HCl was added until pH = 7. After usual work-up, the solvent was removed under reduced pressure, and the residue was subjected to column chromatography (silica gel, chloroform/methanol mixtures) yielding compounds **10**–**16** and **24**–**30** each as colorless solids. 

*(3β)-3-Acetyloxy-urs-12-en-28-oic acid* (**1**). Compound **1** was prepared according to general procedure A from ursolic acid. Yield: 96%; m.p. 287–290 °C (lit.: 289–290 °C [15]).

*(3β)-3-Acetyloxy-lup-20(29)en-28-oic acid* (**2**). Compound **2** was prepared according to general procedure A from betulinic acid. Yield: 93%; m.p. 281–284 °C (lit.: 280-282 °C [16]).

*(3β)-N-(2-Aminoethyl)-3-acetyloxy-urs-12-en-28-amide* (**3**). Compound **3** was prepared from **1** according to general procedure B using ethylenediamine. Column chromatography (SiO_2_, CHCl_3_/MeOH 9:1) gave **3** (yield: 80%); m.p. 202–205 °C (lit.: 140–142 °C [17]); [δ]_D_ = +39.4° (*c* 0.355, CHCl_3_); R_f_ = 0.48 (CHCl_3_/MeOH 9:1); IR (KBr): ν = 3413*br s*, 2948*s*, 1735*s*, 1633*s*, 1526*s*, 1456*s*, 1370*s*, 1247*s*, 1174*w*, 1147*w*, 1092*w*, 1028*s*, 1006*m*, 986*m*, 755*m* cm^−1^; ^1^H-NMR (500 MHz, CDCl_3_): *δ* = 6.88 (*t*, *J* = 5.3 Hz, 1H, NH), 5.34 (*t*, *J* = 3.3 Hz, 1H, 12-H), 4.49 (*dd*, *J* = 10.0, 5.9 Hz, 1H, 3-H), 3.62–3.54 (*m*, 1H, 31-H_a_), 3.38–3.30 (*m*, 1H, 31-H_b_), 3.13–3.01 (*m*, 2H, 32-H_a_, 32-H_b_), 2.09–2.04 (*m*, 1H, 18-H), 2.04 (*s*, 3H, Ac), 2.03–1.87 (*m*, 3H, 16-H_a_, 11-H_a_, 11-H_b_), 1.82–1.22 (*m*, 15H, 22-H_a_, 16-H_b_, 1-H_a_, 15-H_a_, 2-H_a_, 2-H_b_, 9-H, 22-H_b_, 6-H_a_, 21-H_a_, 7-H_a_, 19-H, 6-H_b_, 7-H_b_, 21-H_b_), 1.08 (*s*, 3H, 27-H), 1.07–0.95 (*m*, 3H, 1-H_b_, 15-H_b_, 20-H), 0.96–0.92 (*m*, 4H, 25-H, 20-H), 0.89–0.85 (*m*, 6H, 23-H, 29-H), 0.85 (*s*, 3H, 24-H), 0.84–0.80 (*m*, 1H, 5-H), 0.74 (*s*, 3H, 26-H) ppm; ^13^C-NMR (126 MHz, CDCl_3_): *δ* = 180.2 (C-28), 171.1 (Ac), 139.3 (C-13), 126.0 (C-12), 81.0 (C-3), 55.4 (C-5), 53.1 (C-18), 47.9 (C-17), 47.6 (C-9), 42.4 (C-14), 40.6 (C-32), 39.8 (C-19), 39.7 (C-8), 39.0 (C-20), 38.7 (C-31), 38.5 (C-1), 37.8 (C-4), 37.4 (C-22), 37.0 (C-10), 32.8 (C-7), 31.0 (C-21), 28.2 (C-23), 28.0 (C-15), 24.8 (C-16), 23.7 (C-2), 23.5 (C-11), 23.5 (C-27), 21.4 (Ac), 21.3 (C-30), 18.3 (C-6), 17.4 (C-29), 17.2 (C-26), 16.9 (C-24), 15.7 (C-25) ppm; MS (ESI, MeOH): *m*/*z* = 541 (100 %, [M + H]^+^); analysis calcd for C_34_H_56_N_2_O_3_ (540.83): C 75.51, H 10.44, N 5.18; found: C 75.32, H 10.61, N 5.01.

*(3β)-N-[2-(Dimethylamino)ethyl]-3-acetyloxy-urs-12-en-28-*amide (**4**). Compound **4** was prepared from **1** according to general procedure B using *N*,*N*-dimethylethylenediamine. Column chromatography (SiO_2_, CHCl_3_/MeOH 95:5) gave **4** (yield: 88%); m.p. 121–124 °C; [α]_D_ = +44.9° (*c* 0.300, CHCl_3_); R_f_ = 0.49 (CHCl_3_/MeOH 9:1); IR (KBr): ν = 3422*br m*, 2937*m*, 1734*m*, 1636*m*, 1522*w*, 1457*m*, 1384*s*, 1247*m*, 1028*m* cm^−1^; ^1^H-NMR (500 MHz, CDCl_3_): *δ* = 6.68 (*t*, *J* = 5.0 Hz, 1H, NH), 5.33 (*t*, *J* = 3.4 Hz, 1H, 12-H), 4.48 (*dd*, *J* = 10.5, 5.6 Hz, 1H, 3-H), 3.62–3.52 (*m*, 1H, 31-H_a_), 3.29–3.21 (*m*, 1H, 31-H_b_), 2.83 (*t*, *J* = 5.3 Hz, 2H, 32-H), 2.58 (*s*, 6H, 33-H, 33′-H), 2.03 (*s*, 3H, Ac), 2.02–1.86 (*m*, 4H, 16-H_a_, 18-H, 11-H_a_, 11-H_b_), 1.83–1.76 (*m*, 1H, 22-H_a_), 1.73–1.67 (*m*, 1H, 16-H_b_), 1.67–1.57 (*m*, 4H, 1-H_a_, 15-H_a_, 2-H_a_, 2-H_b_), 1.57–1.26 (*m*, 9H, 9-H, 6-H_a_, 7-H_a_, 21-H_a_, 22-H_b_, 19-H, 6-H_b_, 21-H_b_, 7-H_b_), 1.07 (*s*, 3H, 27-H), 1.06–0.94 (*m*, 3H, 1-H_b_, 15-H_b_, 20-H), 0.93 (*d*, *J* = 6.1 Hz, 3H, 30-H), 0.93 (*s*, 3H, 25-H), 0.87 (*d*, *J* = 6.5 Hz, 3H, 29-H), 0.85 (*s*, 3H, 23-H), 0.84 (*s*, 3H, 24-H), 0.83–0.80 (*m*, 1H, 5-H), 0.75 (*s*, 3H, 26-H) ppm; ^13^C-NMR (126 MHz, CDCl_3_): *δ* = 179.1 (C-28), 171.1 (Ac), 139.2 (C-13), 126.0 (C-12), 81.0 (C-3), 57.7 (C-32), 55.4 (C-5), 53.4 (C-18), 47.9 (C-17), 47.6 (C-9), 44.6 (C-33, C-33′), 42.4 (C-14), 39.8 (C-19), 39.7 (C-8), 39.0 (C-20), 38.4 (C-1), 37.8 (C-4), 37.4 (C-22), 37.0 (C-10), 35.9 (C-31), 32.8 (C-7), 31.0 (C-21), 28.2 (C-23), 28.0 (C-15), 24.8 (C-16), 23.7 (C-2), 23.5 (C-11), 23.4 (C-27), 21.4 (Ac), 21.3 (C-30), 18.3 (C-6), 17.3 (C-29), 17.1 (C-26), 16.9 (C-24), 15.7 (C-25) ppm; MS (ESI, MeOH): *m*/*z* = 569 (100%, [M + H]^+^); analysis calcd for C_36_H_60_N_2_O_3_ (568.89): C 76.01, H 10.63, N 4.92; found: C 75.87, H 10.84, N 4.69.

*(3β)-N-(2-Pyrrolidin-1-ylethyl)-3-acetyloxy-urs-12-en-28-amide* (**5**). Compound **5** was prepared from **1** according to general procedure B using 1-(2-aminoethyl)-pyrrolidine. Column chromatography (SiO_2_, CHCl_3_/MeOH 95:5) gave **5** (yield: 92%). m.p. 156–159 °C; [α]_D_ = +45.9° (*c* 0.355, CHCl_3_); R_f_ = 0.44 (CHCl_3_/MeOH 9:1); IR (KBr): ν = 3404*br s*, 2948*s*, 1734*s*, 1651*s*, 1526*s*, 1456*s*, 1371*s*, 1246*s*, 1146*w*, 1091*m*, 1027*s*, 1006*m*, 985*m*, 753*m* cm^−1^; ^1^H-NMR (500 MHz, CDCl_3_): δ = 6.90 (*dd*, *J* = 11.5, 5.6 Hz, 1H, NH), 5.36 (*dd*, *J* = 7.1, 3.6 Hz, 1H, 12-H), 4.47 (*dd*, *J* = 10.5, 5.3 Hz, 1H, 3-H), 3.89–3.82 (*m*, 1H, 31-H_a_), 3.82–3.73 (*m*, 2H, 33-H_a_, 33′-H_a_) 3.41–3.32 (*m*, 1H, 31-H_b_), 3.30–3.21 (*m*, 2H, 32-H), 2.93–2.83 (*m*, 2H, 33-H_b_, 33′-H_b_), 2.23–2.06 (*m*, 4H, 34-H, 34′-H), 2.02 (*s*, 3H, Ac), 2.01–1.95 (*m*, 2H, 16-H_a_, 18-H), 1.95–1.90 (*m*, 2H, 11-H_a_, 11-H_b_), 1.76–1.56 (*m*, 6H, 22-H_a_, 16-H_b_, 1-H_a_, 15-H_a_, 2-H_a_, 2-H_b_), 1.55–1.41 (*m*, 5H, 9-H, 6-H_a_, 22-H_b_, 7-H_a_, 21-H_a_), 1.41–1.21 (*m*, 4H, 19-H, 6-H_b_, 7-H_b_, 21-H_b_), 1.06 (*s*, 3H, 27-H), 1.10–0.94 (*m*, 3H, 1-H_b_, 15-H_b_, 20-H), 0.92 (*s*, 3H, 25-H), 0.91 (*d*, *J* = 6.1 Hz, 3H, 30-H), 0.86 (*d*, *J* = 6.5 Hz, 3H, 29-H), 0.84 (*s*, 3H, 23-H), 0.83 (*s*, 3H, 24-H), 0.82–0.78 (*m*, 1H, 5-H), 0.71 (*s*, 3H, 26-H) ppm; ^13^C-NMR (126 MHz, CDCl_3_): δ = 179.8 (C-28), 171.1 (Ac), 138.9 (C-13), 126.0 (C-12), 80.9 (C-3), 55.3 (C-5), 55.0 (C-32), 54.8 (C-33, C-33′), 52.9 (C-18), 47.9 (C-17), 47.5 (C-9), 42.3 (C-14), 39.7 (C-19), 39.7 (C-8), 38.8 (C-20), 38.4 (C-1), 37.8 (C-4), 37.4 (C-22), 37.0 (C-10), 36.2 (C-31), 32.8 (C-7), 30.9 (C-21), 28.2 (C-23), 27.9 (C-15), 24.7 (C-16), 23.6 (C-2), 23.5 (C-27), 23.4 (C-11), 23.3 (C-34, C-34′), 21.4 (Ac), 21.3 (C-30), 18.3 (C-6), 17.2 (C-29), 17.1 (C-26), 16.8 (C-24), 15.6 (C-25) ppm; MS (ESI, MeOH): *m*/*z* = 595 (100%, [M + H]^+^); analysis calcd for C_38_H_62_N_2_O_3_ (594.93): C 76.72, H 10.50, N 4.71; found: C 76.60, H 10.72, N 4.59.

*(3β)-N-(2-Piperidin-1-ylethyl)-3-acetyloxy-urs-12-en-28-*amide (**6**). Compound **6** was prepared from **1** according to general procedure B using 1-(2-aminoethyl)-piperidine. Column chromatography (SiO_2_, CHCl_3_/MeOH 95:5) gave **6** (yield: 83%); m.p. 124–127 °C; [α]_D_ = +34.6° (*c* 0.365, CHCl_3_); R_f_ = 0.26 (CHCl_3_/MeOH 95:5); IR (KBr): ν = 3424*br s*, 2936*s*, 2872*m*, 2854*m*, 1736*s*, 1638*s*, 1508*m*, 1456*m*, 1370*m*, 1246*s*, 1154*w*, 1128*w*, 1092*w*, 1028*m* cm^−1^; ^1^H-NMR (400 MHz, CDCl_3_): δ = 6.56–6.51 (*m*, 1H, NH), 5.30 (*t*, *J* = 3.6 Hz, 1H, 12-H), 4.49 (*dd*, *J* = 10.4, 5.8 Hz, 1H, 3-H), 3.41–3.29 (*m*, 1H, 31-H_a_), 3.24–3.12 (*m*, 1H, 31-H_b_), 2.49–2.27 (*m*, 6H, 32-H, 33-H, 33′-H), 2.03 (*s*, 3H, Ac), 2.01–1.80 (*m*, 5H, 16-H_a_, 11-H_a_, 11-H_b_, 18, 22-H_a_), 1.78–1.22 (*m*, 20H, 16-H_b_, 15-H_a_, 1-H_a_, 2-H_a_, 2-H_b_, 34-H, 34*′*-H, 9-H, 6-H_a_, 21-H_a_, 7-H_a_, 35-H, 22-H_b_, 19-H, 6-H_b_, 21-H_b_, 7-H_b_), 1.08 (*s*, 3H, 27-H), 1.14–0.96 (*m*, 3H, 1-H_b_, 15-H_b_, 20-H), 0.95–0.93 (*m*, 3H, 30-H), 0.93 (*s*, 3H, 25-H), 0.88 (*d*, *J* = 6.5 Hz, 3H, 29-H), 0.86 (*s*, 3H, 23-H), 0.85 (*s*, 3H, 24-H), 0.84–0.79 (*m*, 1H, 5-H), 0.77 (*s*, 3H, 26-H) ppm; ^13^C-NMR (101 MHz, CDCl_3_): δ = 178.0 (C-28), 171.1 (Ac), 139.5 (C-13), 125.6 (C-12), 81.0 (C-3), 57.2 (C-32), 55.4 (C-5), 54.5 (C-33, C-33′), 54.0 (C-18), 47.9 (C-17), 47.6 (C-9), 42.5 (C-14), 39.9 (C-19), 39.7 (C-8), 39.2 (C-20), 38.4 (C-1), 37.8 (C-4), 37.5 (C-22), 37.0 (C-10), 36.0 (C-31), 32.9 (C-7), 31.1 (C-21), 28.2 (C-23), 28.0 (C-15), 26.2 (C-34, C-34′), 24.9 (C-16), 24.5 (C-35), 23.7 (C-2), 23.5 (C-11), 23.4 (C-27), 21.4 (Ac), 21.4 (C-30), 18.3 (C-6), 17.5 (C-29), 17.1 (C-26), 16.9 (C-24), 15.7 (C-25) ppm; MS (ESI, MeOH): *m*/*z* = 609 (100%, [M + H]^+^); analysis calcd for C_39_H_64_N_2_O_3_ (608.95): C 76.92, H 10.59, N 4.60; found: C 76.77, H 10.79, N 4.41.

*(3β)-N-(2-Piperazin-1-ylethyl)-3-acetyloxy-urs-12-en-28-amide* (**7**). Compound **7** was prepared from **1** according to general procedure B using 1-(2-aminoethyl)-piperazine. Column chromatography (SiO_2_, CHCl_3_/MeOH 9:1) gave **7** (yield: 82%); m.p. 145–147 °C (lit.: 147–150 °C [18]); [a]_D_ = +35.9° (*c* 0.365, CHCl_3_); R_f_ = 0.29 (CHCl_3_/MeOH 9:1); IR (KBr): ν = 3441*s*, 2947*m*, 1734*m*, 1636*m*, 1458*w*, 1370*w*, 1247*m*, 1027*w* cm^−1^; ^1^H-NMR (400 MHz, CDCl_3_): δ = 6.42 (*t*, *J* = 4.6 Hz, 1H, N*H*), 5.30 (*t*, *J* = 3.6 Hz, 1H, 12-H), 4.49 (*dd*, *J* = 10.4, 5.3 Hz, 1H, 3-H), 3.42–3.32 (*m*, 1H, 31-H_a_), 3.23–3.13 (*m*, 1H, 31-H_b_), 2.93 (*t*, *J* = 4.9 Hz, 4H, 34-H, 34′-H), 2.49–2.37 (*m*, 6H, 32-H, 33-H, 33′-H), 2.04 (*s*, 3H, Ac), 2.02–1.80 (*m*, 5H, 16-H_a_, 11-H_a_, 11-H_b_, 22-H_a_, 18-H), 1.80–1.22 (*m*, 14H, 16-H_b_, 15-H_a_, 1-H_a_, 2-H_a_, 2-H_b_, 9-H, 6-H_a_, 21-H_a_, 7-H_a_, 22-H_b_, 19-H, 6-H_b_, 21-H_b_, 7-H_b_), 1.08 (*s*, 3H, 27-H), 1.07–0.94 (*m*, 3H, 1-H_b_, 15-H_b_, 20-H), 0.96–0.94 (*m*, 3H, 30-H), 0.93 (*s*, 3H, 25-H), 0.88 (*d*, *J* = 6.5 Hz, 3H, 29-H), 0.86 (*s*, 3H, 23-H), 0.85 (*s*, 3H, 24-H), 0.84–0.78 (*m*, 1H, 5-H), 0.77 (*s*, 3H, 26-H) ppm; ^13^C-NMR (101 MHz, CDCl_3_): δ = 178.0 (C-28), 171.1 (Ac), 139.7 (C-13), 125.5 (C-12), 81.0 (C-3), 57.1 (C-32), 55.4 (C-5), 54.1 (C-18), 53.9 (C-33), 47.9 (C-17), 47.6 (C-9), 46.1 (C-34), 42.6 (C-14), 39.9 (C-19), 39.7 (C-8), 39.3 (C-20), 38.4 (C-1), 37.8 (C-4), 37.5 (C-22), 37.0 (C-10), 35.8 (C-31), 32.8 (C-7), 31.1 (C-21), 28.2 (C-23), 28.0 (C-15), 25.0 (C-16), 23.7 (C-2), 23.6 (C-11), 23.4 (C-27), 21.4 (Ac), 21.3 (C-30), 18.3 (C-6), 17.5 (C-29), 17.1 (C-26), 16.9 (C-24), 15.7 (C-25) ppm; MS (ESI, MeOH): *m*/*z* = 610 (100 %, [M + H]^+^); analysis calcd for C_38_H_63_N_3_O_3_ (609.94): C 74.83, H 10.41, N 6.89; found: C 74.57, H 10.69, N 6.64.

*(3β)-N-(4-Aminobutyl)-3-acetyloxy-urs-12-en-28-amide* (**8**). Compound **8** was prepared from **1** according to general procedure B using 1,4-diaminobutane. Column chromatography (SiO_2_, CHCl_3_/MeOH/NH_4_OH 90:10:1) gave **8** (yield: 79%); m.p. 117–120 °C; [α]_D_ = +33.7° (*c* 0.305, CHCl_3_); R_f_ = 0.34 (CHCl_3_/MeOH/NH_4_OH 90:10:1); IR (KBr): ν = 3440*s*, 2928*m*, 1736*w*, 1636*m*, 1522*w*, 1458*w*, 1370*w*, 1246*m*, 1028*w* cm^−1^; ^1^H-NMR (400 MHz, CDCl_3_): δ = 6.03 (*t*, *J* = 5.5 Hz, 1H, NH), 5.30 (*t*, *J* = 3.6 Hz, 1H, 12-H), 4.53–4.46 (*dd*, *J* = 9.7, 6.2 Hz, 1H, 3-H), 3.41–3.30 (*m*, 1H, 31-H_a_), 3.05–2.94 (*m*, 1H, 31-H_b_), 2.78 (*t*, *J* = 6.2 Hz, 2H, 32-H_a_, 32-H_b_), 2.04 (*s*, 3H, Ac), 2.02–1.90 (*m*, 3H, 11-H_a_, 11-H_b_, 16-H_a_), 1.90–1.80 (*m*, 2H, 18-H, 22-H_a_), 1.76–1.20 (*m*, 18H, 16-H_b_, 15-H_a_, 1-H_a_, 2-H_a_, 2-H_b_, 9-H, 6-H_a_, 34-H_a_, 34-H_b_, 33-H_a_, 33-H_b_, 21-H_a_, 7-H_a_, 19-H, 22-H_b_, 6-H_b_, 21-H_b_, 7-H_b_), 1.09 (*s*, 3H, 27-H), 1.14–1.01 (*m*, 2H, 1-H_b_, 15-H_b_), 0.95 (*s*, 7H, 25-H, 30-H, 20-H), 0.89–0.86 (*m*, 3H, 29-H), 0.86 (*s*, 3H, 23-H), 0.85 (*s*, 3H, 24-H), 0.84–0.80 (*m*, 1H, 5-H), 0.77 (*s*, 3H, 26-H) ppm; ^13^C-NMR (101 MHz, CDCl_3_): δ = 178.2 (C-28), 171.1 (Ac), 140.2 (C-13), 125.6 (C-12), 81.0 (C-3), 55.4 (C-5), 54.1 (C-18), 47.8 (C-17), 47.6 (C-9), 42.7 (C-14), 41.8 (C-32), 39.9 (C-19), 39.7 (C-8), 39.4 (C-31), 39.3 (C-20), 38.5 (C-1), 37.8 (C-4), 37.4 (C-22), 37.0 (C-10), 32.8 (C-7), 31.2 (C-21), 30.8 (C-33), 28.2 (C-23), 28.0 (C-15), 26.9 (C-34), 25.0 (C-16), 23.7 (C-2), 23.6 (C-11), 23.4 (C-27), 21.5 (Ac), 21.4 (C-30), 18.3 (C-6), 17.4 (C-29), 17.1 (C-26), 16.9 (C-24), 15.7 (C-25) ppm; MS (ESI, MeOH): *m*/*z* = 569 (100%, [M + H]^+^); analysis calcd for C_36_H_60_N_2_O_3_ (568.89): C 76.01, H 10.63, N 4.92; found: C 75.84, H 10.91, N 4.63.

*(3β)-N-[2-(2-Aminoethoxy)ethyl]-3-acetyloxy-urs-12-en-28-amide* (**9**). Compound **9** was prepared from **1** according to general procedure B using 2,2′-oxybis(ethylamine). Column chromatography (SiO_2_, CHCl_3_/MeOH/NH_4_OH 90:10:0.1) gave **9** (yield: 78%); m.p. 91–94 °C; [α]_D_ = +18.3° (*c* 0.310, CHCl_3_); R_f_ = 0.39 (CHCl_3_/MeOH 9:1); IR (KBr): ν = 3424*br s*, 2927*s*, 2871*s*, 1735*s*, 1640*s*, 1529*m*, 1455*m*, 1370*m*, 1247*s*, 1120*m*, 1027*m* cm^−1^; ^1^H-NMR (500 MHz, CDCl_3_): δ = 6.29 (*t*, *J* = 5.0 Hz, 1H, NH), 5.28 (*t*, *J* = 3.6 Hz, 1H, 12-H), 4.48 (*dd*, *J* = 10.9, 5.3 Hz, 1H, 3-H), 3.57–3.44 (*m*, 5H, 31-H_a_, 32-H, 33-H), 3.30–3.22 (*m*, 1H, 31-H_b_), 2.87 (*t*, J = 5.3 Hz, 2H, 34-H), 2.03 (*s*, 3H, Ac), 2.01–1.81 (*m*, 5H, 16-H_a_, 11-H_a_, 11-H_b_, 18-H, 22-H_a_), 1.78–1.22 (*m*, 14H, 16-H_b_, 15-H_a_, 1-H_a_, 2-H_a_, 2-H_b_, 9-H, 6-H_a_, 21-H_a_, 7-H_a_, 22-H_b_, 19-H, 6-H_b_, 21-H_b_, 7-H_b_), 1.08 (*s*, 3H, 27-H), 1.12–1.00 (*m*, 2H, 1-H_b_, 15-H_b_), 0.99–0.90 (*m*, 4H, 30-H, 20-H), 0.93 (*s*, 3H, 25-H), 0.86 (*d*, *J* = 6.6 Hz, 3H, 29-H), 0.85 (*s*, 3H, 23-H), 0.84 (*s*, 3H, 24-H), 0.83–0.80 (*m*, 1H, 5-H), 0.78 (*s*, 3H, 26-H) ppm; ^13^C-NMR (126 MHz, CDCl_3_): δ = 178.3 (C-28), 171.1 (Ac), 139.8 (C-13), 125.7 (C-12), 81.0 (C-3), 73.2 (C-33), 69.6 (C-32), 55.4 (C-5), 53.9 (C-18), 47.9 (C-17), 47.6 (C-9), 42.6 (C-14), 42.0 (C-34), 39.9 (C-19), 39.7 (C-8), 39.3 (C-31), 39.2 (C-20), 38.4 (C-1), 37.8 (C-4), 37.3 (C-22), 37.0 (C-10), 32.8 (C-7), 31.0 (C-21), 28.2 (C-23), 28.0 (C-15), 25.0 (C-16), 23.7 (C-2), 23.6 (C-11), 23.4 (C-27), 21.4 (Ac), 21.4 (C-30), 18.3 (C-6), 17.4 (C-29), 17.0 (C-26), 16.8 (C-24), 15.7 (C-25) ppm; MS (ESI, MeOH): *m*/*z* = 585 (100 %, [M + H]^+^); analysis calcd for C_36_H_60_N_2_O_4_ (584.89): C 73.93, H 10.34, N 4.79; found: C 73.77, H 10.51, N 4.56.

*(3β)-N-(2-Aminoethyl)-3-hydroxy-urs-12-en-28-amide* (**10**). Compound **10** was prepared from **3** according to general procedure C. Column chromatography (SiO_2_, CHCl_3_/MeOH/NH_4_OH 90:10:0.1) gave **10** (yield: 85%); m.p. 139–142 °C (lit.: 145–147 °C [17]); [α]_D_ = +38.6° (*c* 0.300, CHCl_3_); R_f_ = 0.34 (CHCl_3_/MeOH 9:1); IR (KBr): ν = 3425*br s*, 2926*s*, 1638*m*, 1529*m*, 1454*m*, 1386*w*, 1092*w*, 1046*m*, 755*m* cm^−1^; ^1^H-NMR (400 MHz, CDCl_3_): δ = 6.36 (*t*, *J* = 5.4 Hz, 1H, N*H*), 5.33 (*t*, *J* = 3.4 Hz, 1H, 12-H), 3.46–3.36 (*m*, 1H, 31-H_a_), 3.21 (*dd*, *J* = 11.1, 4.7 Hz, 1H, 3-H), 3.13–3.02 (*m*, 1H, 31-H_b_), 2.82 (*t*, *J* = 5.9 Hz, 2H, 32-H_a_, 32-H_b_), 2.05–1.82 (*m*, 5H, 16-H_a_, 11-H_a_, 11-H_b_, 18-H, 22-H_a_), 1.77–1.23 (*m*, 14H, 16-H_b_, 15-H_a_, 1-H_a_, 2-H_a_, 2-H_b_, 9-H, 6-H_a_, 21-H_a_, 7-H_a_, 22-H_b_, 19-H, 6-H_b_, 21-H_b_, 7-H_b_), 1.09 (*s*, 3H, 27-H), 1.07–0.99 (*m*, 2H, 15-H_b_, 1-H_b_), 0.98 (*s*, 3H, 23-H), 0.96–0.93 (*m*, 4H, 20-H, 30-H), 0.91 (*s*, 3H, 25-H), 0.87 (*d*, *J* = 6.5 Hz, 3H, 29-H), 0.78 (*s*, 6H, 24-H, 26-H), 0.74–0.69 (*m*, 1H, 5-H) ppm; ^13^C-NMR (101 MHz, CDCl_3_): *δ* = 178.8 (C-28), 139.7 (C-13), 125.9 (C-12), 79.1 (C-3), 55.3 (C-5), 53.9 (C-18), 48.0 (C-17), 47.7 (C-9), 42.6 (C-14), 41.8 (C-31), 41.3 (C-32), 39.9 (C-19), 39.7 (C-8), 39.2 (C-20), 38.9 (C-4), 38.8 (C-1), 37.5 (C-22), 37.1 (C-10), 32.9 (C-7), 31.1 (C-21), 28.3 (C-23), 28.0 (C-15), 27.4 (C-2), 25.0 (C-16), 23.6 (C-11), 23.4 (C-27), 21.4 (C-30), 18.4 (C-6), 17.4 (C-29), 17.1 (C-26), 15.8 (C-24), 15.7 (C-25) ppm; MS (ESI, MeOH): *m*/*z* = 499 (100 %, [M + H]^+^); analysis calcd for C_32_H_54_N_2_O_2_ (498.80): C 77.06, H 10.91, N 5.62; found: C 76.92, H 11.08, N 5.40.

*(3β)-N-[2-(Dimethylamino)ethyl]-3-hydroxy-urs-12-en-28-amide* (**11**). Compound **11** was prepared from **4** according to general procedure C. Column chromatography (SiO_2_, CHCl_3_/MeOH 95:5) gave **11** (yield: 86%). m.p. 270–273 °C (decomp.); [α]_D_ = +38.5° (*c* 0.375, CHCl_3_); R_f_ = 0.44 (CHCl_3_/MeOH 9:1); IR (KBr): ν = 3402*br s*, 2924*s*, 2868*s*, 2684*m*, 1658*s*, 1518*m*, 1460*s*, 1386*m*, 1212*w*, 1138*w*, 1046*m*, 1028*m*, 994*m* cm^−1^; ^1^H-NMR (500 MHz, CDCl_3_): δ = 7.18 (*dd*, *J* = 5.6, 5.6 Hz, 1H, NH), 5.41 (*t*, *J* = 3.6 Hz, 1H, 12-H), 3.78–3.68 (*m*, 1H, 31-H_a_), 3.59–3.49 (*m*, 1H, 31-H_b_), 3.21 (*dd*, *J* = 11.0, 4.7 Hz, 1H, 3-H), 3.18–3.13 (*m*, 2H, 32-H), 2.84 (*s*, 6H, 33-H, 33′-H), 2.20 (*d*, *J* = 10.5 Hz, 1H, 18-H), 2.03 (*ddd*, *J* = 13.7, 13.7, 4.2 Hz, 1H, 16-H_a_), 1.96–1.91 (*m*, 2H, 11-H_a_, 11-H_b_), 1.81–1.26 (*m*, 15H, 22-H_a_, 16-H_b_, 15-H_a_, 1-H_a_, 2-H_a_, 2-H_b_, 6-H_a_, 9-H, 22-H_b_, 7-H_a_, 21-H_a_, 19-H, 6-H_b_, 7-H_b_, 21-H_b_), 1.08 (*s*, 3H, 27-H), 1.07–0.96 (*m*, 3H, 15-H_b_, 20-H, 1-H_b_), 0.98 (*s*, 3H, 23-H), 0.93 (*d*, *J* = 6.5 Hz, 3H, 30-H), 0.90 (*s*, 3H, 25-H), 0.88 (*d*, *J* = 6.5 Hz, 3H, 29-H), 0.77 (*s*, 3H, 24-H), 0.73 (*s*, 3H, 26-H), 0.72–0.69 (*m*, 1H, 5-H) ppm; ^13^C-NMR (126 MHz, CDCl_3_): δ = 179.6 (C-28), 138.8 (C-13), 126.2 (C-12), 79.1 (C-3), 58.2 (C-32), 55.3 (C-5), 52.7 (C-18), 48.0 (C-17), 47.7 (C-9), 44.2 (C-33), 43.9 (C-33′), 42.3 (C-14), 39.7 (C-8), 39.7 (C-19), 38.9 (C-1), 38.8 (C-20), 38.7 (C-4), 37.4 (C-22), 37.1 (C-10), 35.1 (C-31), 32.9 (C-7), 31.0 (C-21), 28.3 (C-23), 28.0 (C-15), 27.4 (C-2), 24.6 (C-16), 23.6 (C-27), 23.5 (C-11), 21.3 (C-30), 18.4 (C-6), 17.2 (C-29), 17.2 (C-26), 15.8 (C-24), 15.6 (C-25) ppm; MS (ESI, MeOH): *m*/*z* = 527 (100%, [M + H]^+^); analysis calcd for C_34_H_58_N_2_O_2_ (526.85): C 77.51, H 11.10, N 5.32; found: C 77.37, H 11.25, N 5.17.

*(3β)-N-(2-Pyrrolidin-1-ylethyl)-3-hydroxy-urs-12-en-28-amide* (**12**). Compound **12** was prepared from **5** according to general procedure C. Column chromatography (SiO_2_, CHCl_3_/MeOH 95:5) gave **12** (yield: 84%); m.p. 263–266 °C (decomp.); [α]_D_ = +39.8° (*c* 0.445, MeOH); R_f_ = 0.40 (CHCl_3_/MeOH 9:1); IR (KBr): ν = 3420*br s*, 2926*s*, 2670*s*, 2616*m*, 2488*m*, 2360*s*, 2342*m*, 1636*m*, 1526*m*, 1456*m*, 1386*m*, 1278*w*, 1244*w*, 1092*w*, 1046*m*, 998*m*, 668*m* cm^−1^; ^1^H-NMR (500 MHz, CDCl_3_): δ = 7.06 (*t*, *J* = 5.5 Hz, 1H, NH), 5.40 (*t*, *J* = 3.5 Hz, 1H, 12-H), 3.89–3.77 (*m*, 2H, 33-H_a_, 33′-H_a_), 3.76–3.66 (*m*, 1H, 31-H_a_), 3.61–3.51 (*m*, 1H, 31-H_b_), 3.27–3.14 (*m*, 3H, 3-H, 32-H), 2.89–2.77 (*m*, 2H, 33-H_b_, 33′-H_b_), 2.26–2.17 (*m*, 2H, 34-H_a_, 34′-H_a_), 2.14 (*d*, *J* = 11.0 Hz, 1H, 18-H), 2.12–1.97 (*m*, 3H, 34-H_b_, 34′-H_b_, 16-H_a_), 1.94 (*dd*, *J* = 8.8, 3.4 Hz, 2H, 11-H_a_, 11-H_b_), 1.81–1.22 (*m*, 15H, 22-H_a_, 16-H_b_, 15-H_a_, 1-H_a_, 2-H_a_, 2-H_b_, 6-H_a_, 9-H, 22-H_b_, 7-H_a_, 21-H_a_, 19-H, 6-H_b_, 21-H_b_, 7-H_b_), 1.08 (*s*, 3H, 27-H), 1.07–0.96 (*m*, 3H, 15-H_b_, 20-H, 1-H_b_), 0.98 (*s*, 3H, 23-H), 0.94 (*d*, *J* = 6.3 Hz, 3H, 30-H), 0.91 (*s*, 3H, 25-H), 0.88 (*d*, *J* = 6.4 Hz, 3H, 29-H), 0.78 (*s*, 3H, 24-H), 0.73 (*s*, 3H, 26-H), 0.73–0.69 (*m*, 1H, 5-H) ppm; ^13^C-NMR (126 MHz, CDCl_3_): δ = 179.6 (C-28), 138.9 (C-13), 126.2 (C-12), 79.2 (C-3), 55.4 (C-32), 55.3 (C-5), 54.9 (C-33), 54.6 (C-33′), 52.9 (C-18), 47.9 (C-17), 47.7 (C-9), 42.4 (C-14), 39.8 (C-19), 39.7 (C-8), 38.9 (C-1), 38.9 (C-20), 38.7 (C-4), 37.4 (C-22), 37.1 (C-10), 36.1 (C-31), 32.9 (C-7), 31.0 (C-21), 28.3 (C-23), 28.0 (C-15), 27.4 (C-2), 24.7 (C-16), 23.6 (C-27), 23.5 (C-34, C-34′), 23.4 (C-11), 21.4 (C-30), 18.5 (C-6), 17.3 (C-29), 17.2 (C-26), 15.8 (C-24), 15.6 (C-25) ppm; MS (ESI, MeOH): *m*/*z* = 553 (100%, [M + H]^+^); analysis calcd for C_36_H_60_N_2_O_2_ (552.89): C 78.21, H 10.94, N 5.07; found: C78.02, H 11.09, N 4.83.

*(3β)-N-(2-Piperidin-1-ylethyl)-3-hydroxy-urs-12-en-28-amide* (**13**). Compound **13** was prepared from **6** according to general procedure C. Column chromatography (SiO_2_, CHCl_3_/MeOH 95:5) gave **13** (yield: 93%); m.p. 120–124 °C; [α]_D_ = +40.5° (*c* 0.350, CHCl_3_); R_f_ = 0.23 (CHCl_3_/MeOH 95:5); IR (KBr): ν = 3416*br s*, 2934*s*, 2870*m*, 2854*m*, 1636*s*, 1512*m*, 1456*m*, 1378*m*, 1358*w*, 1304*w*, 1272*w*, 1256*w*, 1156*w*, 1130*w*, 1092*w*, 1046*m*, 998*m*, 754*m* cm^−1^; ^1^H-NMR (400 MHz, CDCl_3_): δ = 6.62–6.54 (*m*, 1H, NH), 5.31 (*t*, J = 3.6 Hz, 1H, 12-H), 3.42–3.32 (*m*, 1H, 31-H_a_), 3.25–3.16 (*m*, 2H, 3-H, 31-H_b_), 2.50–2.35 (*m*, 6H, 32-H, 33-H, 33′-H), 2.02–1.80 (*m*, 5H, 16-H_a_, 11-H_a_, 11-H_b_, 18-H, 22-H_a_), 1.78–1.20 (*m*, 20H, 16-H_b_, 15-H_a_, 1-H_a_, 34-H, 34′-H, 35-H, 9-H, 6-H_a_, 21-H_a_, 7-H_a_, 2-H_a_, 2-H_b_, 22-H_b_, 19-H, 6-H_b_, 21-H_b_, 7-H_b_), 1.08 (*s*, 3H, 27-H), 1.07–0.97 (*m*, 3H, 15-H_b_, 1-H_b_, 20-H), 0.98 (*s*, 3H, 23-H), 0.96–0.93 (*m*, 3H, 30-H), 0.91 (*s*, 3H, 25-H), 0.87 (*d*, J = 6.4 Hz, 3H, 29-H), 0.77 (*s*, 6H, 24-H, 26-H), 0.74–0.69 (*m*, 1H, 5-H) ppm; ^13^C-NMR (101 MHz, CDCl_3_): *δ* = 178.0 (C-28), 139.4 (C-13), 125.8 (C-12), 79.1 (C-3), 57.2 (C-32), 55.3 (C-5), 54.4 (C-33, C-33′), 53.9 (C-18), 47.7 (C-17), 47.7 (C-9), 42.5 (C-14), 39.9 (C-19), 39.7 (C-8), 39.2 (C-20), 38.9 (C-1), 38.8 (C-4), 37.5 (C-22), 37.1 (C-10), 35.9 (C-31), 32.9 (C-7), 31.1 (C-21), 28.3 (C-23), 28.0 (C-15), 27.3 (C-35), 26.0 (C-34, C-34′), 24.9 (C-16), 24.4 (C-2), 23.5 (C-11), 23.4 (C-27), 21.4 (C-30), 18.4 (C-6), 17.4 (C-29), 17.1 (C-26), 15.8 (C-24), 15.6 (C-25) ppm; MS (ESI, MeOH): *m*/*z* = 567 (100%, [M + H]^+^); analysis calcd for C_37_H_62_N_2_O_2_ (566.92): C 78.39, H 11.02, N 4.94; found: C 78.11, H 11.19, N 4.80.

*(3β)-N-(2-Piperazin-1-ylethyl)-3-hydroxy-urs-12-en-28-amide* (**14**). Compound **14** was prepared from **7** according to general procedure C. Column chromatography (SiO_2_, CHCl_3_/MeOH 9:1) gave **14** (yield: 88%); m.p. 214–217 °C (lit.: 217–220 °C [18]); [α]_D_ = +32.2° (*c* 0.335, CHCl_3_); R_f_ = 0.20 (CHCl_3_/MeOH 9:1); IR (KBr): ν = 3420*br s*, 2928*s*, 2870*m*, 2496*w*, 1630*s*, 1528*m*, 1458*m*, 1384*s*, 1178*w*, 1138*w*, 1092*w*, 1046*m*, 1030*m*, 998*m* cm^−1^; ^1^H-NMR (400 MHz, CD_3_OD): *δ* = 5.37 (*t*, *J* = 3.6 Hz, 1H, 12-H), 3.42–3.31 (*m*, 1H, 31-H_a_), 3.28–3.18 (*m*, 2H, 31-H_b_, 3-H), 3.16 (*t*, *J* = 5.0 Hz, 4H, 34-H, 34‘-H), 2.77–2.61 (*m*, 4H, 33-H, 33‘-H), 2.54 (*t*, *J* = 6.6 Hz, 2H, 32-H), 2.17–1.89 (*m*, 4H, 18-H, 16-H_a_, 11-H_a_, 11-H_b_), 1.86–1.30 (*m*, 15H, 15-H_a_, 22-H_a_, 16-H_b_, 1-H_a_, 2-H_a_, 2-H_b_, 9-H, 6-H_a_, 7-H_a_, 22-H_b_, 21-H_a_, 19-H, 6-H_b_, 21-H_b_, 7-H_b_), 1.16 (*s*, 3H, 27-H), 1.15–1.02 (*m*, 3H, 15-H_b_, 1-H_b_, 20-H), 1.02–0.97 (*m*, 3H, 30-H), 1.01 (*s*, 3H, 23-H), 0.99 (*s*, 3H, 25-H), 0.95 (*d*, *J* = 6.4 Hz, 3H, 29-H), 0.84 (*s*, 3H, 26-H), 0.81 (*s*, 3H, 24-H), 0.80–0.76 (*m*, 1H, 5-H) ppm; ^13^C-NMR (101 MHz, CD_3_OD): δ = 180.3 (C-28), 140.1 (C-13), 127.0 (C-12), 79.6 (C-3), 57.8 (C-32), 56.7 (C-5), 54.3 (C-18), 51.8 (C-33, C-33‘), 49.0 (C-17), 48.9 (C-9), 45.3 (C-34, C-34‘), 43.4 (C-14), 40.9 (C-8), 40.9 (C-19), 40.3 (C-20), 39.9 (C-1), 39.8 (C-4), 38.8 (C-22), 38.1 (C-10), 37.4 (C-31), 34.1 (C-7), 31.9 (C-21), 29.0 (C-15), 28.8 (C-23), 27.9 (C-2), 25.3 (C-16), 24.5 (C-11), 24.0 (C-27), 21.6 (C-30), 19.4 (C-6), 18.0 (C-26), 17.7 (C-29), 16.4 (C-24), 16.1 (C-25) ppm; MS (ESI, MeOH): *m*/*z* = 568 (100%, [M + H]^+^); analysis calcd for C_36_H_61_N_3_O_2_ (567.90): C 76.14, H 10.83, N 7.40; found: C 75.84, H 11.03, N 7.25.

*(3β)-N-(4-Aminobutyl)-3-hydroxy-urs-12-en-28-amide* (**15**). Compound **15** was prepared from **8** according to general procedure C. Column chromatography (SiO_2_, CHCl_3_/MeOH 9:1) gave **15** (yield: 87%); m.p. 177–180 °C; [α]_D_ = +42.2° (*c* 0.315, DMSO); R_f_ = 0.33 (CHCl_3_/MeOH 88:12); IR (KBr): ν = 3424*br m*, 2927*m*, 1629*w*, 1534*w*, 1384*s*, 1029*w* cm^−1^; ^1^H-NMR (400 MHz, DMSO-*d*_6_): *δ* = 7.79–7.54 (*m*, 2H, NH_2_), 7.17 (*t*, *J* = 5.7 Hz, 1H, NH), 5.19 (*t*, *J* = 3.6 Hz, 1H, 12-H), 4.28 (*s*, 1H, OH), 3.07–2.90 (*m*, 3H, 3-H, 31-H), 2.76 (*t*, *J* = 7.4 Hz, 2H, 32-H), 2.15 (*d*, *J* = 10.9 Hz, 1H, 18-H), 1.97–1.65 (*m*, 4H, 16-H_a_, 11-H_a_, 11-H_b_, 15-H_a_), 1.65–1.17 (*m*, 18H, 16-H_b_, 22-H_a_, 1-H_a_, 34-H_a_, 34-H_b_, 6-H_a_, 9-H, 2-H_a_, 2-H_b_, 7-H_a_, 22-H_b_, 21-H_a_, 33-H_a_, 33-H_b_, 19-H, 6-H_b_, 21-H_b_, 7-H_b_), 1.03 (s, 3H, 27-H), 0.95–0.88 (*m*, 6H, 15-H_b_, 1-H_b_, 30-H, 20-H), 0.89 (*s*, 3H, 23-H), 0.85 (*s*, 3H, 25-H), 0.82 (*d*, *J* = 6.4 Hz, 3H, 29-H), 0.67 (*s*, 6H, 24-H, 26-H), 0.67–0.64 (*m*, 1H, 5-H) ppm; ^13^C-NMR (101 MHz, DMSO-*d_6_*): *δ* = 176.2 (C-28), 138.4 (C-13), 124.5 (C-12), 76.8 (C-3), 54.8 (C-5), 51.9 (C-18), 47.0 (C-9), 46.5 (C-17), 41.6 (C-14), 39.1 (C-8), 38.8 (C-19), 38.7 (C-32), 38.5 (C-20), 38.4 (C-4), 38.2 (C-1), 38.0 (C-31), 37.1 (C-22), 36.5 (C-10), 32.7 (C-7), 30.4 (C-21), 28.3 (C-23), 27.4 (C-15), 27.0 (C-2), 26.0 (C-33), 24.6 (C-34), 23.5 (C-16), 23.3 (C-27), 22.9 (C-11), 21.1 (C-30), 18.0 (C-6), 17.1 (C-29), 16.8 (C-26), 16.1 (C-24), 15.2 (C-25) ppm; MS (ESI, MeOH): *m*/*z* = 527 (100 %, [M + H]^+^), 1053 (18 %, [2M + H]^+^); analysis calcd for C_34_H_58_N_2_O_2_ (526.85): C 77.51, H 11.10, N 5.32; found: C 77.39, H 11.30, N 5.16.

*(3β)-N-[2-(2-Aminoethoxy)ethyl]-3-acetyloxy-urs-12-en-28-amide* (**16**). Compound **16** was prepared from **9** according to general procedure C. Column chromatography (SiO_2_, CHCl_3_/MeOH 9:1) gave **16** (yield: 86%). m.p. 126–129 °C; [α]_D_ = +34.3° (*c* 0.305, CHCl_3_); R_f_ = 0.20 (CHCl_3_/MeOH 9:1); IR (KBr): ν = 3426*br s*, 2926*s*, 2870*m*, 1636*m*, 1534*m*, 1534*m*, 1456*w*, 1384*w*, 1118*w*, 1044*w*, 1030*w* cm^−1^; ^1^H-NMR (500 MHz, CD_3_OD): *δ* = 5.33 (*t*, *J* = 3.7 Hz, 1H, 12-H), 3.62 (*dd*, *J* = 5.7, 4.7 Hz, 2H, 33-H), 3.52 (*t*, *J* = 5.9 Hz, 2H, 32-H), 3.44–3.37 (*m*, 1H, 31-H_a_), 3.34–3.24 (*m*, 1H, 31-H_b_), 3.16 (*dd*, *J* = 11.3, 4.7 Hz, 1H, 3-H), 3.03 (*dd*, *J* = 5.7, 4.6 Hz, 2H, 34-H), 2.16–1.91 (*m*, 4H, 18-H, 16-H_a_, 11-H_a_, 11-H_b_), 1.84–1.27 (*m*, 15H, 15-H_a_, 22-H_a_, 1-H_a_, 16-H_b_, 2-H_a_, 2-H_b_, 9-H, 6-H_a_, 7-H_a_, 22-H_b_, 21-H_a_, 19-H, 6-H_b_, 21-H_b_, 7-H_b_), 1.13 (*s*, 3H, 27-H), 1.11–0.98 (*m*, 3H, 15-H_b_, 1-H_b_, 20-H), 0.98–0.95 (*m*, 3H, 30-H), 0.98 (*s*, 3H, 23-H), 0.96 (*s*, 3H, 25-H), 0.91 (*d*, *J* = 6.4 Hz, 3H, 29-H), 0.81 (*s*, 3H, 26-H), 0.78 (*s*, 3H, 24-H), 0.77–0.73 (*m*, 1H, 5-H) ppm; ^13^C-NMR (126 MHz, CD_3_OD): *δ* = 180.6 (C-28), 140.0 (C-13), 127.1 (C-12), 79.6 (C-3), 70.8 (C-32), 68.9 (C-33), 56.7 (C-5), 54.2 (C-18), 49.2 (C-17), 48.9 (C-9), 43.3 (C-14), 40.9 (C-8), 40.9 (C-34), 40.8 (C-19), 40.3 (C-20), 40.2 (C-31), 40.0 (C-1), 39.8 (C-4), 38.8 (C-22), 38.1 (C-10), 34.2 (C-7), 31.9 (C-21), 29.0 (C-15), 28.8 (C-23), 27.9 (C-2), 25.3 (C-16), 24.4 (C-11), 24.0 (C-27), 21.6 (C-30), 19.4 (C-6), 17.9 (C-29), 17.7 (C-26), 16.4 (C-24), 16.1 (C-25) ppm; MS (ESI, MeOH): *m*/*z* = 543 (100%, [M + H]^+^); analysis calcd for C_34_H_58_N_2_O_3_ (542.85): C 75.23, H 10.77, N 5.16; found: C 75.02, H 10.98, N 5.02.

*(3β)-N-(2-Aminoethyl)-3-acetyloxy-lup-20(29)-en-28-amide* (**17**). Compound **17** was prepared from **2** according to general procedure B using ethylenediamine. Column chromatography (SiO_2_, CHCl_3_/MeOH 9:1) gave **17** (yield: 83%); m.p. 152–154 °C; [α]_D_ = +8.4° (*c* 0.330, CHCl_3_); R_f_ = 0.38 (CHCl_3_/MeOH 9:1); IR (KBr): ν = 3442*br s*, 2946*s*, 1734*m*, 1638*m*, 1522*m*, 1452*m*, 1376*m*, 1248*s*, 1030*m* cm^−1^; ^1^H-NMR (400 MHz, CDCl_3_): *δ* = 6.39 (*t*, *J* = 5.5 Hz, 1H, N*H*), 4.73–4.70 (*m*, 1H, 29-H_a_), 4.60–4.57 (*m*, 1H, 29-H_b_), 4.45 (*dd*, *J* = 10.0, 6.2 Hz, 1H, 3-H), 3.40–3.33 (*m*, 2H, 31-H), 3.09 (*ddd*, *J* = 11.0, 11.0, 4.0 Hz, 1H, 19-H), 2.90 (*t*, *J* = 5.9 Hz, 2H, 32-H), 2.42 (*ddd*, *J* = 12.7, 12.7, 3.4 Hz, 1H, 13-H), 2.03 (*s*, 3H, Ac), 2.01–1.68 (*m*, 4H, 16-H_a_, 21-H_a_, 22-H_a_,12-H_a_), 1.67 (*s*, 3H, 30-H_a_), 1.66–1.07 (*m*, 16H, 22-H_b_, 2-H_a_, 2-H_b_, 18-H, 16-H_b_, 15-H_a_, 6-H_a_, 1-H_a_, 11-H_a_, 6-H_b_, 21-H_b_, 7-H_a_, 7-H_b_, 9-H, 11-H_b_, 15-H_b_), 1.05–0.88 (*m*, 2H, 12-H_b_, 1-H_b_), 0.95 (*s*, 3H, 27-H), 0.92 (*s*, 3H, 26-H), 0.83 (*s*, 6H, 25-H, 23-H), 0.82 (*s*, 3H, 24-H), 0.80–0.74 (*m*, 1H, 5-H) ppm; ^13^C-NMR (101 MHz, CDCl_3_): *δ* = 177.2 (C-28), 171.1 (Ac), 150.9 (C-20), 109.6 (C-29), 81.1 (C-3), 55.9 (C-17), 55.6 (C-5), 50.7 (C-9), 50.3 (C-18), 47.0 (C-19), 42.6 (C-14), 41.5 (C-32), 40.9 (C-8), 40.8 (C-31), 38.6 (C-1, C-22), 37.9 (C-4), 37.9 (C-13), 37.3 (C-10), 34.5 (C-7), 33.8 (C-16), 31.1 (C-21), 29.6 (C-15), 28.1 (C-23), 25.7 (C-12), 23.8 (C-2), 21.5 (Ac), 21.1 (C-11), 19.6 (C-30), 18.3 (C-6), 16.6 (C-24), 16.4 (C-25), 16.3 (C-26), 14.8 (C-27) ppm; MS (ESI, MeOH): *m*/*z* = 541 (100 %, [M + H]^+^); analysis calcd for C_34_H_56_N_2_O_3_ (540.83): C 75.51, H 10.44, N 5.18; found: C 75.35, H 10.67, N 5.02.

*(3β)-N-[2-(Dimethylamino)ethyl]-3-acetyloxy-lup-20(29)-en-28-amide* (**18**). Compound **18** was prepared from **2** according to general procedure B using *N*,*N*-dimethylethylenediamine. Column chromatography (SiO_2_, CHCl_3_/MeOH 95:5) gave **18** (yield: 94%); m.p. 108–110 °C; [α]_D_ = +16.4° (*c* 0.320, CHCl_3_); R_f_ = 0.51 (CHCl_3_/MeOH 9:1); IR (KBr): ν = 3420*br s*, 2945*s*, 2869*m*, 1736*s*, 1641*m*, 1456*s*, 1375*m*, 1246*s*, 1195*w*, 1029*m*, 979*m* cm^−1^; ^1^H-NMR (500 MHz, CDCl_3_): *δ* = 6.24 (*t*, *J* = 4.7 Hz, 1H, NH), 4.74–4.72 (*m*, 1H, 29-H_a_), 4.60–4.58 (*m*, 1H, 29-H_b_), 4.46 (*dd*, *J* = 10.4, 5.9 Hz, 1H, 3-H), 3.40–3.24 (*m*, 2H, 31-H), 3.11 (*ddd*, *J* = 11.0, 11.0, 4.2 Hz, 1H, 19-H), 2.47–2.38 (*m*, 3H, 32-H + 13-H), 2.26 (*s*, 6H, 33-H, 33′-H), 2.03 (*s*, 3H, Ac), 2.03–1.89 (*m*, 2H, 16-H_a_, 21-H_a_), 1.80–1.74 (m, 1H, 22-H_a_), 1.73–1.63 (*m*, 2H, 12-H_a_, 22-H_b_), 1.68 (*s*, 3H, 30-H), 1.63–1.11 (*m*, 15H, 2-H_a_, 2-H_b_, 18-H, 16-H_b_, 15-H_a_, 6-H_a_, 11-H_a_, 1-H_a_, 6-H_b_, 7-H_a_, 7-H_b_, 21-H_b_, 9-H, 11-H_b_, 15-H_b_), 1.05–0.94 (*m*, 2H, 12-H_b_, 1-H_b_), 0.96 (*s*, 3H, 27-H), 0.94 (*s*, 3H, 26-H), 0.83 (*s*, 6H, 23-H, 25-H), 0.82 (*s*, 3H, 24-H), 0.80–0.76 (*m*, 1H, 5-H) ppm; ^13^C-NMR (126 MHz, CDCl_3_): *δ* = 176.5 (C-28), 171.1 (Ac), 151.2 (C-20), 109.5 (C-29), 81.1 (C-3), 58.3 (C-32), 55.9 (C-17), 55.6 (C-5), 50.7 (C-9), 50.2 (C-18), 47.1 (C-19), 45.3 (C-33, C-33′), 42.7 (C-14), 40.9 (C-8), 38.6 (C-22), 38.6 (C-1), 38.0 (C-13), 38.0 (C-4), 37.3 (C-10), 36.6 (C-31), 34.5 (C-7), 33.8 (C-16), 31.1 (C-21), 29.6 (C-15), 28.1 (C-23), 25.8 (C-12), 23.9 (C-2), 21.5 (Ac), 21.1 (C-11), 19.6 (C-30), 18.4 (C-6), 16.6 (C-24), 16.4 (C-25), 16.3 (C-26), 14.8 (C-27) ppm; MS (ESI, MeOH): *m*/*z* = 569 (100%, [M + H]^+^); analysis calcd for C_26_H_60_N_2_O_3_ (568.89): C 76.01, H 10.63, N 4.92; found: C 75.77, H 10.84, N 4.63.

*(3β)-N-(2-Pyrrolidin-1-ylethyl)-3-acetyloxy-lup-20(29)-en-28-amide* (**19**). Compound **19** was prepared from **2** according to general procedure B using 1-(2-aminoethyl)-pyrrolidine. Column chromatography (SiO_2_, CHCl_3_/MeOH 95:5) gave **19** (yield: 86%); m.p. 143–145 °C; [α]_D_ = +11.7° (*c* 0.330, CHCl_3_); R_f_ = 0.53 (CHCl_3_/MeOH 9:1); IR (KBr): ν =3422*br m*, 2946*s*, 1734*m*, 1640*m*, 1451*m*, 1384*s*, 1247*s*, 1029*m*, 979*m* cm^−1^; ^1^H-NMR (400 MHz, CDCl_3_): *δ* = 7.23 (*t*, *J* = 5.5 Hz, 1H, NH), 4.72–4.70 (*m*, 1H, 29-H_a_), 4.58–4.56 (*m*, 1H, 29-H_b_), 4.44 (*dd*, *J* = 10.8, 5.5 Hz, 1H, 3-H), 3.68–3.56 (*m*, 2H, 31-H), 3.47–3.27 (*m*, 4H, 33-H, 33′-H), 3.24 (*t*, *J* = 6.1 Hz, 2H, 32-H), 3.05 (*ddd*, *J* = 10.9, 10.9, 4.2 Hz, 1H, 19-H), 2.39 (*ddd*, *J* = 12.8, 12.8, 3.5 Hz, 1H, 13-H), 2.13–2.04 (*m*, 5H, 34-H, 34′-H, 16-H_a_), 2.02 (*s*, 3H, Ac), 1.89–1.76 (*m*, 2H, 21-H_a_, 22-H_a_), 1.66 (*s*, 3H, 30-H), 1.71–1.11 (*m*, 17H, 12-H_a_, 1-H_a_, 2-H_a_, 2-H_b_, 18-H, 16-H_b_, 6-H_a_, 22-H_b_, 11-H_a_, 21-H_b_, 7-H_a_, 7-H_b_, 15-H_a_, 6-H_b_, 9-H, 11-H_b_, 15-H_b_), 1.03–0.91 (*m*, 2H, 12-H_b_, 1-H_b_), 0.93 (*s*, 3H, 27-H), 0.89 (*s*, 3H, 26-H), 0.82 (*s*, 6H, 23-H, 25-H), 0.81 (*s*, 3H, 24-H), 0.78–0.75 (*m*, 1H, 5-H) ppm; ^13^C-NMR (101 MHz, CDCl_3_): *δ* = 177.8 (C-28), 171.1 (Ac), 151.0 (C-20), 109.6 (C-29), 81.1 (C-3), 55.9 (C-17), 55.6 (C-5), 55.5 (C-32), 54.8 (C-33, C-33′), 50.6 (C-9), 50.3 (C-18), 47.0 (C-19), 42.6 (C-14), 40.9 (C-8), 38.5 (C-1), 38.2 (C-22), 37.9 (C-13), 37.9 (C-4), 37.3 (C-10), 36.3 (C-31), 34.5 (C-7), 33.2 (C-16), 31.0 (C-21), 29.6 (C-15), 28.1 (C-23), 25.7 (C-12), 23.8 (C-2), 23.4 (C-34, C-34′), 21.4 (Ac), 21.1 (C-11), 19.5 (C-30), 18.3 (C-6), 16.6 (C-24), 16.3 (C-25), 16.3 (C-26), 14.7 (C-27) ppm; MS (ESI, MeOH): *m*/*z* = 595 (100%, [M + H]^+^); analysis calcd for C_38_H_62_N_2_O_3_ (594.93): C 76.72, H 10.50, N 4.71; found: C 76.50, H 10.74, N 4.51.

*(3β)-N-(2-Piperidin-1-ylethyl)-3-acetyloxy-lup-20(29)-en-28-amide* (**20**). Compound **20** was prepared from **2** according to general procedure B using 1-(2-aminoethyl)-piperidine. Column chromatography (SiO_2_, CHCl_3_/MeOH 95:5) gave **20** (yield: 81%); m.p. 124–127 °C; [α]_D_ = +14.1° (*c* 0.340, CHCl_3_); R_f_ = 0.25 (CHCl_3_/MeOH 95:5); IR (KBr): ν = 3424*br s*, 2942*s*, 2968*m*, 1736*s*, 1638*s*, 1508*m*, 1452*m*, 1376*m*, 1246*s*, 1154*w*, 1128*w*, 1028*m* cm^−1^; ^1^H-NMR (400 MHz, CDCl_3_): *δ* = 6.52–6.39 (*m*, 1H, NH), 4.75–4.70 (*m*, 1H, 29-H_a_), 4.61–4.56 (*m*, 1H, 29-H_b_), 4.46 (*dd*, *J* = 9.8, 6.5 Hz, 1H, 3-H), 3.41–3.25 (*m*, 2H, 31-H_a_, 31-H_b_), 3.08 (*ddd*, *J* = 11.1, 10.9, 3.9 Hz, 1H, 19-H), 2.53–2.40 (*m*, 6H, 32-H, 33-H, 33′-H), 2.35 (*ddd*, *J* = 12.4, 12.3, 3.6 Hz, 1H, 13-H), 2.03 (*s*, 3H, Ac), 2.12–1.89 (*m*, 2H, 16-H_a_, 21-H_a_), 1.83–1.74 (*m*, 1H, 22-H_a_), 1.68 (*s*, 3H, 30-H), 1.72–1.54 (*m*, 9H, 12-H_a_, 1-H_a_, 2-H_a_, 2-H_b_, 18-H, 34-H, 34′-H), 1.55–1.15 (*m*, 13H, 16-H_b_, 15-H_a_, 6-H_a_, 35-H, 11-H_a_, 22-H_b_, 21-H_b_, 6-H_b_, 7-H_a_, 7-H_b_, 9-H, 11-H_b_), 1.15–1.09 (*m*, 1H, 15-H_b_), 1.08–0.93 (*m*, 2H, 12-H_b_, 1-H_b_), 0.96 (*s*, 3H, 27-H), 0.92 (*s*, 3H, 26-H), 0.83 (*s*, 6H, 25-H, 23-H), 0.82 (*s*, 3H, 24-H), 0.80–0.74 (*m*, 1H, 5-H) ppm; ^13^C-NMR (101 MHz, CDCl_3_): *δ* = 176.3 (C-28), 171.1 (Ac), 151.1 (C-20), 109.5 (C-29), 81.1 (C-3), 57.1 (C-32), 56.0 (C-17), 55.6 (C-5), 54.3 (C-33, C-33′), 50.6 (C-9), 50.0 (C-18), 47.2 (C-19), 42.7 (C-14), 40.9 (C-8), 38.5 (C-1), 38.5 (C-22), 38.1 (C-13), 37.9 (C-4), 37.3 (C-10), 35.7 (C-31), 34.5 (C-7), 33.8 (C-16), 31.1 (C-21), 29.6 (C-15), 28.1 (C-23), 26.1 (C-34, C-34′), 25.8 (C-12), 24.4 (C-35), 23.8 (C-2), 21.4 (Ac), 21.1 (C-11), 19.6 (C-30), 18.4 (C-6), 16.6 (C-24), 16.4 (C-25), 16.3 (C-26), 14.8 (C-27) ppm; MS (ESI, MeOH): *m*/*z* = 609 (100%, [M + H]^+^); analysis calcd for C_39_H_64_N_2_O_3_ (608.95): C 76.92, H 10.59, N 4.60; found:

*(3β)-N-(2-Piperazin-1-ylethyl)-3-acetyloxy-lup-20(29)-en-28-amide* (**21**). Compound **21** was prepared from **2** according to general procedure B using 1-(2-aminoethyl)-piperazine. Column chromatography (SiO_2_, CHCl_3_/MeOH/NH_4_OH 90:11:0.5) gave **21** (yield: 84%); m.p. 107–110 °C; [α]_D_ = +13.7° (*c* 0.335, CHCl_3_); R_f_ = 0.44 (CHCl_3_/MeOH/NH_4_OH 90:10:1);IR (KBr): ν = 3422*br s*, 2944*s*, 2870*m*, 1734*s*, 1638*s*, 1522*m*, 1452*m*, 1374*m*, 1318*w*, 1248*s*, cm^−1^; 1194*w*, 1138*w*, 1028*m* cm^−1^; ^1^H-NMR (400 MHz, CDCl_3_): *δ* = 6.22 (*t*, *J* = 4.8 Hz, 1H, NH), 4.75–4.70 (*m*, 1H, 29-H_a_), 4.61–4.58 (*m*, 1H, 29-H_b_), 4.46 (*dd*, *J* = 9.7, 6.5 Hz, 1H, 3-H), 3.41–3.23 (*m*, 2H, 31-H), 3.08 (*ddd*, *J* = 11.1, 11.1, 3.8 Hz, 1H, 19-H), 2.92 (*t*, *J* = 4.9 Hz, 4H, 34-H, 34′-H), 2.55–2.41 (*m*, 6H, 32-H, 33-H, 33′-H), 2.36 (*ddd*, *J* = 12.4, 12.4, 3.6 Hz, 1H, 13-H), 2.03 (s, 3H, Ac), 2.05–1.89 (*m*, 2H, 16-H_a_, 21-H_a_), 1.68 (*s*, 3H, 30-H), 1.81–1.53 (*m*, 6H, 22-H_a_, 12-H_a_, 1-H_a_, 18-H, 2-H_a_, 2-H_b_), 1.52–1.19 (*m*, 11H, 16-H_b_, 15-H_a_, 6-H_a_, 11-H_a_, 22-H_b_, 6-H_b_, 21-H_b_, 7-H_a_, 7-H_b_, 9-H, 11-H_b_), 1.20–0.90 (*m*, 3H, 15-H_b_, 12-H_b_, 1-H_b_), 0.96 (*s*, 3H, 27-H), 0.91 (*s*, 3H, 26-H), 0.83 (*s*, 6H, 23-H, 25-H), 0.82 (*s*, 3H, 24-H), 0.80–0.74 (*m*, 1H, 5-H) ppm; ^13^C-NMR (101 MHz, CDCl_3_): *δ* = 176.3 (C-28), 171.1 (Ac), 151.0 (C-20), 109.6 (C-29), 81.1 (C-3), 57.1 (C-32), 56.0 (C-17), 55.6 (C-5), 53.7 (C-33, C-33′), 50.6 (C-9), 50.0 (C-18), 47.2 (C-19), 46.1 (C-34, C-34′), 42.7 (C-14), 40.9 (C-8), 38.5 (C-1, C-22), 38.1 (C-13), 37.9 (C-4), 37.3 (C-10), 35.6 (C-31), 34.5 (C-7), 33.8 (C-16), 31.1 (C-21), 29.6 (C-15), 28.1 (C-23), 25.7 (C-12), 23.8 (C-2), 21.5 (Ac), 21.1 (C-11), 19.6 (C-30), 18.3 (C-6), 16.6 (C-24), 16.4 (C-25), 16.3 (C-26), 14.8 (C-27) ppm; MS (ESI, MeOH): *m*/*z* = 610 (100%, [M + H]^+^); analysis calcd for C_38_H_63_N_3_O_3_ (609.94): C 74.83, H 10.41, N 6.89; found: C 74.65, H 10.69, N 6.64.

*(3β)-N-(4-Aminobutyl)-3-acetyloxy-lup-20(29)-en-28-amide* (**22**). Compound **22** was prepared from **2** according to general procedure B using 1,4-diaminobutane. Column chromatography (SiO_2_, CHCl_3_/MeOH/NH_4_OH 90:11:0.5) gave **22** (yield: 84%); m.p. 133–135 °C; [α]_D_ = +7.4° (*c* 0.350, CHCl_3_); R_f_ = 0.33 (CHCl_3_/MeOH/NH_4_OH 90:10:1); IR (KBr): ν = 3422*br s*, 2944*s*, 2868*m*, 1736*s*, 1638*s*, 1522*m*, 1452*m*, 1374*m*, 1248*s*, 1028*m*, 980*m*, 754*m* cm^−1^; ^1^H-NMR (400 MHz, CDCl_3_): *δ* = 5.94 (*t*, *J* = 5.8 Hz, 1H, NH), 4.75–4.69 (*m*, 1H, 29-H_a_), 4.61–4.55 (*m*, 1H, 29-H_b_), 4.45 (*dd*, *J* = 8.7, 7.6 Hz, 1H, 3-H), 3.35–3.15 (*m*, 2H, 31-H_a_, 31-H_b_), 3.11 (*ddd*, *J* = 11.0, 4.0 Hz, 4.0 Hz, 1H, 19-H), 2.84–2.73 (*m*, 2H, 32-H_a_, 32-H_b_), 2.45 (*ddd*, *J* = 12.4, 3.6 Hz, 3.6 Hz, 1H, 13-H), 2.03 (*s*, 3H, Ac), 2.00–1.80 (*m*, 2H, 21-H_a_, 16-H_a_), 1.67 (*s*, 3H, 30-H), 1.77–1.18 (*m*, 21H, 22-H_a_, 12-H_a_, 1-H_a_, 2-H_a_, 2-H_b_, 34-H_a_, 34-H_b_, 18-H, 33-H_a_, 33-H_b_, 16-H_b_, 6-H_a_, 15-H_a_, 11-H_a_, 22-H_b_, 6-H_b_, 21-H_b_, 7-H_a_, 7-H_b_, 11-H_b_, 9-H), 1.16–1.08 (*m*, 1H, 15-H_b_), 1.04–0.90 (*m*, 2H, 12-H_b_, 1-H_b_), 0.95 (*s*, 3H, 27-H), 0.92 (*s*, 3H, 26-H), 0.84 (*s*, 3H, 25-H), 0.83 (*s*, 3H, 23-H), 0.82 (*s*, 3H, 24-H), 0.80–0.74 (*m*, 1H, 5-H) ppm; ^13^C-NMR (101 MHz, CDCl_3_): *δ* = 176.4 (C-28), 171.1 (Ac), 151.1 (C-20), 109.5 (C-29), 81.1 (C-3), 55.8 (C-17), 55.6 (C-5), 50.7 (C-9), 50.3 (C-18), 46.9 (C-19), 42.6 (C-14), 41.4 (C-32), 40.9 (C-8), 39.1 (C-31), 38.6 (C-22), 38.5 (C-1), 38.0 (C-4), 37.9 (C-13), 37.3 (C-10), 34.5 (C-7), 33.9 (C-16), 31.1 (C-21), 29.9 (C-33), 29.6 (C-15), 28.1 (C-23), 27.3 (C-34), 25.8 (C-12), 23.9 (C-2), 21.5 (Ac), 21.1 (C-11), 19.6 (C-30), 18.3 (C-6), 16.6 (C-24), 16.4 (C-25), 16.3 (C-26), 14.8 (C-27) ppm; MS (ESI, MeOH): *m*/*z* = 569 (100%, [M + H]^+^); analysis calcd for C_36_H_60_N_2_O_3_ (568.89): C 76.01, H 10.63, N 4.92; found: C75.81, H 10.77, N 4.75.

*(3β)-N-[2-(2-Aminoethoxy)ethyl]-3-acetyloxy-lup-20(29)-en-28-amide* (**23**). Compound **23** was prepared from **2** according to general procedure B using 2,2′-oxybis(ethylamine). Column chromatography (SiO_2_, CHCl_3_/MeOH 9:1) gave **23** (yield: 81%); m.p. 109–112 °C; [α]_D_ = +38.4° (*c* 0.325, CHCl_3_); R_f_ = 0.58 (CHCl_3_/MeOH/NH_4_OH 90:10:1); IR (KBr): ν = 3448*br s*, 2944*m*, 1734*m*, 1637*m*, 1527*w*, 1375w, 1248*m*, 1029*w* cm^−1^; ^1^H-NMR (500 MHz, CDCl_3_): *δ* = 6.09 (*t*, *J* = 5.4 Hz, 1H, NH), 4.74–4.70 (*m*, 1H, 29-H_a_), 4.60–4.56 (*m*, 1H, 29-H_b_), 4.46 (*dd*, *J* = 10.6, 5.8 Hz, 1H, 3-H), 3.58–3.44 (*m*, 5H, 32-H, 33-H, 31-H_a_), 3.43–3.35 (*m*, 1H, 31-H_b_), 3.10 (*ddd*, *J* = 11.1, 11.0, 4.2 Hz, 1H, 19-H), 2.88 (*t*, *J* = 5.2 Hz, 2H, 34-H), 2.43 (*ddd*, *J* = 12.9, 11.5, 3.7 Hz, 1H, 13-H), 2.03 (*s*, 3H, Ac), 2.00–1.88 (*m*, 2H, 16-H_a_, 21-H_a_), 1.78–1.72 (*m*, 1H, 22-H_a_), 1.67 (*s*, 3H, 30-H), 1.72–1.17 (*m*, 16H, 12-H_a_, 1-H_a_, 2-H_a_, 2-H_b_, 18-H, 16-H_b_, 6-H_a_, 15-H_a_, 22-H_b_, 11-H_a_, 6-H_b_, 21-H_b_, 7-H_a_, 7-H_b_, 9-H, 11-H_b_), 1.16–1.09 (*m*, 1H, 15-H_b_), 1.05–0.95 (*m*, 2H, 12-H_b_, 1-H_b_), 0.95 (*s*, 3H, 27-H), 0.93 (*s*, 3H, 26-H), 0.83 (*s*, 3H, 25-H), 0.83 (*s*, 3H, 23-H), 0.82 (*s*, 3H, 24-H), 0.80–0.74 (*m*, 1H, 5-H) ppm; ^13^C-NMR (126 MHz, CDCl_3_): *δ* = 176.4 (C-28), 171.1 (Ac), 151.0 (C-20), 109.5 (C-29), 81.1 (C-3), 72.8 (C-33), 70.1 (C-32), 55.9 (C-17), 55.6 (C-5), 50.7 (C-9), 50.2 (C-18), 47.0 (C-19), 42.6 (C-14), 41.8 (C-34), 40.9 (C-8), 39.1 (C-31), 38.6 (C-1), 38.5 (C-22), 37.9 (C-4), 37.9 (C-13), 37.3 (C-10), 34.5 (C-7), 33.9 (C-16), 31.0 (C-21), 29.5 (C-15), 28.1 (C-23), 25.7 (C-12), 23.9 (C-2), 21.4 (Ac), 21.1 (C-11), 19.6 (C-30), 18.4 (C-6), 16.6 (C-24), 16.3 (C-25), 16.3 (C-26), 14.8 (C-27) ppm; MS (ESI, MeOH): *m*/*z* = 585 (100 %, [M + H]^+^), 607 (47 %, [M + Na]^+^); analysis calcd for C_36_H_60_N_2_O_4_ (584.89): C 73.93, H 10.34, N 4.79; found: C 73.69, H 10.54, N 4.56.

*(3β)-N-(2-Aminoethyl)-3-hydroxy-lup-20(29)-en-28-amide* (**24**). Compound **24** was prepared from **17** according to general procedure C. Column chromatography (SiO_2_, CHCl_3_/MeOH 9:1) gave **24** (yield: 86%); m.p. 218–220 °C; [α]_D_ = +4.5° (*c* 0.300, DMSO); R_f_ = 0.28 (CHCl_3_/MeOH 9:1);IR (KBr): ν = 3424*br s*, 2941*m*, 1636*m*, 1449*m*, 1044*m*, 879*w* cm^−1^; ^1^H-NMR (400 MHz, DMSO-*d*_6_): *δ* = 7.53 (*t*, *J* = 5.5 Hz, 1H, NH), 4.67–4.63 (*m*, 1H, 29-H_a_), 4.54–4.51 (*m*, 1H, 29-H_b_), 3.15–2.92 (*m*, 4H, 32-H_a_, 19-H, 32-H_b_, 3-H), 2.60–2.51 (*m*, 3H, 13-H, 31-H_a_, 31-H_b_), 2.16–2.09 (*m*, 1H, 16-H_a_), 1.82–1.65 (*m*, 2H, 22-H_a_, 21-H_a_), 1.62 (*s*, 3H, 30-H), 1.61–0.92 (*m*, 17H, 12-H_a_, 1-H_a_, 2-H_a_, 2-H_b_, 6-H_a_, 18-H, 16-H_b_, 11-H_a_, 22-H_b_, 15-H_a_, 6-H_b_, 7-H_a_, 7-H_b_, 21-H_b_, 9-H, 11-H_b_, 15-H_b_), 0.92–0.78 (*m*, 2H, 12-H_b_, 1-H_b_), 0.91 (*s*, 3H, 27-H), 0.87 (*s*, 3H, 23-H), 0.84 (*s*, 3H, 26-H), 0.76 (*s*, 3H, 25-H), 0.65 (*s*, 3H, 24-H), 0.64–0.60 (*m*, 1H, 5-H) ppm; ^13^C-NMR (101 MHz, DMSO-*d*_6_): *δ* = 175.6 (C-28), 150.9 (C-20), 109.1 (C-29), 76.8 (C-3), 54.9 (C-5), 54.9 (C-17), 50.1 (C-9), 49.7 (C-18), 46.2 (C-19), 41.9 (C-14), 41.8 (C-32), 41.4 (C-31), 40.3 (C-8), 38.5 (C-4), 38.3 (C-1), 37.7 (C-22), 36.7 (C-10), 36.6 (C-13), 34.0 (C-7), 32.4 (C-16), 30.3 (C-21), 28.9 (C-15), 28.1 (C-23), 27.1 (C-2), 25.2 (C-12), 20.6 (C-11), 19.0 (C-30), 17.9 (C-6), 15.9 (C-25), 15.8 (C-26), 15.7 (C-24), 14.3 (C-27) ppm; MS (ESI, MeOH): *m*/*z* = 499 (100 %, [M + H]^+^); analysis calcd for C_32_H_54_N_2_O_2_ (498.80): C 77.06, H 10.91, N 5.62; found: C 76.81, H 11.07, N 5.55.

*(3β)-N-[2-(Dimethylamino)ethyl]-3-hydroxy-lup-20(29)-en-28-amide* (**25**). Compound **25** was prepared from **18** according to general procedure C. Column chromatography (SiO_2_, CHCl_3_/MeOH 95:5) gave **25** (yield: 89%); m.p. 118–120 °C; [α]_D_ = −4.4° (*c* 0.330, MeOH); R_f_ = 0.43 (CHCl_3_/MeOH 9:1); IR (KBr): ν = 3408*br s*, 2944*s*, 2866*s*, 1638*s*, 1528*m*, 1464*s*, 1376*m*, 1246*m*, 1194*m*, 1044*m*, 880*m* cm^−1^; ^1^H-NMR (500 MHz, CDCl_3_): *δ* = 6.26 (*t*, *J* = 4.9 Hz, 1H, NH), 4.73–4.71 (*m*, 1H, 29-H_a_), 4.58–4.56 (*m*, 1H, 29-H_b_), 3.37–3.22 (*m*, 2H, 31-H), 3.16 (*dd*, *J* = 11.0, 5.2 Hz, 1H, 3-H), 3.10 (*ddd*, *J* = 11.1, 11.1, 4.2 Hz, 1H, 19-H), 2.46–2.37 (*m*, 3H, 13-H, 32-H), 2.22 (*s*, 6H, 33-H, 33′-H), 2.06–1.89 (*m*, 2H, 16-H_a_, 21-H_a_), 1.79–1.72 (*m*, 1H, 22-H_a_), 1.67 (*s*, 3H, 30-H), 1.72– 1.16 (*m*, 16H, 12-H_a_, 1-H_a_, 2-H_a_, 2-H_b_, 18-H, 6-H_a_, 16-H_b_, 15-H_a_, 11-H_a_, 22-H_b_, 6-H_b_, 7-H_a_, 7-H_b_, 21-H_b_, 9-H, 11-H_b_), 1.15–1.10 (*m*, 1H, 15-H_b_), 1.04–0.96 (*m*, 1H, 12-H_b_), 0.95 (*s*, 3H, 27-H), 0.95 (*s*, 3H, 23-H), 0.93 (*s*, 3H, 26-H), 0.91–0.81 (*m*, 1H, 1-H_b_), 0.80 (*s*, 3H, 25-H), 0.74 (*s*, 3H, 24-H), 0.69–0.64 (*m*, 1H, 5-H) ppm; ^13^C-NMR (126 MHz, CDCl_3_): *δ* = 176.4 (C-28), 151.2 (C-20), 109.4 (C-29), 79.1 (C-3), 58.3 (C-32), 55.9 (C-17), 55.5 (C-5), 50.8 (C-9), 50.2 (C-18), 47.0 (C-19), 45.3 (C-33, C-33′), 42.6 (C-14), 40.9 (C-8), 39.0 (C-4), 38.9 (C-1), 38.6 (C-22), 38.0 (C-13), 37.4 (C-10), 36.7 (C-31), 34.6 (C-7), 33.8 (C-16), 31.1 (C-21), 29.6 (C-15), 28.1 (C-23), 27.6 (C-2), 25.8 (C-12), 21.1 (C-11), 19.6 (C-30), 18.5 (C-6), 16.3 (C-26), 16.2 (C-25), 15.5 (C-24), 14.8 (C-27) ppm; MS (ESI, MeOH): *m*/*z* = 527 (100%, [M + H]^+^); analysis calcd for C_34_H_58_N_2_O_2_ (526.85): C 77.51, H 11.10, N 5.32; found: C 77.40, H 11.22, N 5.18.

*(3β)-N-(2-Pyrrolidin-1-ylethyl)-3-hydroxy-lup-20(29)-en-28-amide* (**26**). Compound **26** was prepared from **19** according to general procedure C. Column chromatography (SiO_2_, CHCl_3_/MeOH 95:5) gave **26** (yield: 80%); m.p. 253–256 °C (decomp.); [α]_D_ = −14.7° (*c* 0.320, MeOH); R_f_ = 0.40 (CHCl_3_/MeOH 9:1); IR (KBr): ν = 3426*br s*, 2942*s*, 2866*s*, 2696*m*, 2620*m*, 2500*m*, 1638*s*, 1544*m*, 1450*m*, 1376*m*, 1246*w*, 1196*w*, 1046*m*, 880*m* cm^−1^; ^1^H-NMR (500 MHz, CDCl_3_): *δ* = 7.54 (*t*, *J* = 5.7 Hz, 1H, NH), 4.73–4.71 (*m*, 1H, 29-H_a_), 4.59–4.57 (*m*, 1H, 29-H_b_), 3.91–3.79 (*m*, 2H, 33-H_a_, 33′-H_a_), 3.78–3.61 (*m*, 2H, 31-H), 3.24–3.15 (*m*, 3H, 32-H, 3-H), 3.07 (*ddd*, *J* = 10.9, 10.9, 4.2 Hz, 1H, 19-H), 2.89–2.78 (*m*, 2H, 33-H_b_, 33′-H_b_), 2.42 (*ddd*, J = 12.6, 12.6, 3.6 Hz, 1H, 13-H), 2.31–2.18 (*m*, 3H, 16-H_a_, 34-H_a_, 34′-H_a_), 2.15–2.05 (*m*, 2H, 34-H_b_, 34′-H_b_), 1.96–1.78 (*m*, 2H, 22-H_a_, 21-H_a_), 1.67 (*s*, 3H, 30-H), 1.73–1.14 (*m*, 17H, 12-H_a_, 1-H_a_, 2-H_a_, 2-H_b_, 18-H, 16-H_b_, 6-H_a_, 22-H_b_, 11-H_a_, 6-H_b_, 21-H_b_, 7-H_a_, 7-H_b_, 15-H_a_, 9-H, 11-H_b_, 15-H_b_), 1.01–0.92 (*m*, 1H, 12-H_b_), 0.96 (*s*, 6H, 23-H, 27-H), 0.91 (*s*, 3H, 26-H), 0.89–0.81 (*m*, 1H, 1-H_b_), 0.81 (*s*, 3H, 25-H), 0.75 (*s*, 3H, 24-H), 0.70–0.65 (*m*, 1H, 5-H) ppm; ^13^C-NMR (126 MHz, CDCl_3_): *δ* = 177.9 (C-28), 151.1 (C-20), 109.5 (C-29), 79.1 (C-3), 56.6 (C-32), 56.1 (C-17), 55.5 (C-5), 54.8 (C-33, C-33′), 50.8 (C-9), 50.4 (C-18), 47.0 (C-19), 42.6 (C-14), 40.9 (C-8), 39.0 (C-4), 38.9 (C-1), 38.2 (C-22), 37.9 (C-13), 37.4 (C-10), 35.7 (C-31), 34.6 (C-7), 33.2 (C-16), 31.1 (C-21), 29.7 (C-15), 28.1 (C-23), 27.6 (C-2), 25.8 (C-12), 23.5 (C34, C-34′), 21.1 (C-11), 19.6 (C-30), 18.5 (C-6), 16.4 (C-26), 16.3 (C-25), 15.5 (C-24), 14.8 (C-27) ppm; MS (ESI, MeOH): *m*/*z* = 553 (100%, [M + H]^+^); analysis calcd for C_36_H_60_N_2_O_2_ (552.89): C 78.21, H 10.94, N 5.07; found: C 78.00, H 11-09, N 4.81.

*(3β)-N-(2-Piperidin-1-ylethyl)-3-hydroxy-lup-20(29)-en-28-amide* (**27**). Compound **27** was prepared from **20** according to general procedure C. Column chromatography (SiO_2_, CHCl_3_/MeOH 95:5) gave **27** (yield: 83%); m.p. 141–144 °C (decomp.); [α]_D_ = +4.9° (*c* 0.315, CHCl_3_); R_f_ = 0.21 (CHCl_3_/MeOH 95:5); IR (KBr): ν = 3424*br s*, 2940*s*, 2866*m*, 2364*w*, 1638*s*, 1508*m*, 1452*m*, 1376*m*, 1248*w*, 1194*w*, 1128*w*, 1046*m* cm^−1^; ^1^H-NMR (400 MHz, CDCl_3_): *δ* = 6.78–6.57 (*m*, 1H, NH), 4.76–4.69 (*m*, 1H, 29-H_a_), 4.61–4.56 (*m*, 1H, 29-H_b_), 3.48–3.31 (*m*, 2H, 31-H), 3.17 (*dd*, *J* = 11.1, 5.0 Hz, 1H, 3-H), 3.08 (*ddd*, *J* = 11.0, 10.8, 3.9 Hz, 1H, 19-H), 2.65–2.46 (*m*, 6H, 32-H, 33-H, 33′-H), 2.37 (*ddd*, *J* = 12.4, 12.3, 3.6 Hz, 1H, 13-H), 2.15–2.08 (*m*, 1H, 16-H_a_), 2.00–1.88 (*m*, 1H, 21-H_a_), 1.85–1.76 (*m*, 1H, 22-H_a_), 1.68 (*s*, 3H, 30-H), 1.73–1.08 (*m*, 23H, 12-H_a_, 35-H, 1-H_a_, 18-H, 34-H, 34′-H, 6-H_a_, 2-H_a_, 2-H_b_, 16-H_b_, 15-H_a_, 11-H_a_, 22-H_b_, 21-H_b_, 6-H_b_, 7-H_a_, 7-H_b_, 9-H, 11-H_b_, 15-H_b_), 1.08–0.95 (*m*, 1H, 12-H_b_), 0.96 (*s*, 3H, 27-H), 0.95 (*s*, 3H, 23-H), 0.91 (*s*, 3H, 26-H), 0.91–0.82 (*m*, 1H, 1-H_b_), 0.80 (*s*, 3H, 25-H), 0.74 (*s*, 3H, 24-H), 0.70–0.63 (*m*, 1H, 5-H) ppm; ^13^C-NMR (101 MHz, CDCl_3_): *δ* = 176.6 (C-28), 151.1 (C-20), 109.5 (C-29), 79.1 (C-3), 57.3 (C-32), 56.1 (C-17), 55.5 (C-5), 54.3 (C-33, C-33′), 50.7 (C-9), 50.1 (C-18), 47.1 (C-19), 42.7 (C-14), 40.9 (C-8), 39.0 (C-4), 38.9 (C-1), 38.4 (C-22), 38.1 (C-13), 37.4 (C-10), 35.5 (C-31), 34.6 (C-7), 33.6 (C-16), 31.1 (C-21), 29.6 (C-15), 28.1 (C-23), 27.6 (C-34, C-34′), 25.8 (C-12), 25.5 (C-35), 24.0 (C-2), 21.1 (C-11), 19.6 (C-30), 18.5 (C-6), 16.3 (C-26), 16.2 (C-25), 15.5 (C-24), 14.8 (C-27) ppm; MS (ESI, MeOH): *m*/*z* = 567 (100%, [M + H]^+^); analysis calcd for C_37_H_62_N_2_O_2_ (566.92): C 78.39, H 11.02, N 4.94; found: C 78.16, H 11.20, N 4.71.

*(3β)-N-(2-Piperazin-1-ylethyl)-3-hydroxy-lup-20(29)-en-28-amide* (**28**). Compound **28** was prepared from **21** according to general procedure C. Column chromatography (SiO_2_, CHCl_3_/MeOH/NH_4_OH 90:10:0.5) gave **28** (yield: 90%); m.p. 146–148 °C (decomp.); [α]_D_ = +6.5° (*c* 0.380, CHCl_3_); R_f_ = 0.30 (CHCl_3_/MeOH/NH_4_OH 90:10:1); IR (KBr): ν = 3422*br s*, 3072*w*, 2942*s*, 2868*m*, 1638*s*, 1510*m*, 1452*m*, 1376*m*, 1320*w*, 1248*w*, 1194*w*, 1138*w*, 1046*w*, 754*m* cm^−1^; ^1^H-NMR (400 MHz, CDCl_3_): *δ* = 6.19 (*t*, *J* = 4.9 Hz, 1H, NH), 4.77–4.70 (*m*, 1H, 29-H_a_), 4.63–4.55 (*m*, 1H, 29-H_b_), 3.42–3.28 (*m*, 2H, 31-H), 3.17 (*dd*, *J* = 11.1, 5.0 Hz, 1H, 3-H), 3.09 (*ddd*, *J* = 10.8, 10.3, 3.7 Hz, 1H, 19-H), 2.97 (*t*, *J* = 4.9 Hz, 4H, 34-H, 34′-H), 2.58–2.42 (*m*, 6H, 32-H, 33-H, 33′-H), 2.37 (*ddd*, *J* = 12.4, 12.3, 3.7 Hz, 1H, 13-H), 2.06–1.89 (*m*, 2H, 16-H_a_, 21-H_a_), 1.68 (*s*, 3H, 30-H), 1.81–1.08 (*m*, 18H, 22-H_a_, 12-H_a_, 1-H_a_, 18-H, 2-H_a_, 2-H_b_, 6-H_a_, 16-H_b_, 15-H_a_, 11-H_a_, 22-H_b_, 6-H_b_, 21-H_b_, 7-H_a_, 7-H_b_, 9-H, 11-H_b_, 15-H_b_), 1.07–0.95 (*m*, 1H, 12-H_b_), 0.97 (*s*, 3H, 27-H), 0.95 (*s*, 3H, 23-H), 0.92 (*s*, 3H, 26-H), 0.96–0.83 (*m*, 1H, 1-H_b_), 0.80 (*s*, 3H, 25-H), 0.75 (*s*, 3H, 24-H), 0.70–0.64 (*m*, 1H, 5-H) ppm; ^13^C-NMR (101 MHz, CDCl_3_): *δ* = 176.3 (C-28), 151.0 (C-20), 109.6 (C-29), 79.1 (C-3), 57.1 (C-32), 56.0 (C-17), 55.5 (C-5), 53.6 (C-33, C-33′), 50.7 (C-9), 50.1 (C-18), 47.2 (C-19), 46.0 (C-34, C-34′), 42.7 (C-14), 40.9 (C-8), 39.0 (C-4), 38.9 (C-1), 38.5 (C-22), 38.1 (C-13), 37.4 (C-10), 35.6 (C-31), 34.6 (C-7), 33.9 (C-16), 31.1 (C-21), 29.6 (C-15), 28.2 (C-23), 27.6 (C-2), 25.8 (C-12), 21.1 (C-11), 19.6 (C-30), 18.5 (C-6), 16.4 (C-26), 16.3 (C-25), 15.5 (C-24), 14.8 (C-27) ppm; MS (ESI, MeOH): *m*/*z* = 568 (100%, [M + H]^+^); analysis calcd for C_36_H_61_N_3_O_2_ (567.90): C 76.14, H 10.83, N 7.40; found: C 75.96, H 11.01, N 7.27.

*(3β)-N-(4-Aminobutyl)-3-hydroxy-lup-20(29)-en-28-amide* (**29**). Compound **29** was prepared from **22** according to general procedure C. Column chromatography (SiO_2_, CHCl_3_/MeOH 9:1) gave **29** (yield: 85%); m.p. 130–133 °C; [α]_D_ = +4.8° (*c* 0.380, DMSO); R_f_ = 0.31 (CHCl_3_/MeOH 88:12);IR (KBr): ν = 3448*br s*, 2941*s*, 2867*m*, 1636*m*, 1534*m*, 1452*m*, 1384*w*, 1195*w*, 1045*w* cm^−1^; ^1^H-NMR (400 MHz, DMSO-*d*_6_): *δ* = 7.55 (*t*, *J* = 5.8 Hz, 1H, NH), 4.67–4.62 (*m*, 1H, 29-H_a_), 4.55–4.50 (*m*, 1H, 29-H_b_), 3.12–2.88 (*m*, 4H, 31-H_a_, 19-H, 3-H, 31-H_b_), 2.61–2.51 (*m*, 3H, 13-H, 32-H_a_, 32-H_b_), 2.18–2.09 (*m*, 1H, 16-H_a_), 1.81–1.64 (*m*, 2H, 22-H_a_, 21-H_a_), 1.62 (*s*, 3H, 30-H), 1.61–1.51 (*m*, 2H, 1-H_a_, 12-H_a_), 1.49–0.98 (*m*, 19H, 2-H_a_, 2-H_b_, 6-H_a_, 18-H, 34-H_a_, 34-H_b_, 11-H_a_, 16-H_b_, 15a, 22-H_b_, 6-H_b_, 33-H_a_, 33-H_b_, 7-H_a_, 7-H_b_, 9-H, 21-H_b_, 11-H_b_, 15-H_b_), 0.97–0.78 (*m*, 2H, 1-H_b_, 12-H_b_), 0.90 (*s*, 3H, 27-H), 0.86 (*s*, 3H, 23-H), 0.84 (*s*, 3H, 26-H), 0.76 (*s*, 3H, 25-H), 0.65 (*s*, 3H, 24-H), 0.64–0.59 (*m*, 1H, 5-H) ppm; ^13^C-NMR (101 MHz, DMSO-*d*_6_): *δ* = 175.2 (C-28), 150.9 (C-20), 109.1 (C-29), 76.8 (C-3), 55.0 (C-5), 54.8 (C-17), 50.1 (C-9), 49.7 (C-18), 46.1 (C-19), 41.9 (C-14), 41.2 (C-32), 40.3 (C-8), 38.5 (C-4), 38.3 (C-31), 38.2 (C-1), 37.7 (C-22), 36.7 (C-10), 36.6 (C-13), 34.0 (C-7), 32.4 (C-16), 30.4 (C-21, C33), 28.9 (C-15), 28.1 (C-23), 27.2 (C-2), 26.7 (C-34), 25.2 (C-12), 20.6 (C-11), 19.0 (C-30), 18.0 (C-6), 16.0 (C-25), 15.8 (C-26), 15.7 (C-24), 14.3 (C-27) ppm; MS (ESI, MeOH): *m*/*z* = 527 (100 %, [M + H]^+^), 1053 (22 %, [2M + H]^+^); analysis calcd for C_34_H_58_N_2_O_2_ (526.45): C 77.51, H 11.10, N 5.32; found: C 77.38, H 11.30, N 5.13.

*(3β)-N-[2-(2-Aminoethoxy)ethyl]-3-hydroxy-lup-20(29)-en-28-amide* (**30**). Compound **30** was prepared from **23** according to general procedure C. Column chromatography (SiO_2_, CHCl_3_/MeOH 9:1) gave **30** (yield: 91%); m.p. 182–183 °C; [α]_D_ = −1.1° (*c* 0.315, MeOH); R_f_ = 0.45 (CHCl_3_/MeOH/NH_4_OH 90:10:1); IR (KBr): ν = 3424*br s*, 2942*s*, 2868*m*, 1636*s*, 1534*m*, 1450*w*, 1384*w*, 1318*w*, 1278*w*, 1248*w*, 1196*w*, 1108*m*, 1044*m* cm^−1^; ^1^H-NMR (500 MHz, CD_3_OD): *δ* = 4.72–4.68 (*m*, 1H, 29-H_a_), 4.61–4.57 (*m*, 1H, 29-H_b_), 3.70–3.63 (*m*, 2H, 33-H), 3.57–3.51 (*m*, 2H, 32-H), 3.47–3.31 (*m*, 2H, 31-H), 3.15–3.05 (*m*, 4H, 3-H, 19-H, 34-H), 2.56 (*ddd*, *J* = 12.8, 12.4, 3.6 Hz, 1H, 13-H), 2.12 (*ddd*, *J* = 13.1, 3.3, 3.2 Hz, 1H, 16-H_a_), 1.93–1.78 (*m*, 2H, 21-H_a_, 22-H_a_), 1.69 (*s*, 3H, 30-H), 1.76–1.63 (*m*, 2H, 12-H_a_, 1-H_a_), 1.65–1.10 (*m*, 15H, 2-H_a_, 2-H_b_, 18-H, 16-H_b_, 6-H_a_, 15-H_a_, 22-H_b_, 11-H_a_, 6-H_b_, 7-H_a_, 7-H_b_, 21-H_b_, 9-H, 11-H_b_, 15-H_b_), 1.09–0.96 (*m*, 1H, 12-H_b_), 1.00 (*s*, 3H, 27-H), 0.97 (*s*, 3H, 26-H), 0.95 (*s*, 3H, 23-H), 0.95–0.88 (*m*, 1H, 1-H_b_), 0.86 (*s*, 3H, 25-H), 0.75 (*s*, 3H, 24-H), 0.73–0.69 (*m*, 1H, 5-H) ppm; ^13^C-NMR (126 MHz, CD_3_OD): *δ* = 179.6 (C-28), 152.3 (C-20), 110.0 (C-29), 79.6 (C-3), 71.4 (C-32), 67.8 (C-33), 57.1 (C-17), 56.9 (C-5), 52.1 (C-9), 51.4 (C-18), 48.2 (C-19), 43.5 (C-14), 42.0 (C-8), 40.7 (C-34), 40.1 (C-1), 39.9 (C-4), 39.7 (C-31), 39.3 (C-22), 39.0 (C-13), 38.3 (C-10), 35.6 (C-7), 34.1 (C-16), 31.9 (C-21), 30.6 (C-15), 28.6 (C-23), 28.0 (C-2), 27.0 (C-12), 22.2 (C-11), 19.6 (C-30), 19.5 (C-6), 16.9 (C-24), 16.8 (C-25), 16.1 (C-26), 15.1 (C-27) ppm; MS (ESI, MeOH): *m*/*z* = 543 (100 %, [M + H]^+^), 1085 (10 %, [2M + H]^+^); analysis calcd for C_34_H_58_N_2_O_3_ (542.58): C 75.23, H 10.77, N 5.16; found: C 75.11, H 10.94, N 4.97.

## 5. Conclusions

A set of 28 ursolic and betulinic carboxamides was prepared and screened for their cytotoxic activity using SRB assays. This screening revealed the compounds derived from betulinic acid to be more potent than those from ursolic acid. In particular, betulinic carboxamides **24**–**30** showed remarkable cytotoxicity, as indicated by EC_50_ values lower than 1 µM. The most potent compounds of this study are (*3β*)-*N*-[2-(dimethylamino)ethyl]-3-hydroxy-lup-20(29)-en-28-amide (**25**, EC_50_ = 0.2 µM ± 0.01 µM for A2780 tumor cells; SI = 1.50) and (*3β*)-*N*-(2-pyrrolidin-1-ylethyl)-3-hydroxy-lup-20(29)-en-28-amide (**26**, EC_50_ = 0.2 µM ± 0.05 µM for MCF-7 tumor cells; SI = 2.00). Compound **18** showed the highest selectivity for HT29 tumor cells (EC_50_ = 0.3 ± 0.02 µM; SI = 3.33). Further structural modifications showed that the replacement of the 3-*O*-acetyl moiety has an impact on the cytotoxicity and on the selectivity, respectively. Compounds **25** and **26** were selected for extended biological testing employing MCF-7 and A2780 human tumor cell lines. Fluorescence microscopic images revealed both of the compounds to show characteristics of apoptosis.

## Figures and Tables

**Table 1:** Cytotoxicity of compounds **3–30**, betulinic acid (**BA**), ursolic acid (**UA**), and doxorubicin hydrochloride (**DRC**): EC_50_ values from SRB assays after 96 h of treatment are given in µM (n.d. not detected); the values are averaged from three independent experiments each performed in triplicate; confidence interval CI = 95%.

	518A2	A2780	HT29	MCF-7	8505C	NIH 3T3
**BA**	9.4 ± 0.70	8.8 ± 0.90	14.4 ± 2.3	10.2 ± 1.2	n.d.	13.1 ± 1.1
**UA**	14.7 ± 0.1	11.7 ± 0.6	10.6 ± 0.7	12.7 ± 0.1	13.5 ± 1.5	18.7 ± 1.6
**3**	2.7 ± 0.10	2.3 ± 0.10	1.8 ± 0.10	2.0 ± 0.10	4.1 ± 0.40	2.6 ± 0.30
**4**	5.3 ± 0.40	3.6 ± 0.40	3.4 ± 0.30	3.3 ± 0.70	8.3 ± 0.70	3.7 ± 0.20
**5**	4.5 ± 0.20	3.1 ± 0.20	2.1 ± 0.20	2.9 ± 0.70	4.3 ± 0.20	3.3 ± 0.30
**6**	12.2 ± 0.30	6.5 ± 0.50	4.2 ± 0.50	6.0 ± 0.90	15.5 ± 2.7	7.2 ± 1.20
**7**	3.2 ± 0.10	2.4 ±0.10	1.8 ± 0.20	2.7 ± 0.30	5.4 ± 0.40	2.2 ± 0.10
**8**	2.7 ± 0.10	2.6 ± 0.10	1.7 ± 0.10	1.7 ± 0.10	3.2 ± 0.01	1.3 ± 0.20
**9**	3.5 ± 0.60	3.4 ± 0.50	1.6 ± 0.10	2.3 ± 0.40	5.8 ± 0.40	3.1 ± 0.40
**10**	3.7 ± 0.50	3.3 ± 0.10	2.0 ± 0.10	3.2 ± 0.30	3.7 ± 0.40	3.9 ± 0.10
**11**	6.6 ± 0.30	4.3 ± 0.50	3.0 ± 0.20	4.2 ± 0.90	7.3 ± 0.20	6.7 ± 1.30
**12**	5.5 ± 0.10	3.3 ± 0.30	2.6 ± 0.30	3.5 ± 1.00	5.7 ± 0.20	6.2 ± 0.40
**13**	12.6 ± 0.20	7.6 ± 0.60	8.4 ± 0.70	11.1 ± 1.40	11.1 ± 0.30	3.5 ± 0.60
**14**	7.0 ± 0.40	4.9 ± 0.40	2.8 ± 0.40	4.4 ± 0.10	7.8 ± 0.50	6.6 ± 0.60
**15**	9.3 ± 0.10	5.4 ± 1.40	2.1 ± 0.30	5.5 ± 1.10	10.8 ± 1.20	5.6 ± 0.30
**16**	9.9 ± 0.20	6.5 ± 1.40	3.1 ± 0.30	6.3 ± 0.60	10.6 ± 1.10	7.5 ± 0.80
**17**	1.6 ± 0.10	1.4 ± 0.20	1.0 ± 0.30	1.3 ± 0.06	2.5 ± 0.03	1.4 ± 0.10
**18**	1.3 ± 0.10	1.1 ± 0.05	0.3 ± 0.02	1.0 ± 0.30	1.4 ± 0.15	1.0 ± 0.40
**19**	1.5 ± 0.10	1.4 ± 0.09	0.5 ± 0.08	1.2 ± 0.20	1.6 ± 0.09	1.2 ± 0.07
**20**	3.1 ± 0.30	1.6 ± 0.20	0.8 ± 0.10	1.5 ± 0.50	3.0 ± 0.36	1.2 ± 0.20
**21**	2.5 ± 0.70	1.5 ± 0.06	0.6 ± 0.30	1.9 ± 0.10	1.1 ± 0.09	1.0 ± 0.05
**22**	1.0 ± 0.03	1.1 ± 0.03	0.6 ± 0.05	0.8 ± 0.04	1.4 ± 0.05	0.6 ± 0.04
**23**	1.9 ± 0.01	1.5 ± 0.10	0.4 ± 0.10	1.4 ± 0.20	2.2 ± 0.81	1.0 ± 0.10
**24**	0.4 ± 0.01	0.4 ± 0.03	0.4 ± 0.02	0.4 ± 0.02	0.4 ± 0.07	0.4 ± 0.02
**25**	0.3 ± 0.16	0.2 ± 0.01	0.3 ± 0.05	0.3 ± 0.07	0.4 ± 0.04	0.3 ± 0.09
**26**	0.4 ± 0.06	0.3 ± 0.10	0.4 ± 0.02	0.2 ± 0.05	0.5 ± 0.06	0.4 ± 0.02
**27**	0.5 ± 0.10	0.4 ± 0.10	0.4 ± 0.03	0.4 ± 0.01	0.8 ± 0.09	0.4 ± 0.01
**28**	0.9 ± 0.21	0.8 ± 0.04	0.8 ± 0.18	0.3 ± 0.02	0.85 ± 0.17	0.8 ± 0.22
**29**	0.4 ± 0.06	0.6 ± 0.10	0.4 ± 0.10	0.6 ± 0.10	0.8 ± 0.26	0.5 ± 0.10
**30**	0.5 ± 0.04	0.5 ± 0.04	0.5 ± 0.07	0.4 ± 0.20	0.2 ± 0.01	0.4 ± 0.07
**DRC**	0.2 ± 0.05	0.01 ± 0.01	0.9 ± 0.2	1.1 ± 0.3	1.1 ± 0.4	0.06 ± 0.03

**Table 2:** Selectivity of compounds **3**–**30**, betulinic acid (**BA**), ursolic acid (**UA**) and doxorubicin hydrochloride (**DRC**): Selectivity index (SI) is defined as: SI = EC_50_ (NIH 3T3)/EC_50_ (tumor cell line).

	(518A2)	A2780	HT29	MCF-7	8505C
**BA**	1.39	1.49	0.91	1.28	-
**UA**	1.27	1.59	1.76	1.47	1.39
**3**	0.96	1.13	1.44	1.30	0.63
**4**	0.70	1.03	1.09	1.12	0.45
**5**	0.73	1.06	1.57	1.14	0.77
**6**	0.59	1.11	1.71	1.20	0.46
**7**	0.69	0.92	1.22	0.81	0.41
**8**	0.48	0.50	0.76	0.76	0.41
**9**	0.89	0.91	1.93	1.35	0.53
**10**	1.05	1.18	1.95	1.22	1.05
**11**	1.02	1.56	2.23	1.60	0.92
**12**	1.13	1.88	2.38	1.77	1.09
**13**	0.28	0.46	0.42	0.32	0.32
**14**	0.94	1.35	2.36	1.50	0.85
**15**	0.60	1.04	2.67	1.02	0.52
**16**	0.76	1.15	2.42	1.19	0.71
**17**	0.88	1.00	1.40	1.08	0.56
**18**	0.77	0.91	3.33	1.00	0.71
**19**	0.80	0.86	2.40	1.00	0.75
**20**	0.39	0.75	1.50	0.80	0.40
**21**	0.40	0.67	1.67	0.53	0.91
**22**	0.60	0.55	1.00	0.75	0.43
**23**	0.53	0.67	2.50	0.71	0.45
**24**	1.00	1.00	1.00	1.00	1.00
**25**	1.00	1.50	1.00	1.00	0.75
**26**	1.00	1.33	1.00	2.00	0.80
**27**	0.80	1.00	1.00	1.00	0.50
**28**	0.89	1.00	1.00	2.67	0.94
**29**	1.25	0.83	1.25	0.83	0.63
**30**	0.80	0.80	0.80	1.00	2.00
**DRC**	0.30	6.00	0.07	0.05	0.05

## Supplementary Materials

Supplementary data related to this article can be found at http://www.mdpi.com/1420-3049/23/10/2558/s1.
