# Supplementary material for: Ethylenediamine Derived Carboxamides of Betulinic and Ursolic Acid as Potential Cytotoxic Agents
Source: Molecules. 2018 Oct 8;23(10):2558. doi: 10.3390/molecules23102558 (PMC6222718; doi:10.3390/molecules23102558)
Supplement: Supplementary file 1 [file molecules-23-02558-s001.pdf]

Supplementary Material

Betulinic and ursolic carboxamides as potential cytotoxic agents

Michael Kahnt [1], Lucie Fischer (née Heller) [1], Ahmed Al-Harrasi [2] and René Csuk [1,*]

1 Martin-Luther-University Halle-Wittenberg, Organic Chemistry, Kurt-Mothes-Str. 2, D-06120 Halle (Saale), Germany; michael.kahnt@chemie.uni-halle.de, lucie.heller@chemie.uni-halle.de

2 University of Nizwa, Chair of Oman's Medicinal Plants and Marine Natural Products, PO Box 33, Birkat Al-Mauz, Nizwa, Sultanate of Oman; aharrasi@unizwa.edu.om

* Correspondence: rene.csuk@chemie.uni-halle.de; Tel.: +49-345-55-25660

Representative NMR spectra

NMR spectra of 3

^{1}H NMR

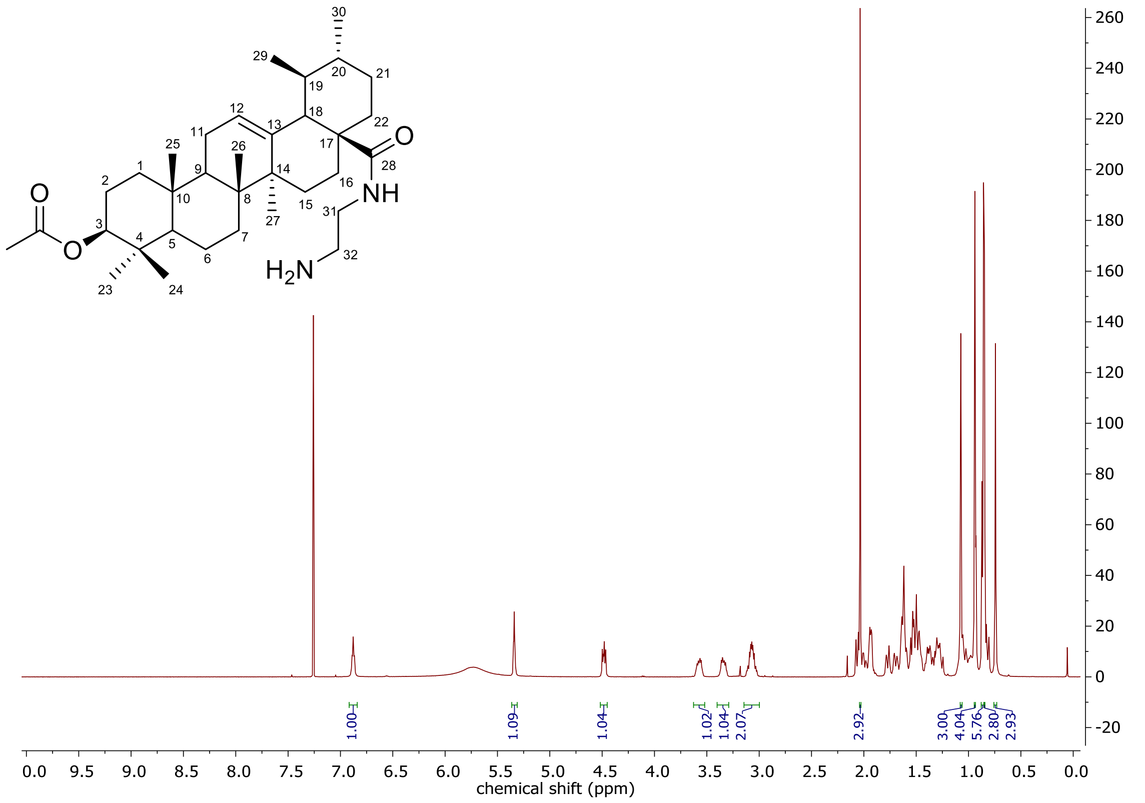

^{13}C NMR (APT)

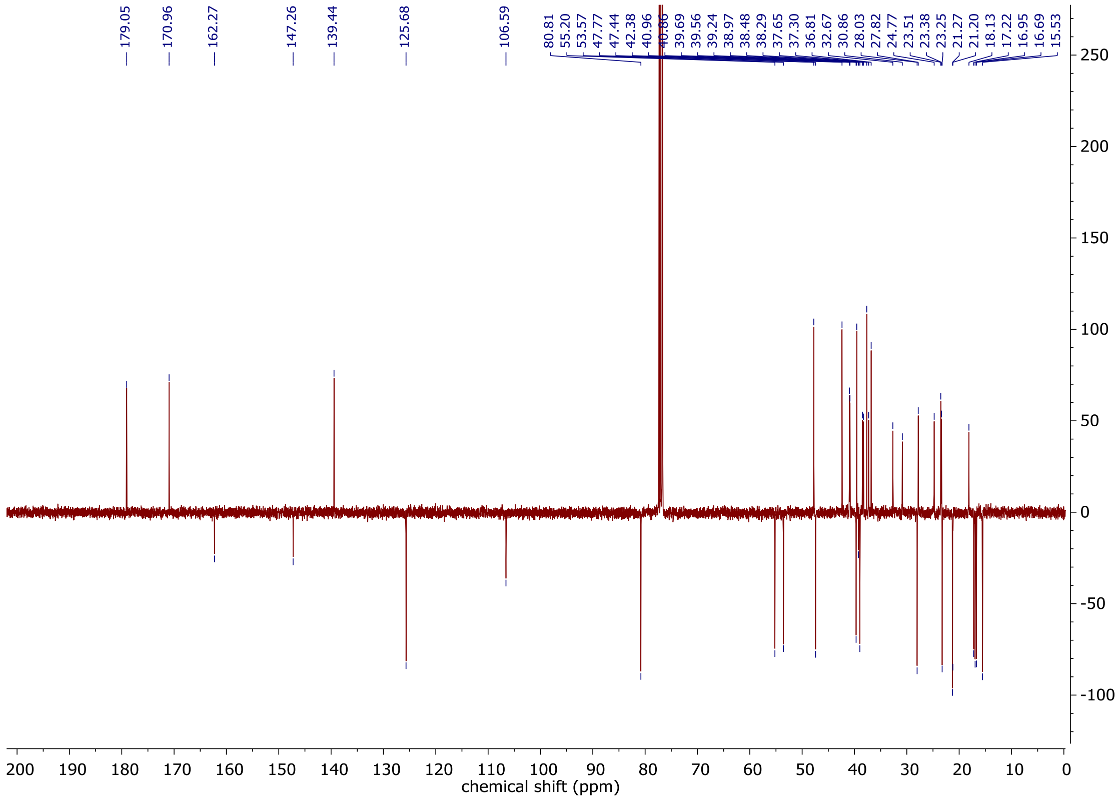

NMR spectra of 4

^{1}H NMR

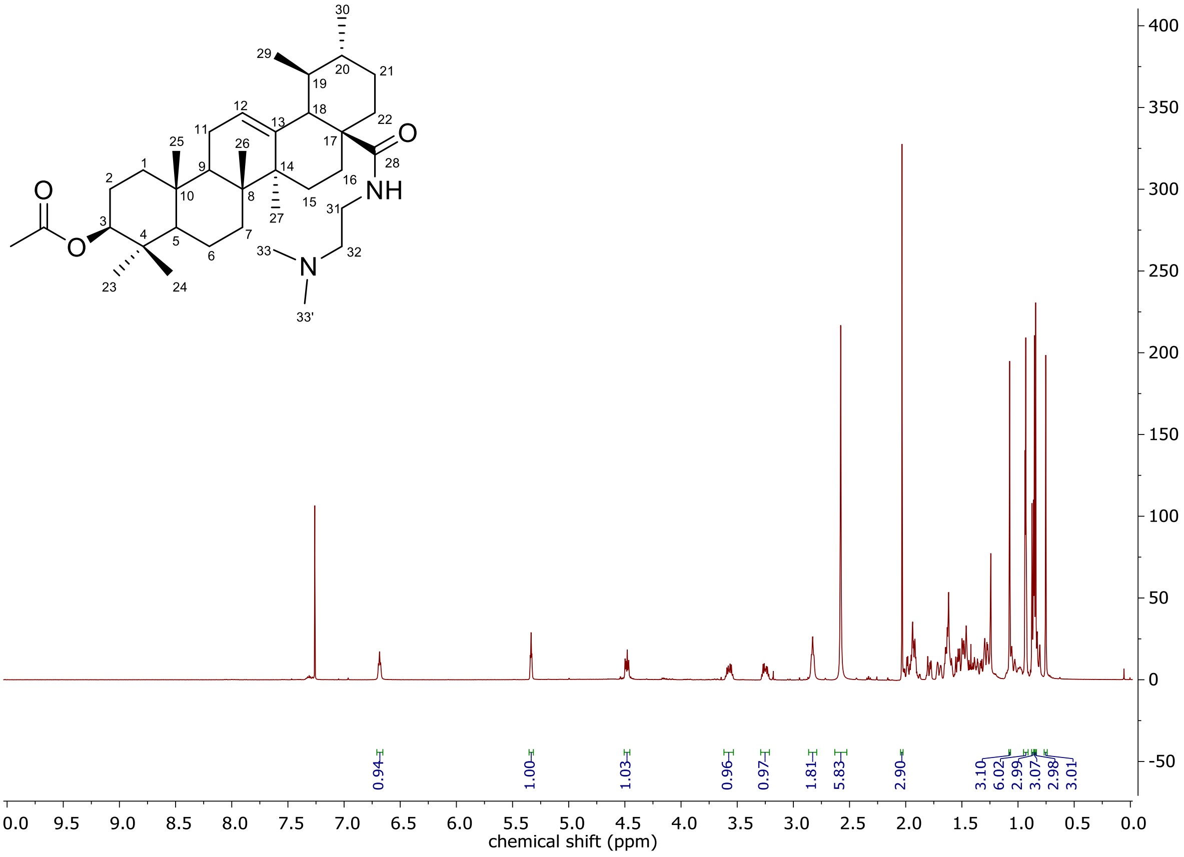

^{13}C NMR (APT)

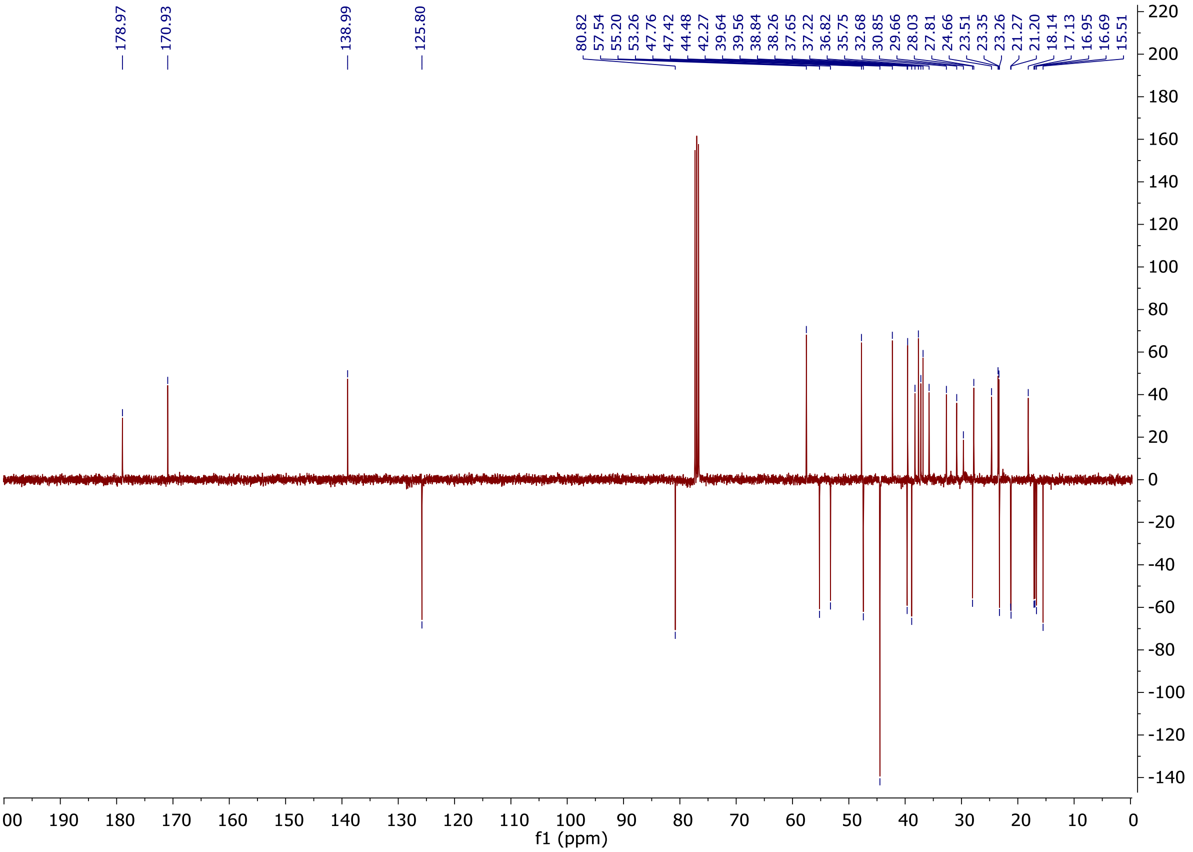

NMR spectra of 5

^{1}H NMR

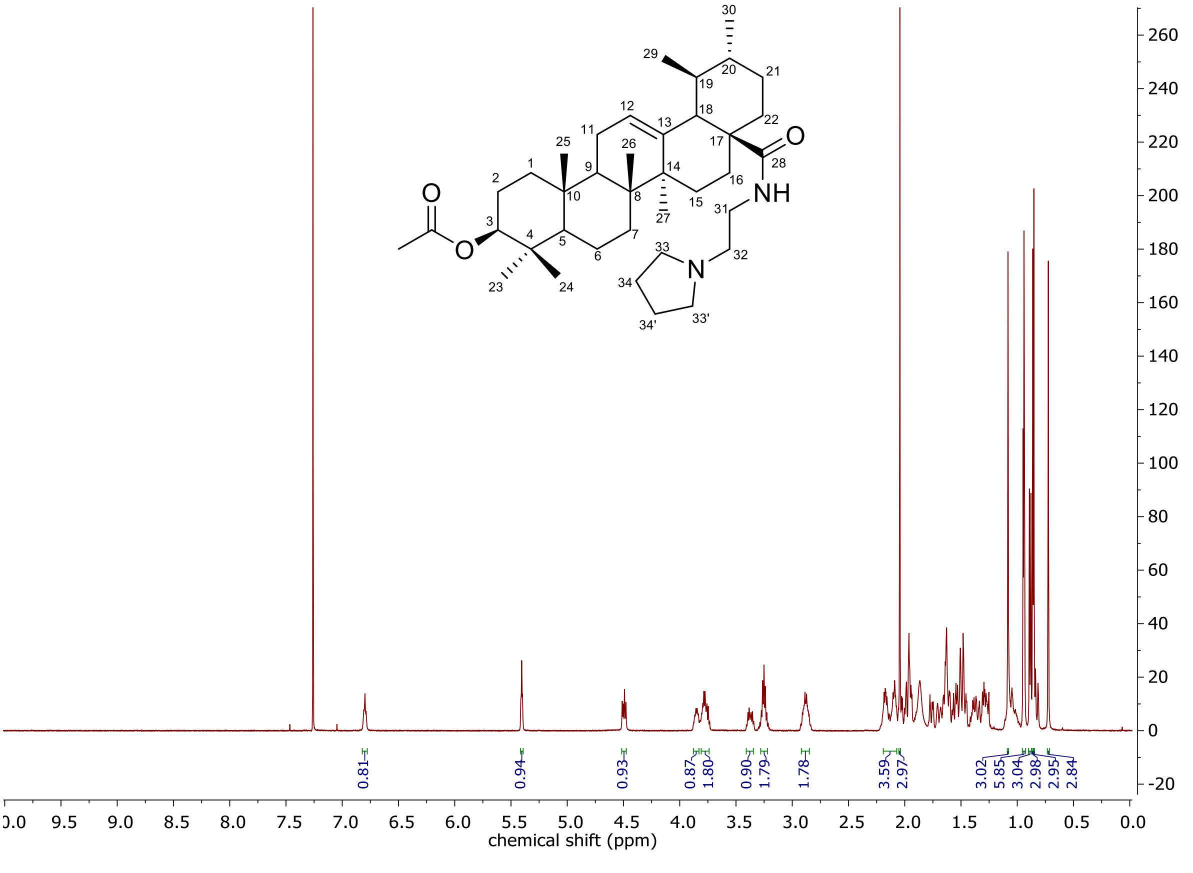

^{13}C NMR (APT)

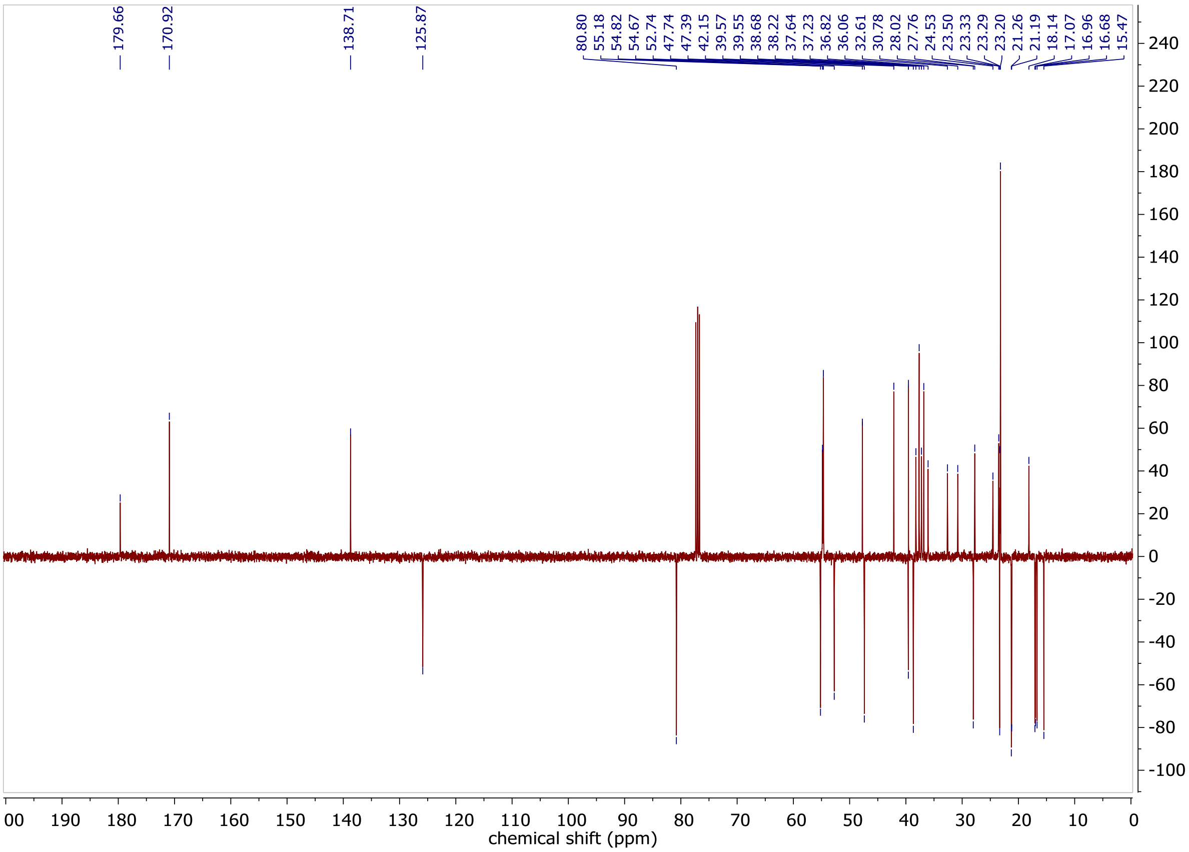

NMR spectra of 6

^{1}H NMR

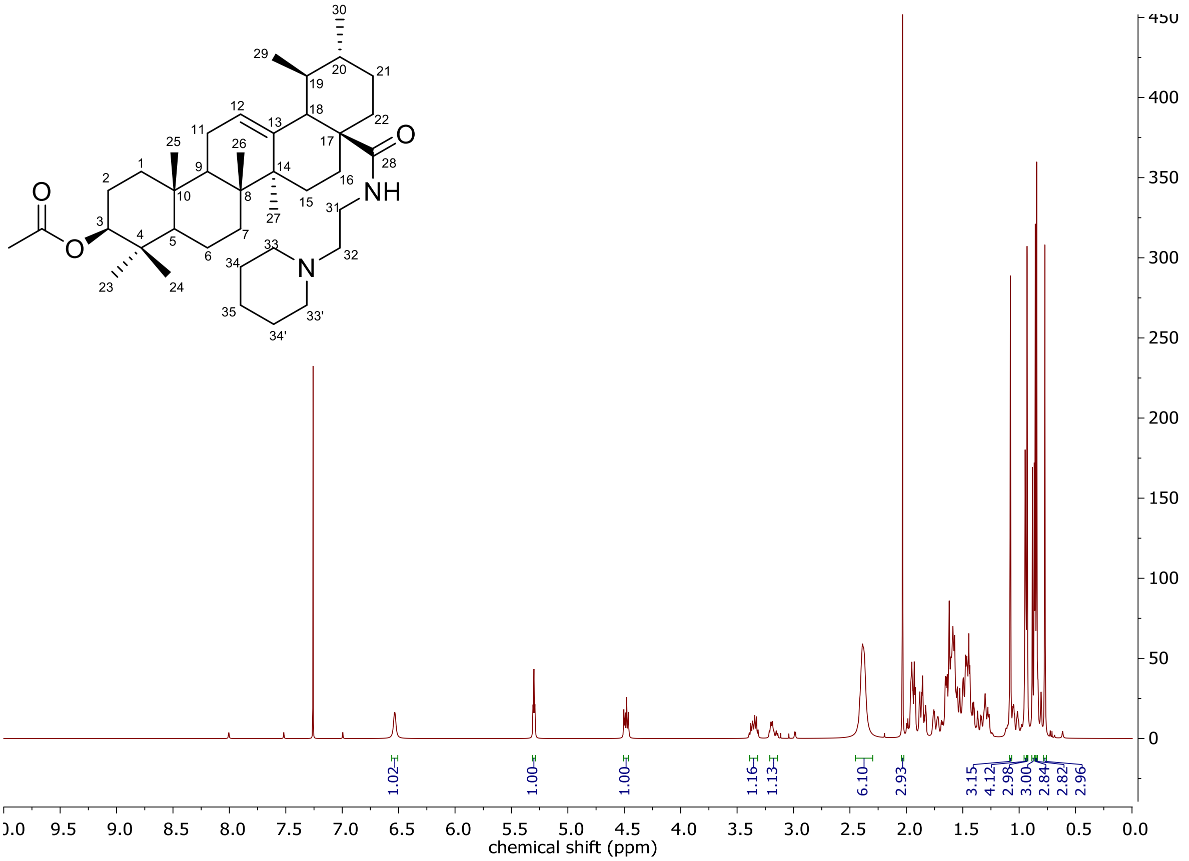

^{13}C NMR (APT)

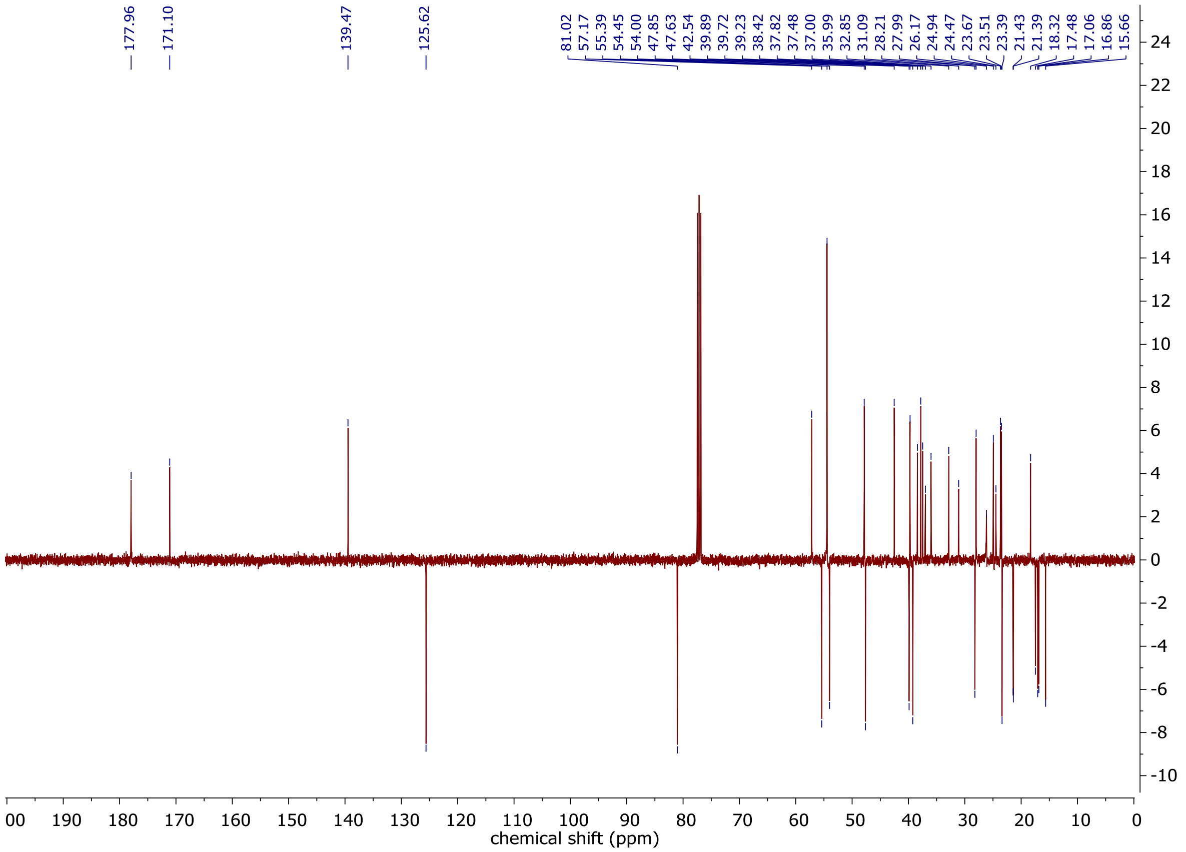

NMR spectra of 7

^{1}H NMR

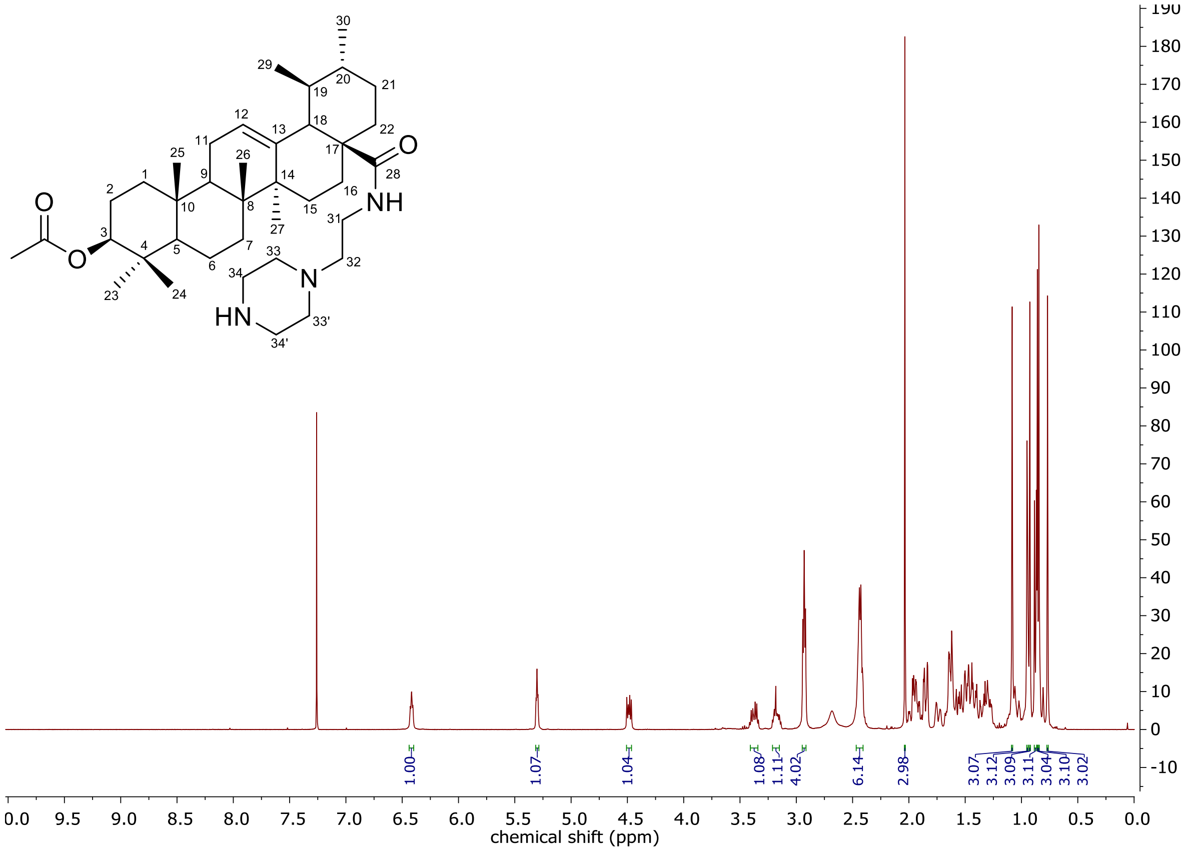

^{13}C NMR (APT)

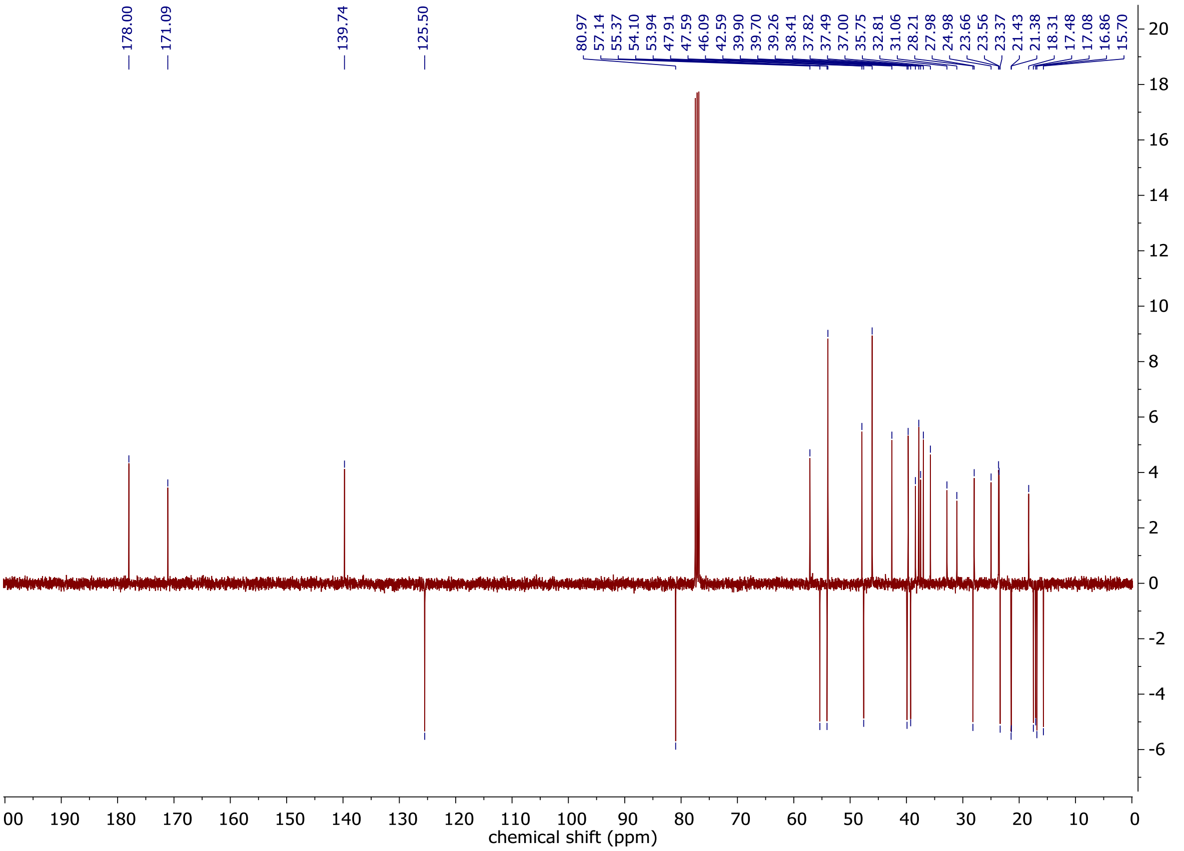

NMR spectra of 8

^{1}H NMR

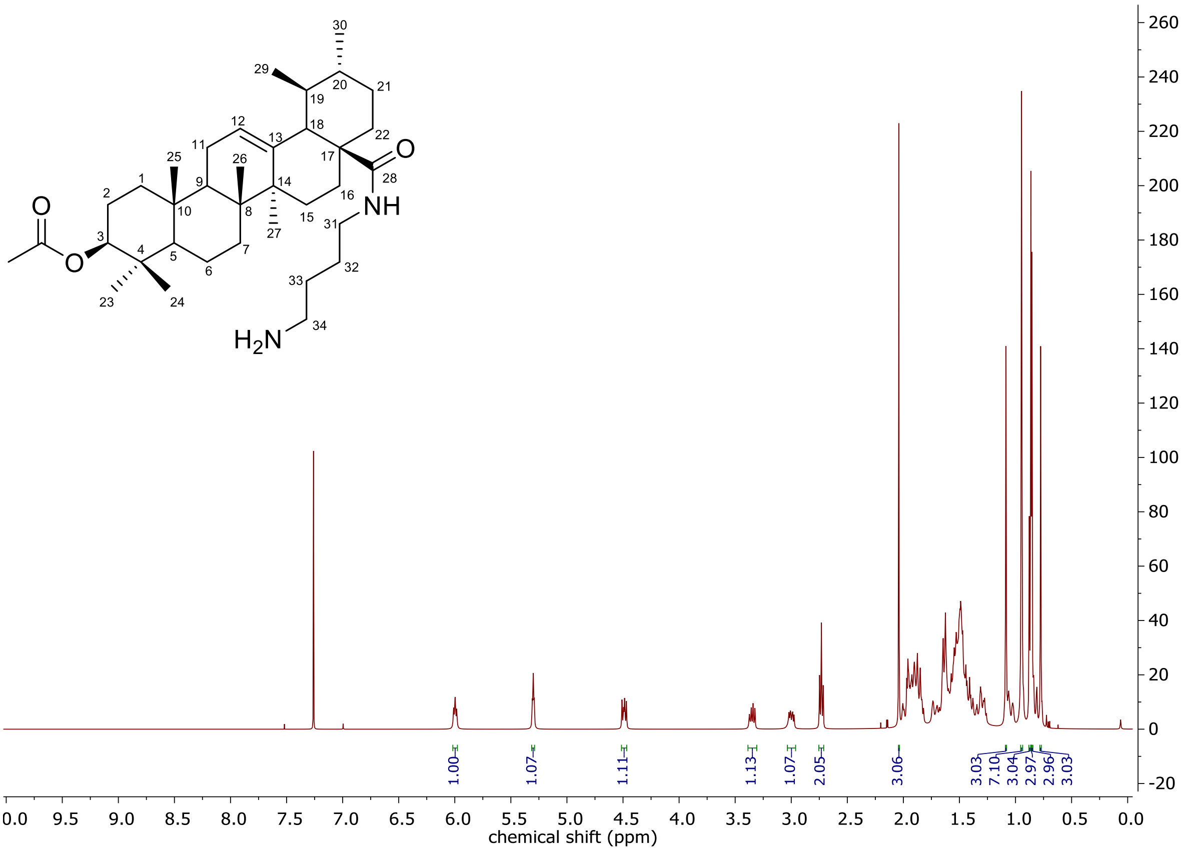

^{13}C NMR (APT)

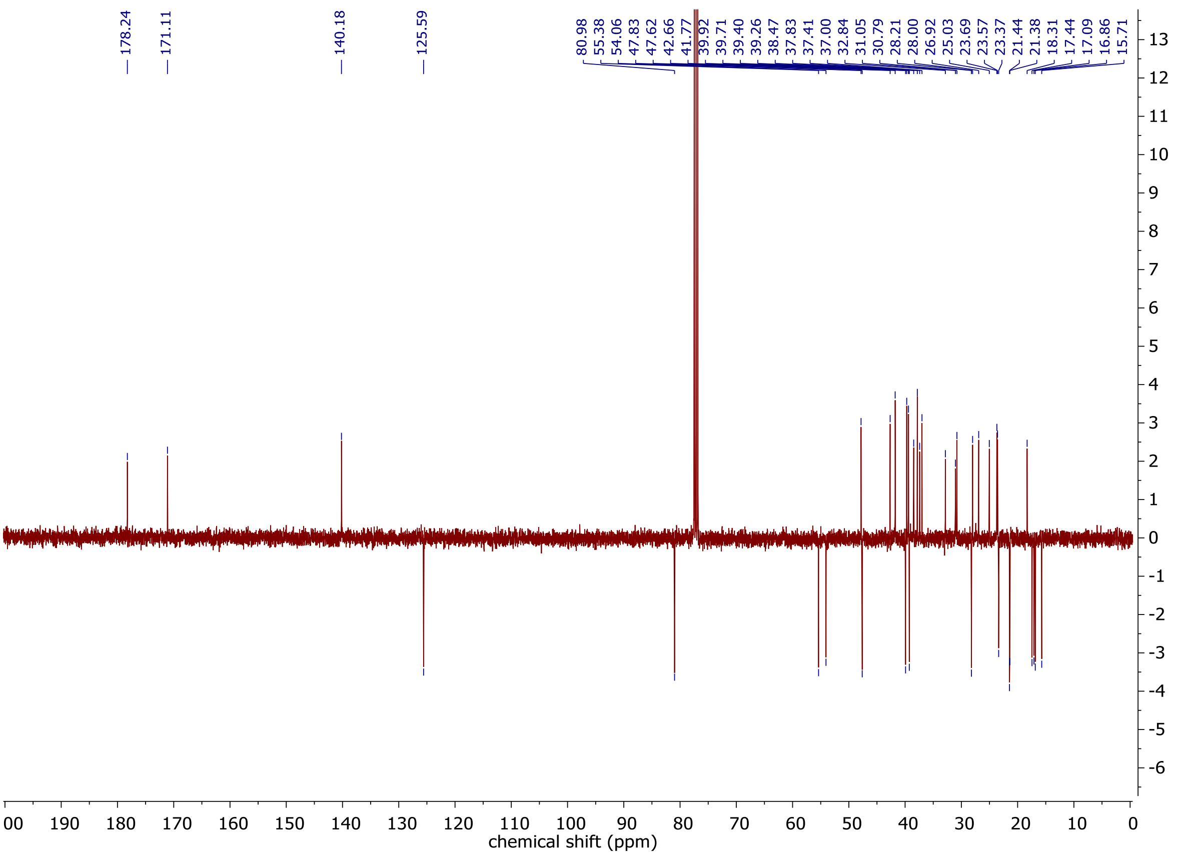

NMR spectra of 9

^{1}H NMR

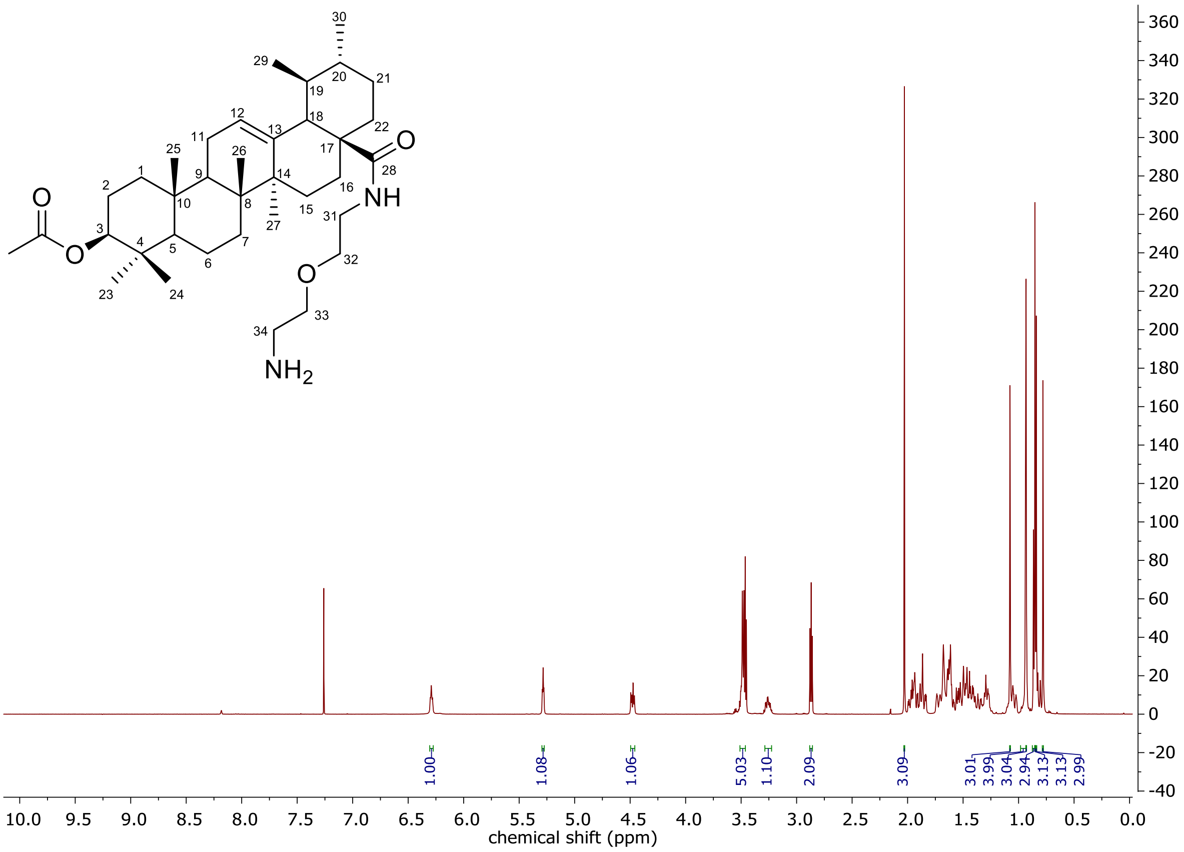

^{13}C NMR (APT)

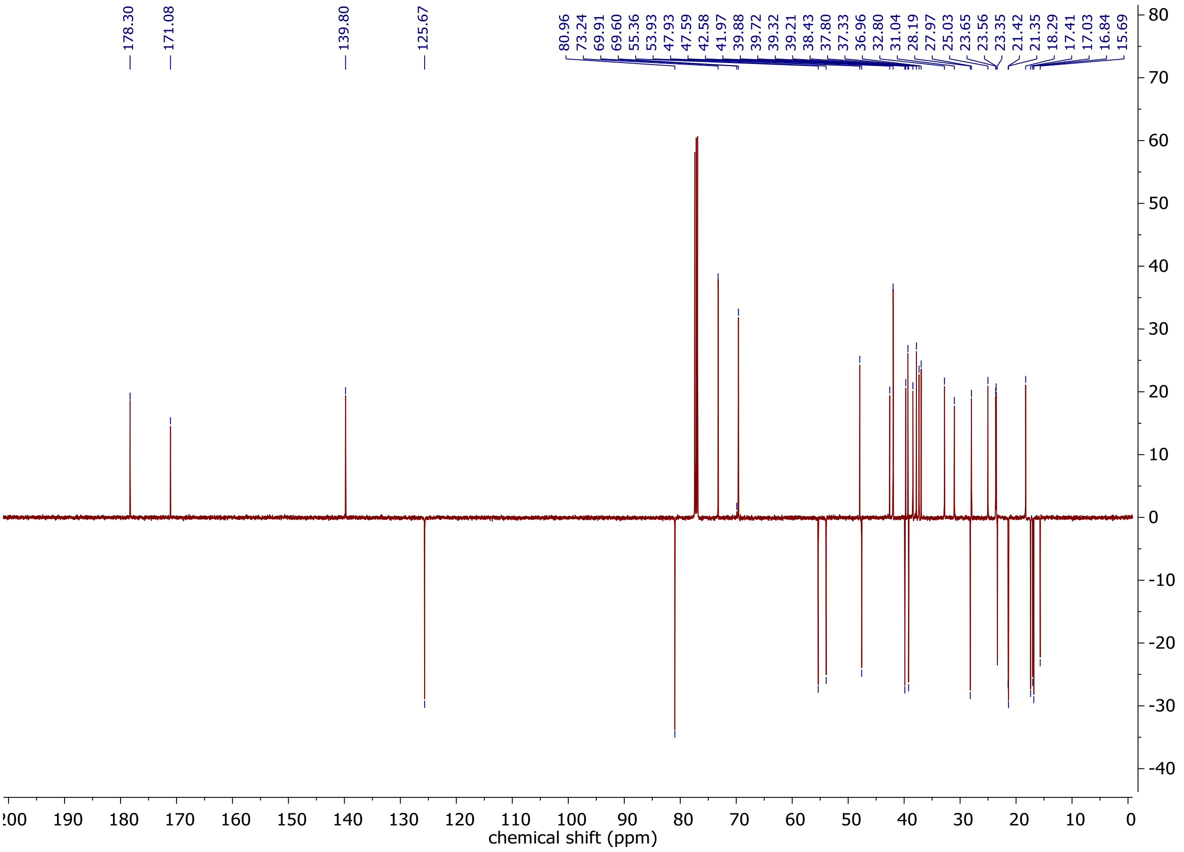

NMR spectra of 10

^{1}H NMR

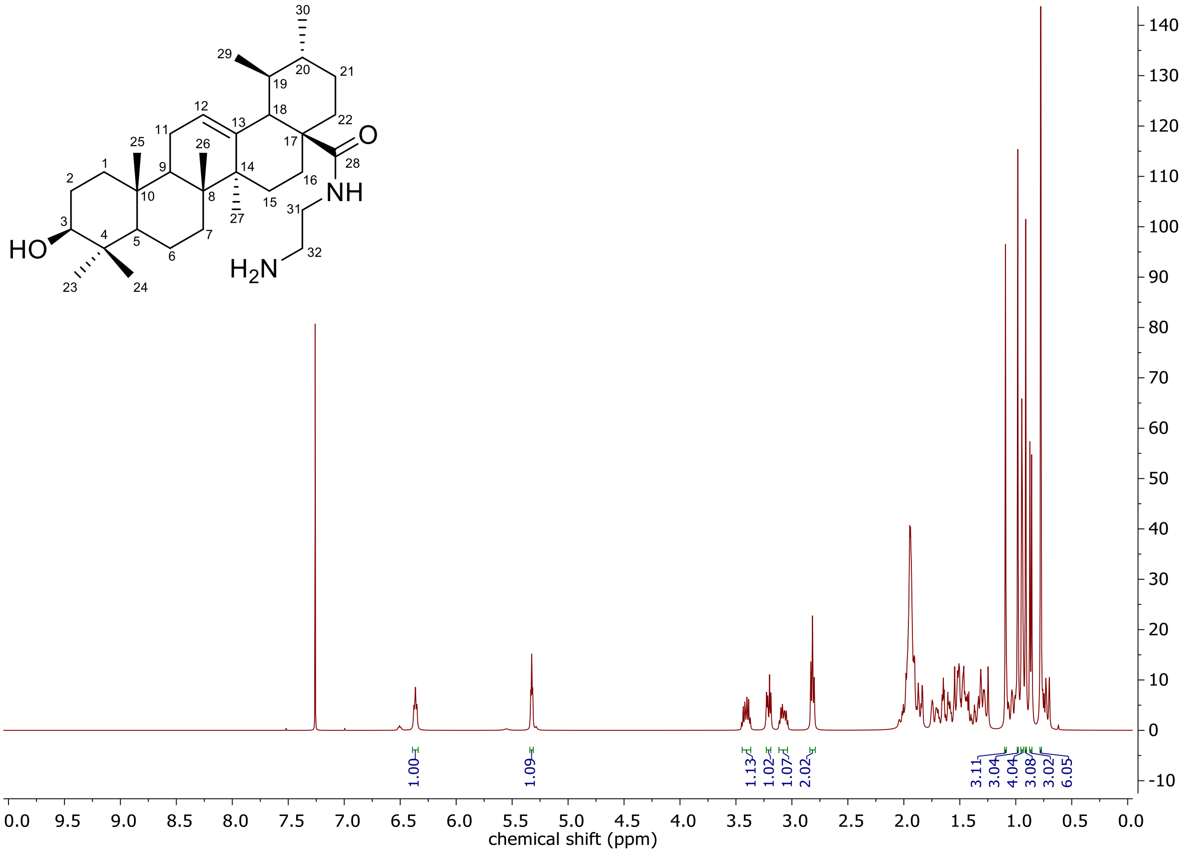

^{13}C NMR (APT)

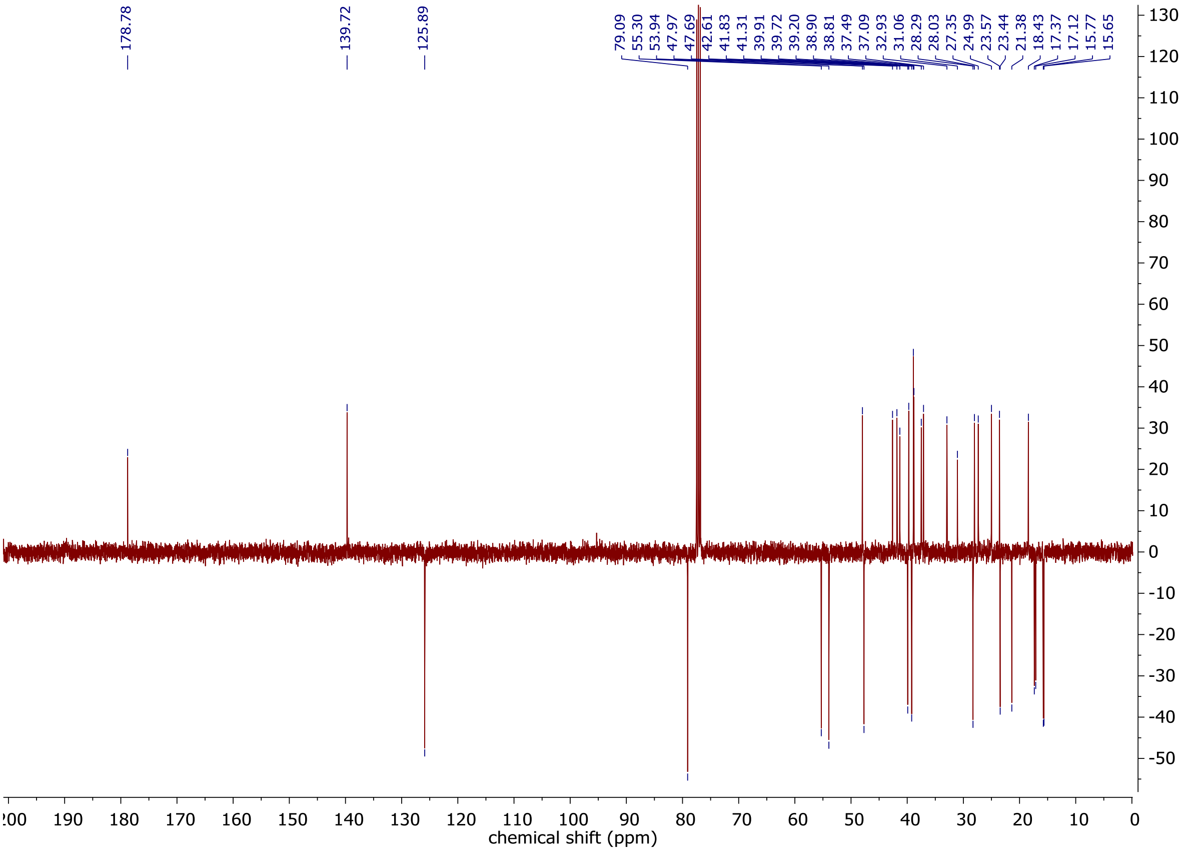

NMR spectra of 11

^{1}H NMR

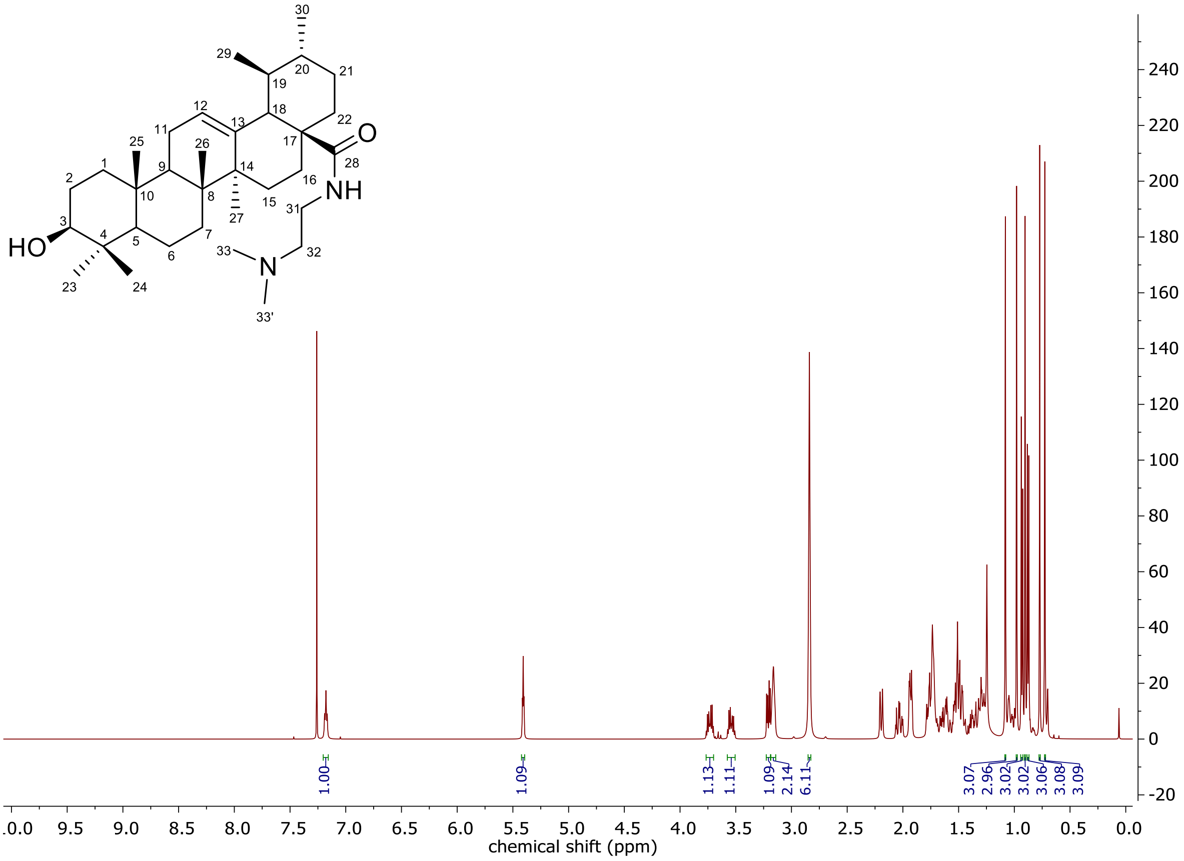

^{13}C NMR (APT)

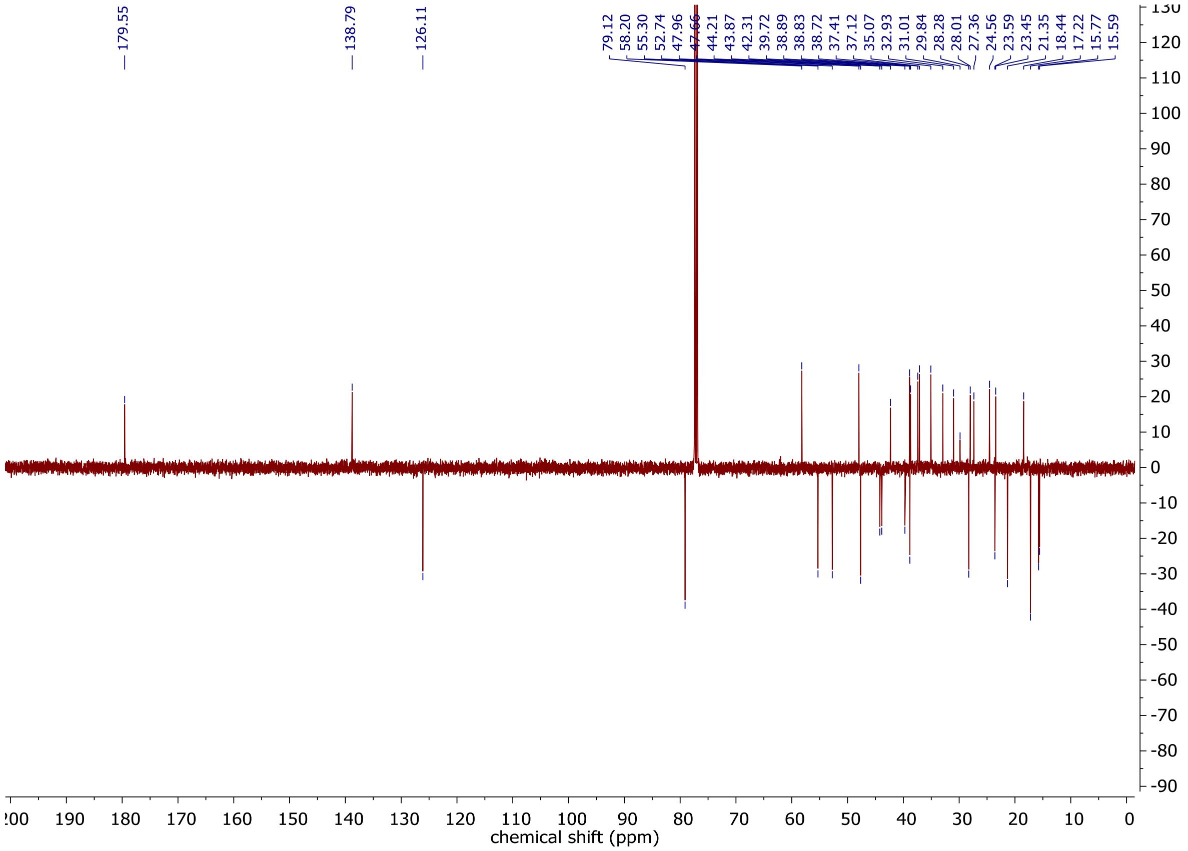

NMR spectra of 12

^{1}H NMR

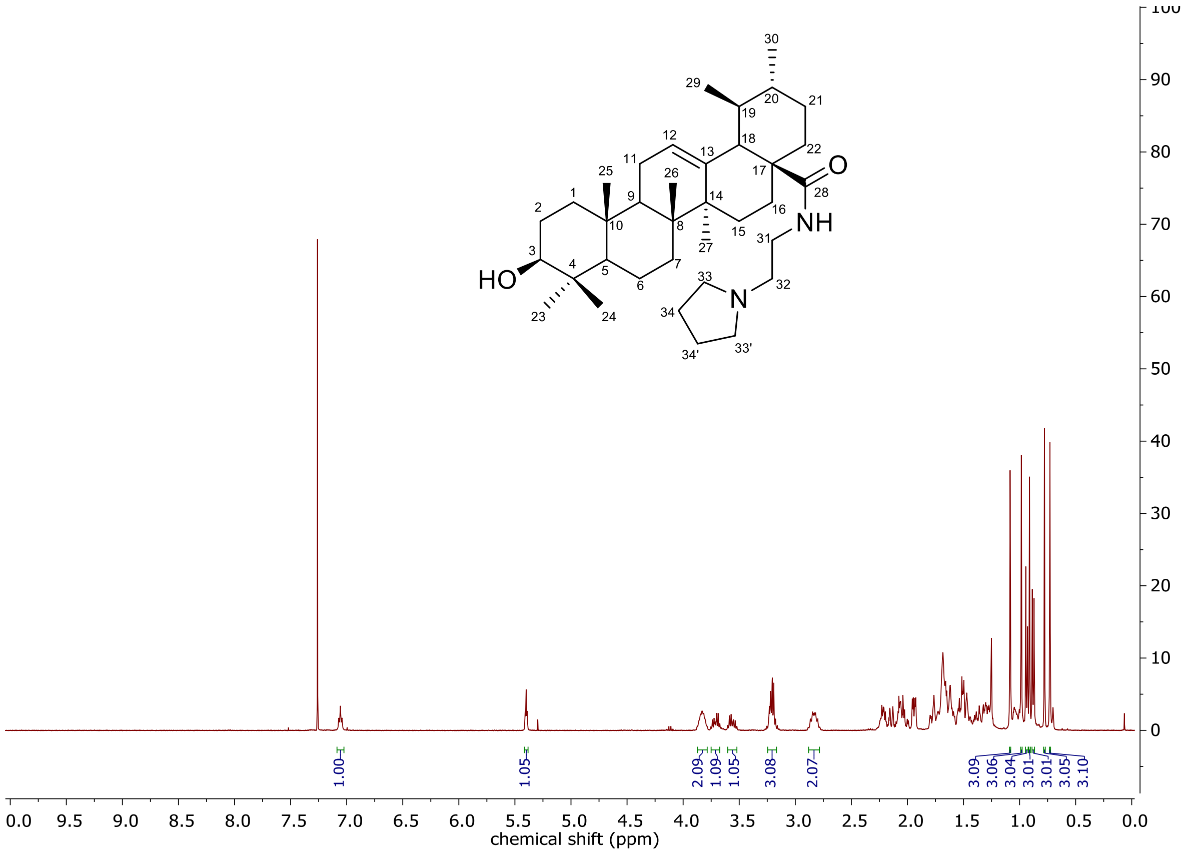

^{13}C NMR (APT)

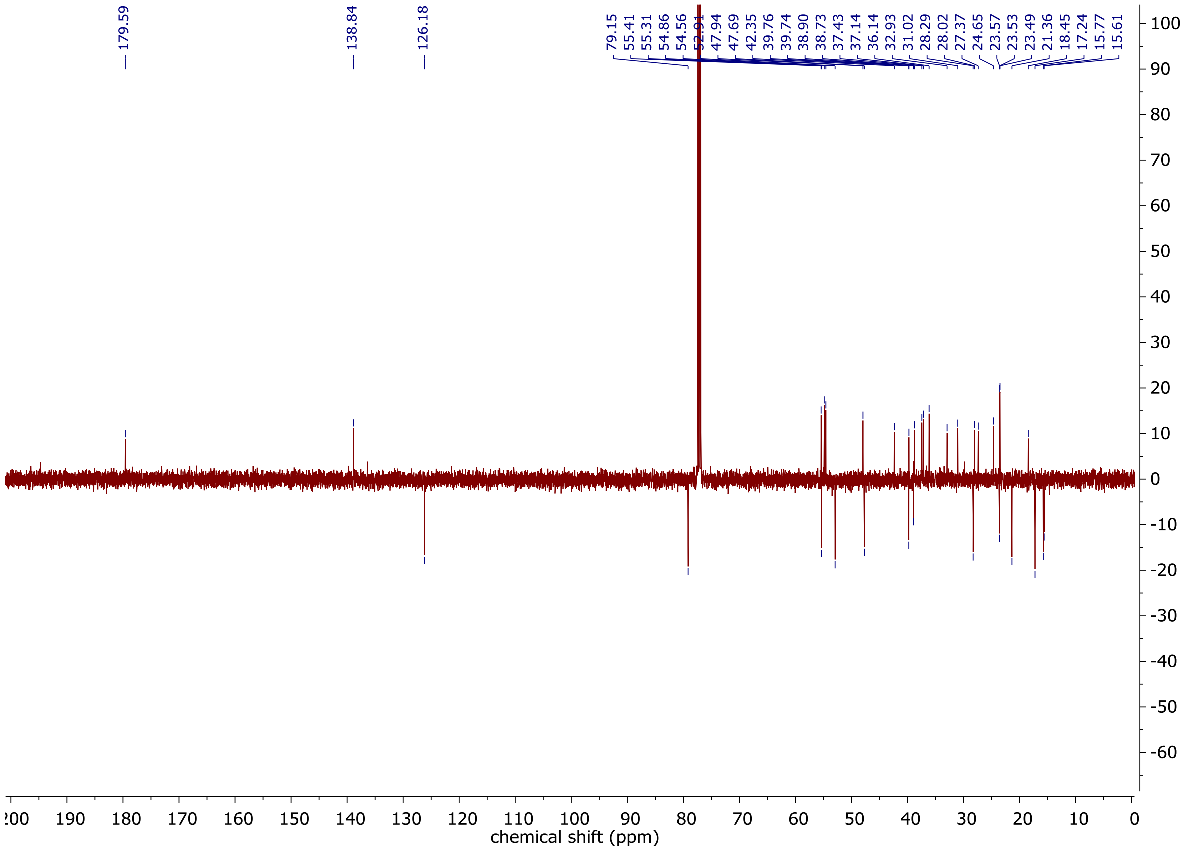

NMR spectra of 13

^{1}H NMR

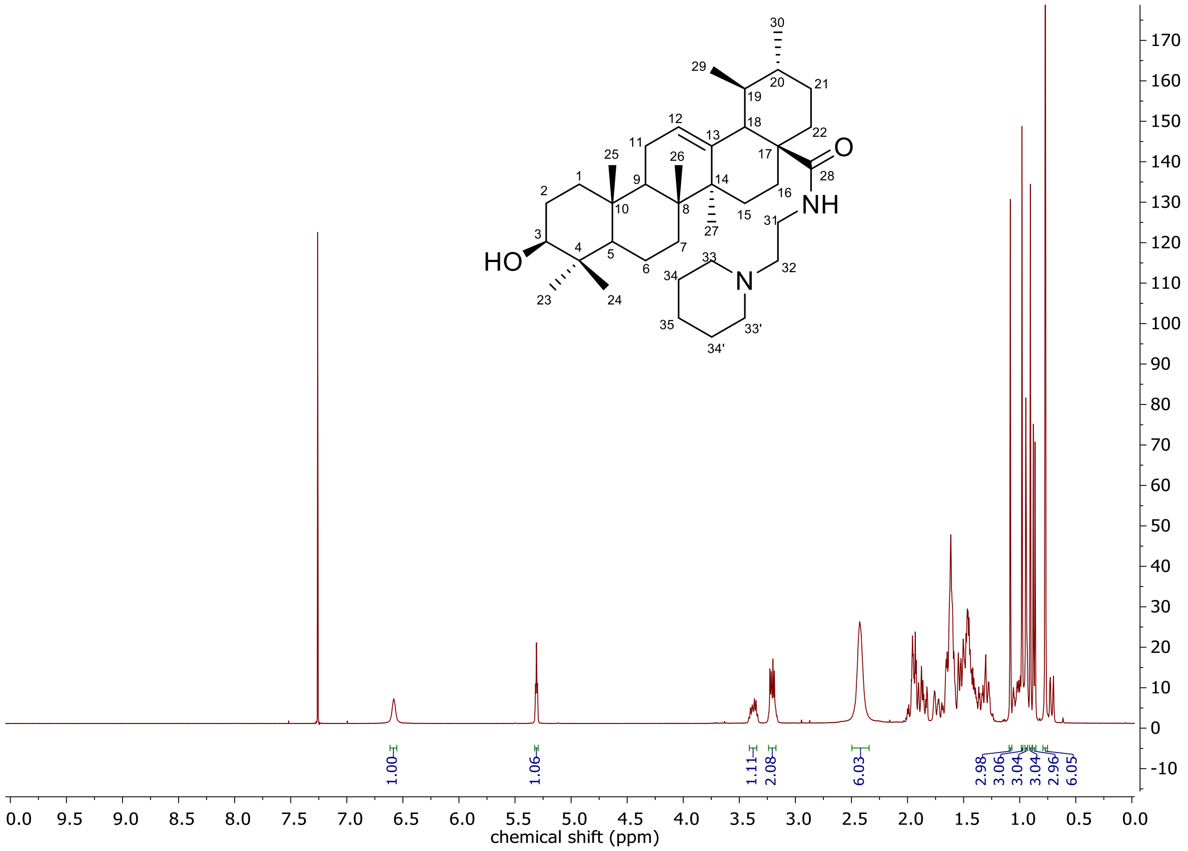

^{13}C NMR (APT)

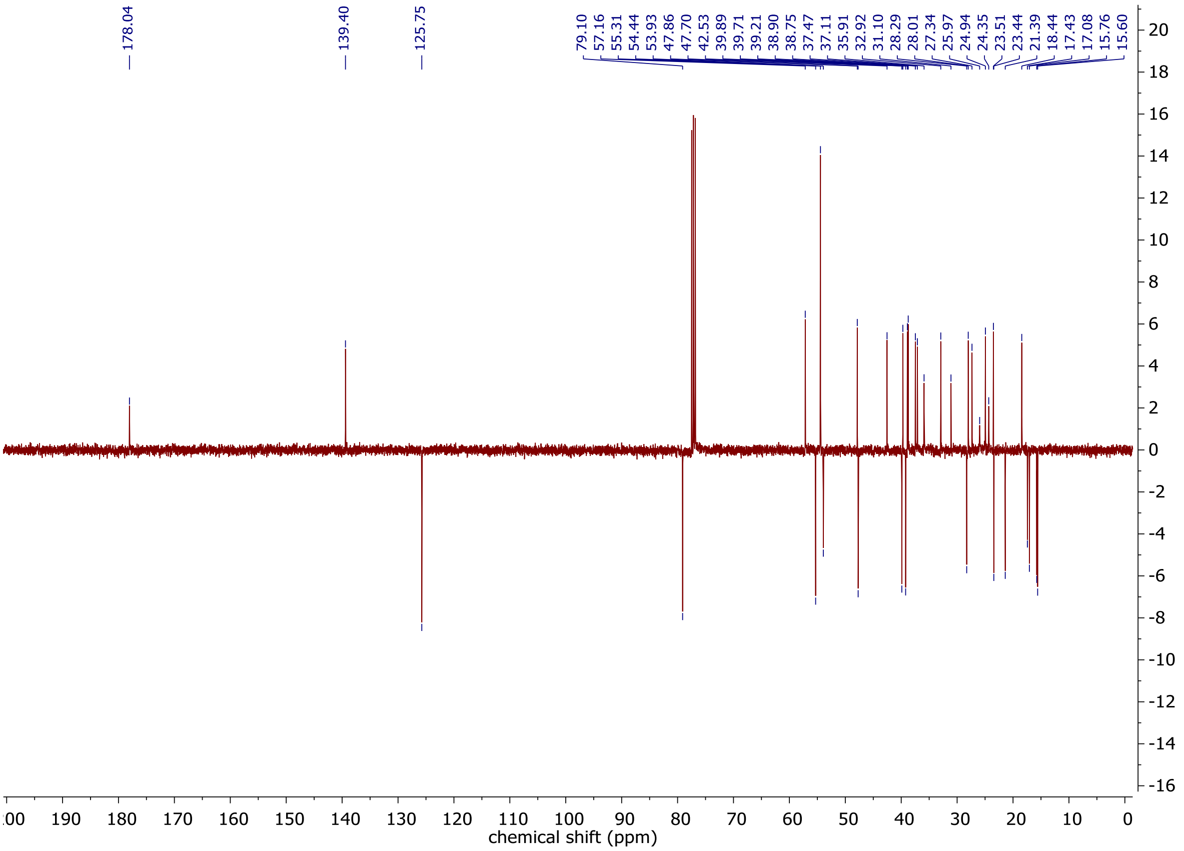

NMR spectra of 14

^{1}H NMR

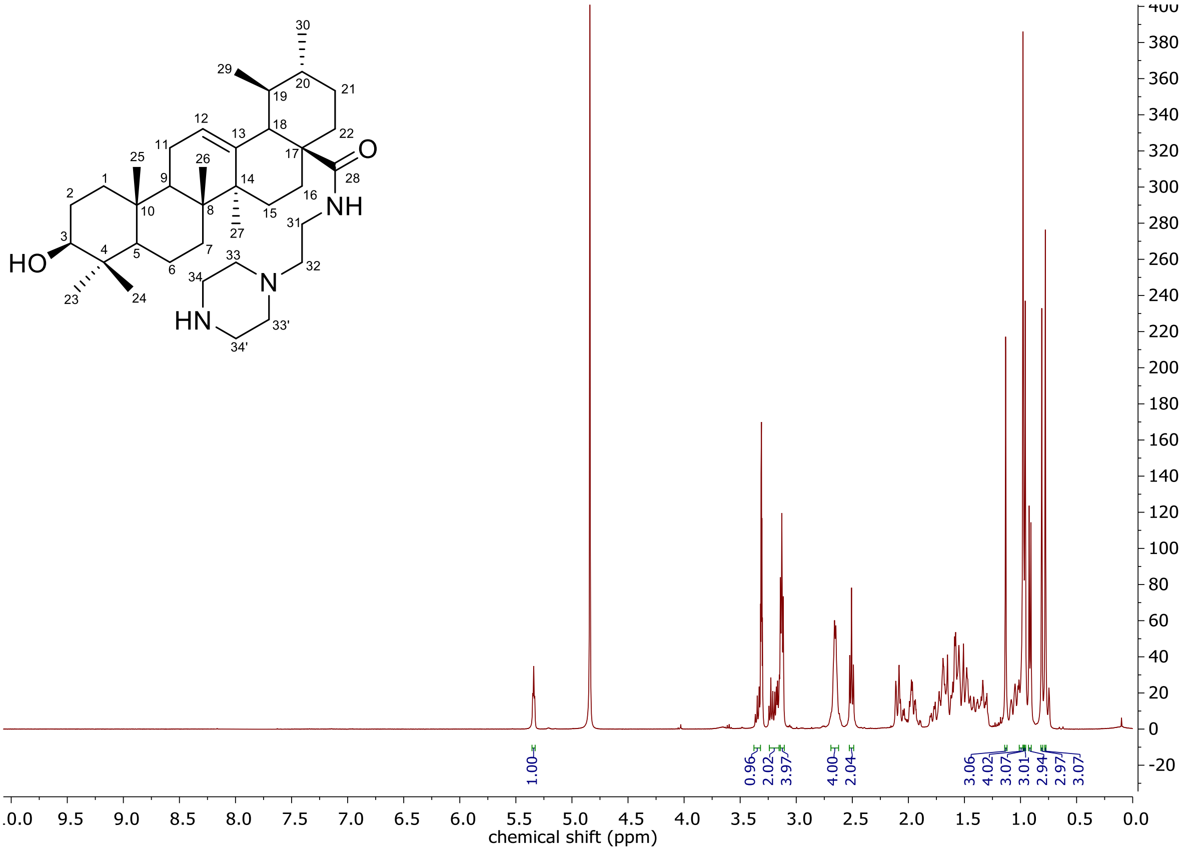

^{13}C NMR (APT)

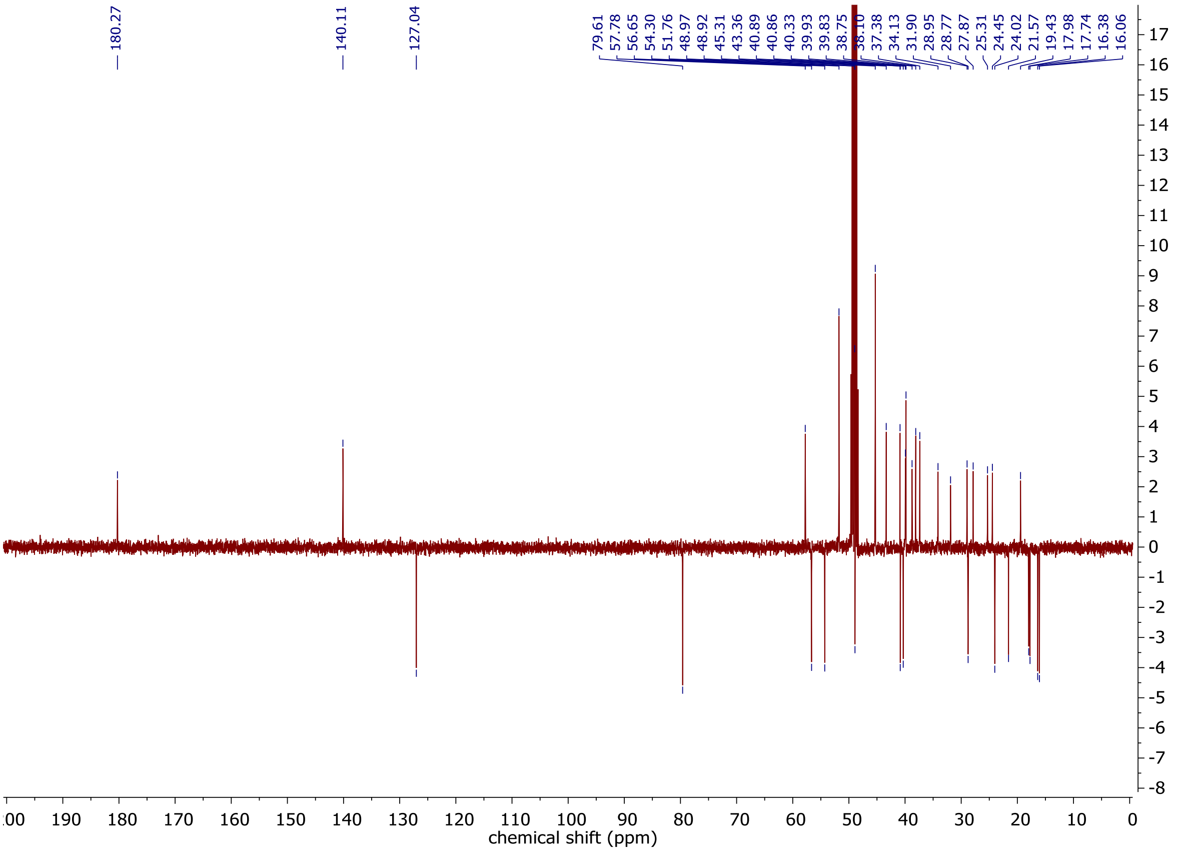

NMR spectra of 15

^{1}H NMR

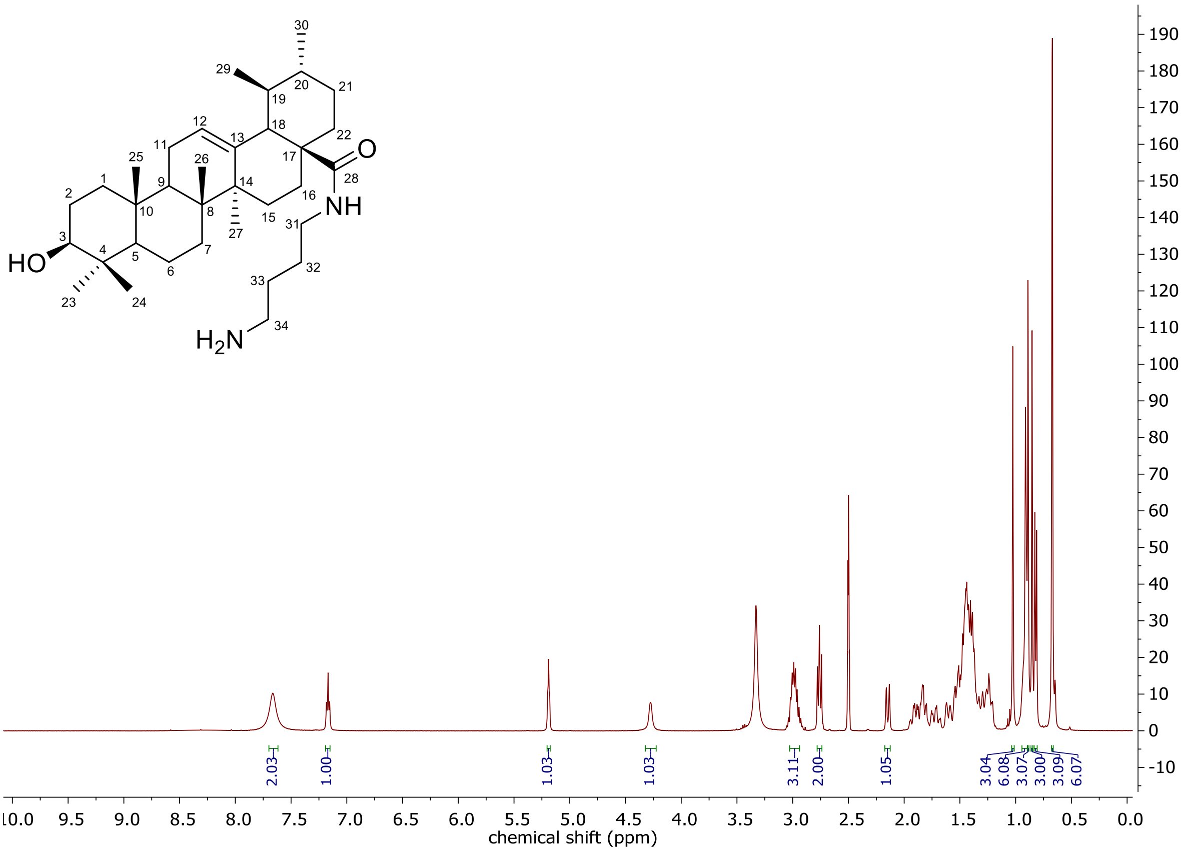

^{13}C NMR (APT)

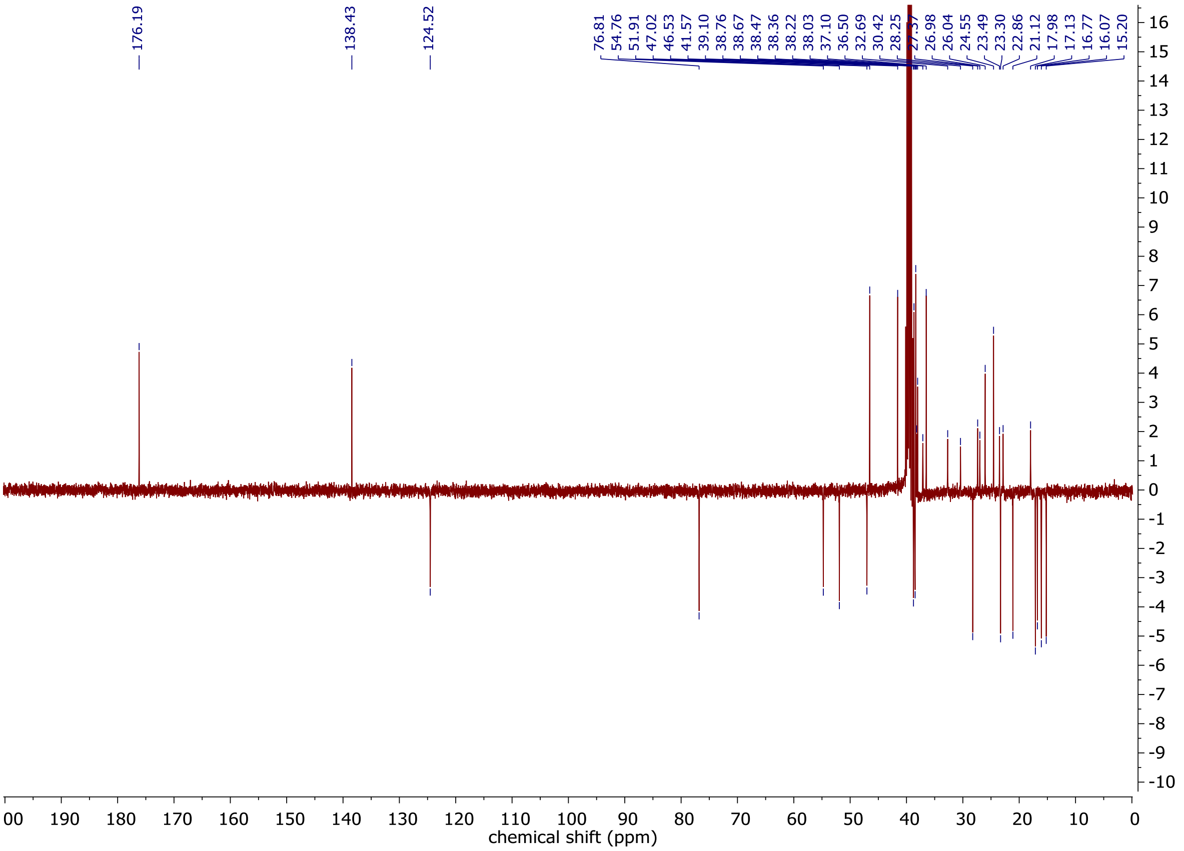

NMR spectra of 16

^{1}H NMR

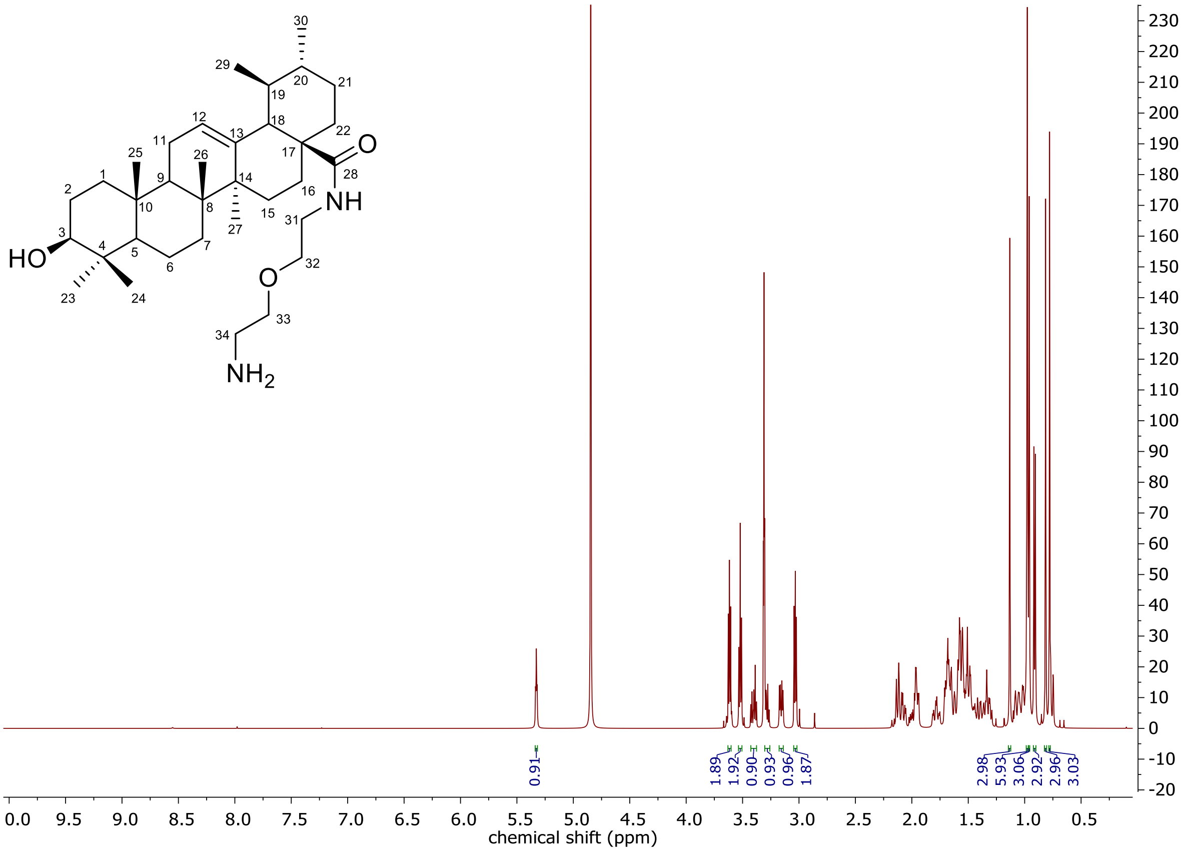

^{13}C NMR (APT)

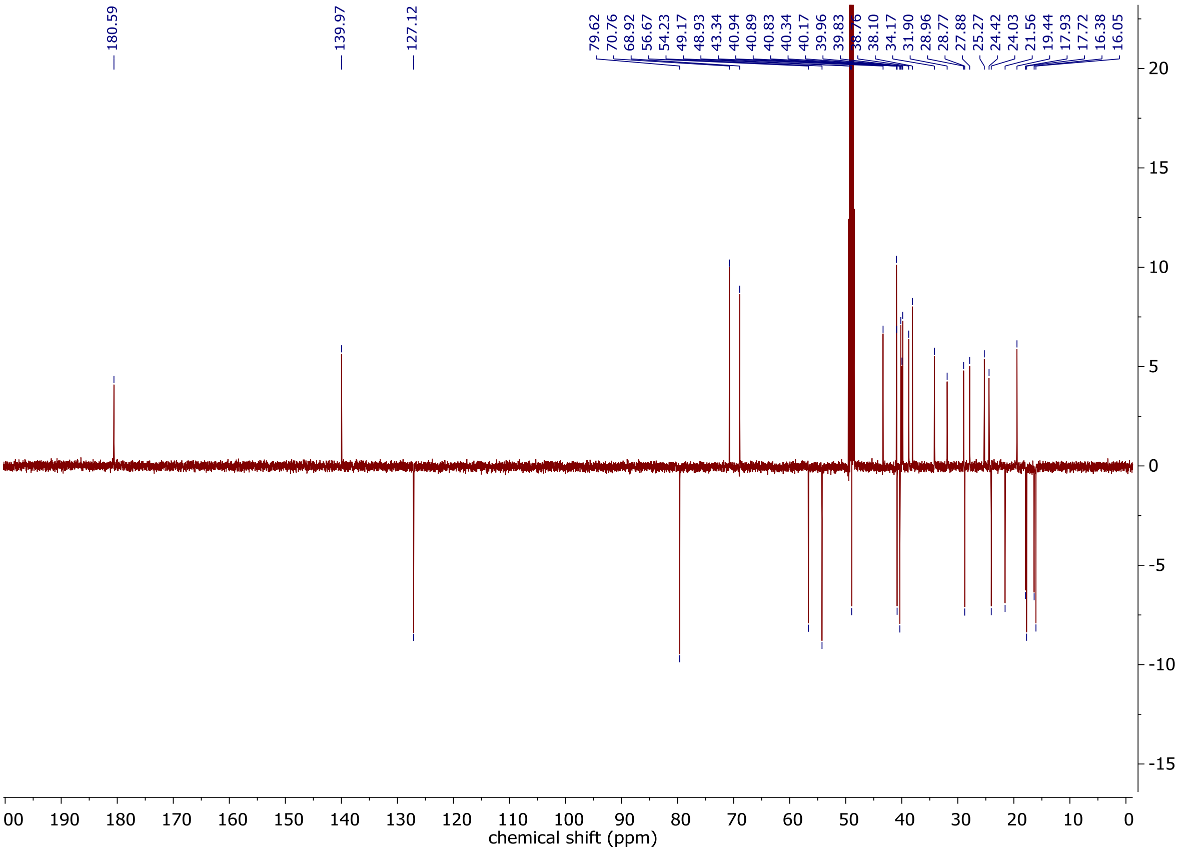

NMR spectra of 17

^{1}H NMR

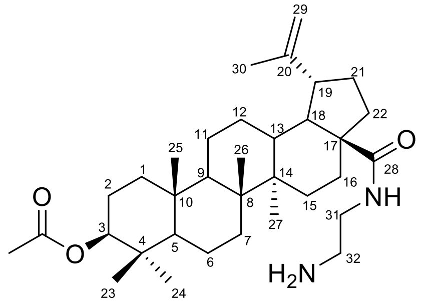

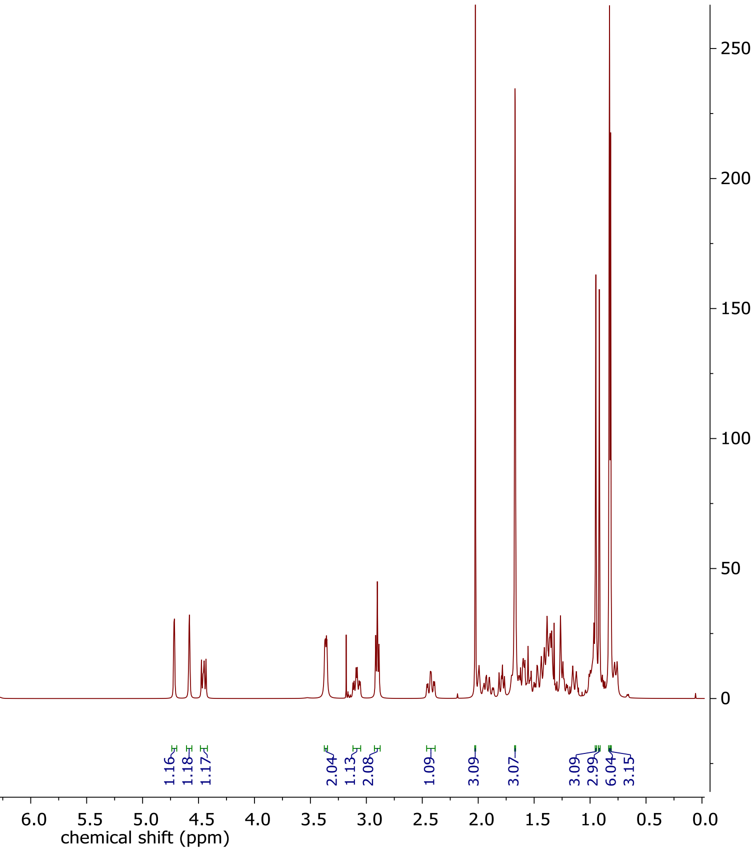

^{13}C NMR (APT)

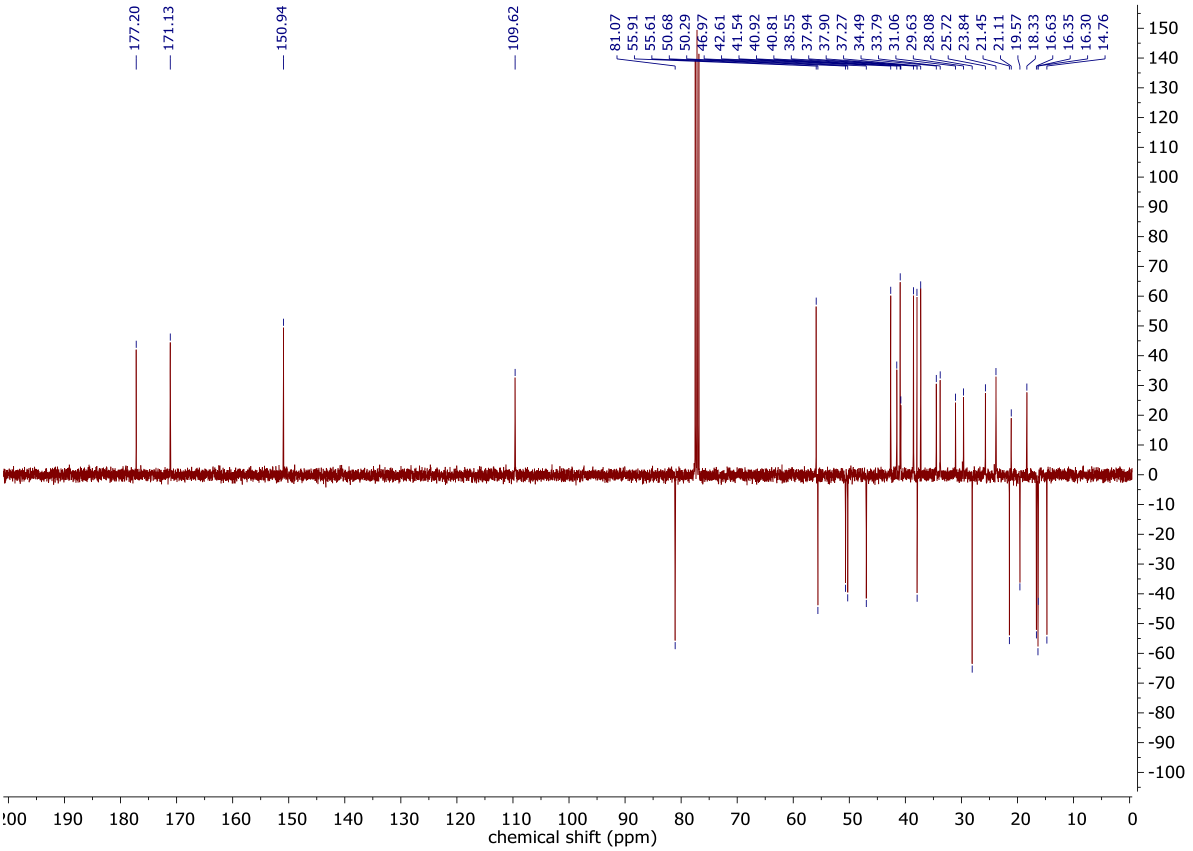

NMR spectra of 18

^{1}H NMR

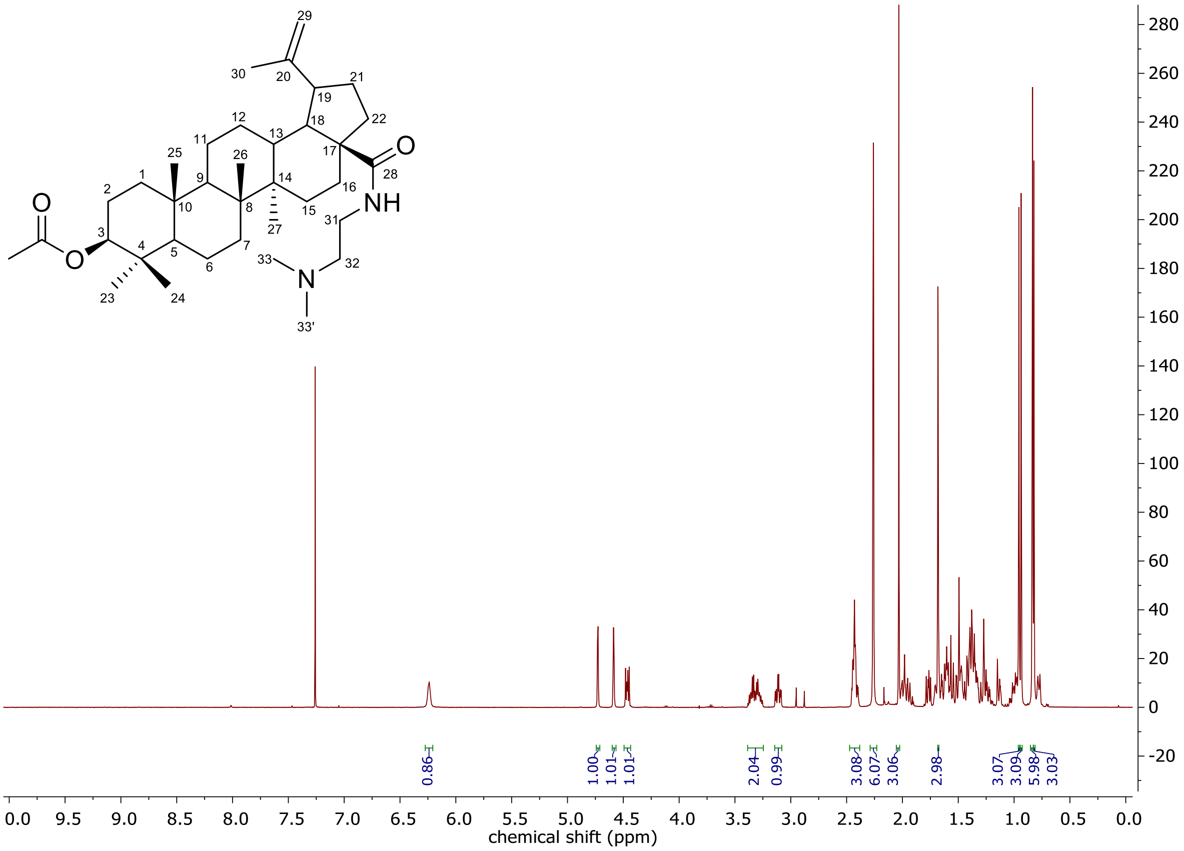

^{13}C NMR (APT)

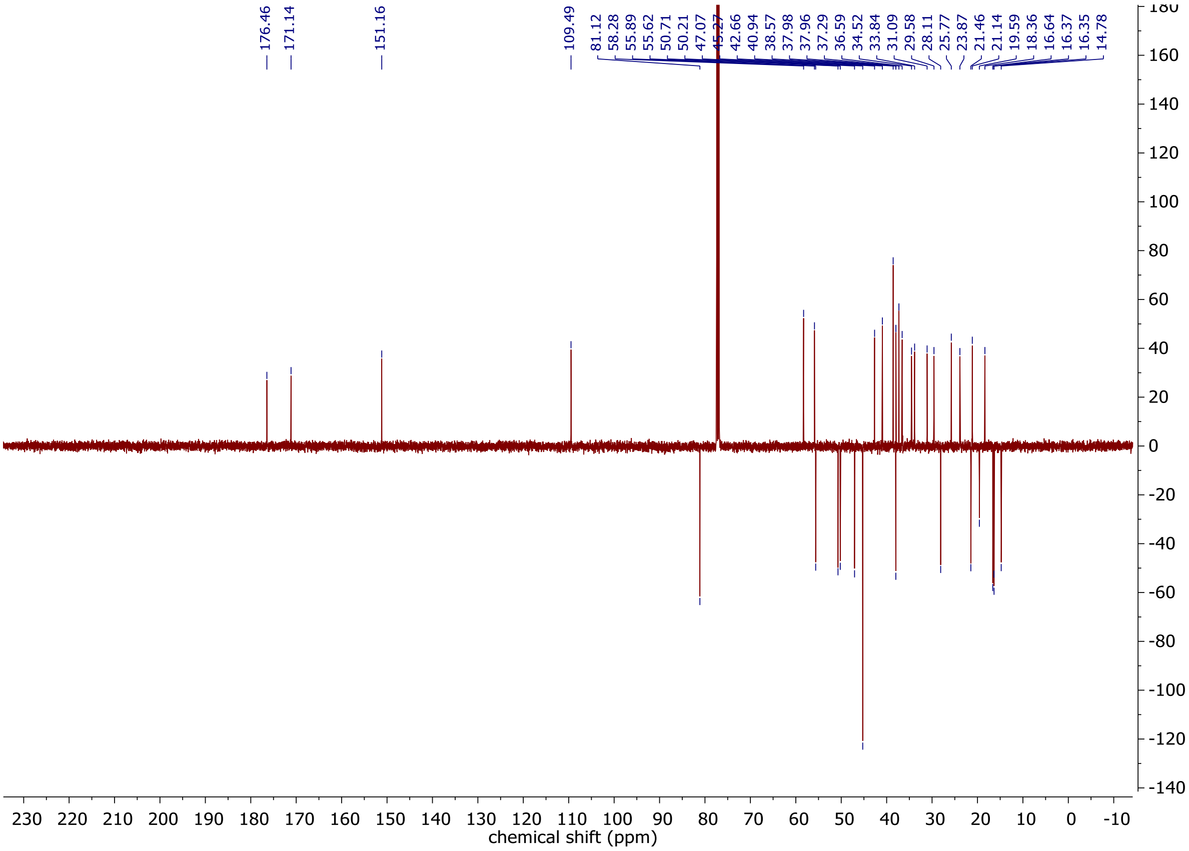

NMR spectra of 19

^{1}H NMR

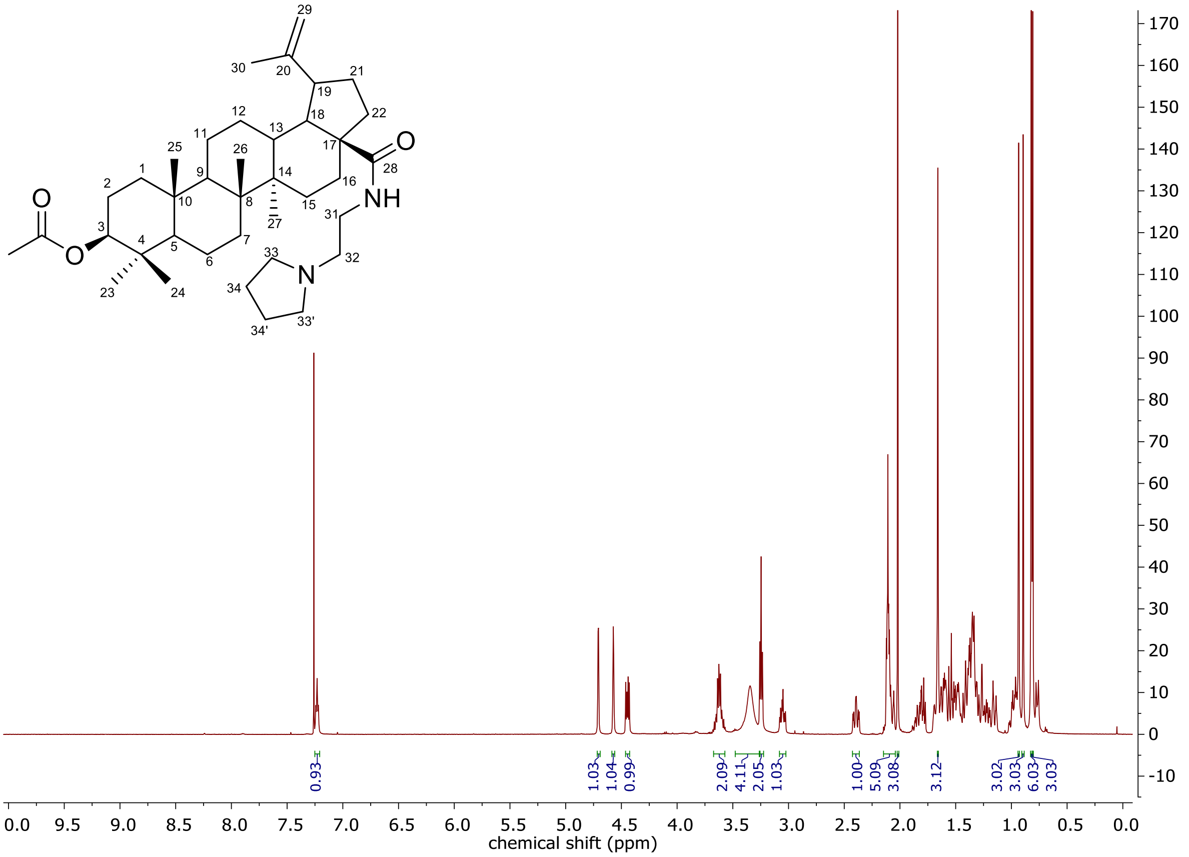

^{13}C NMR (APT)

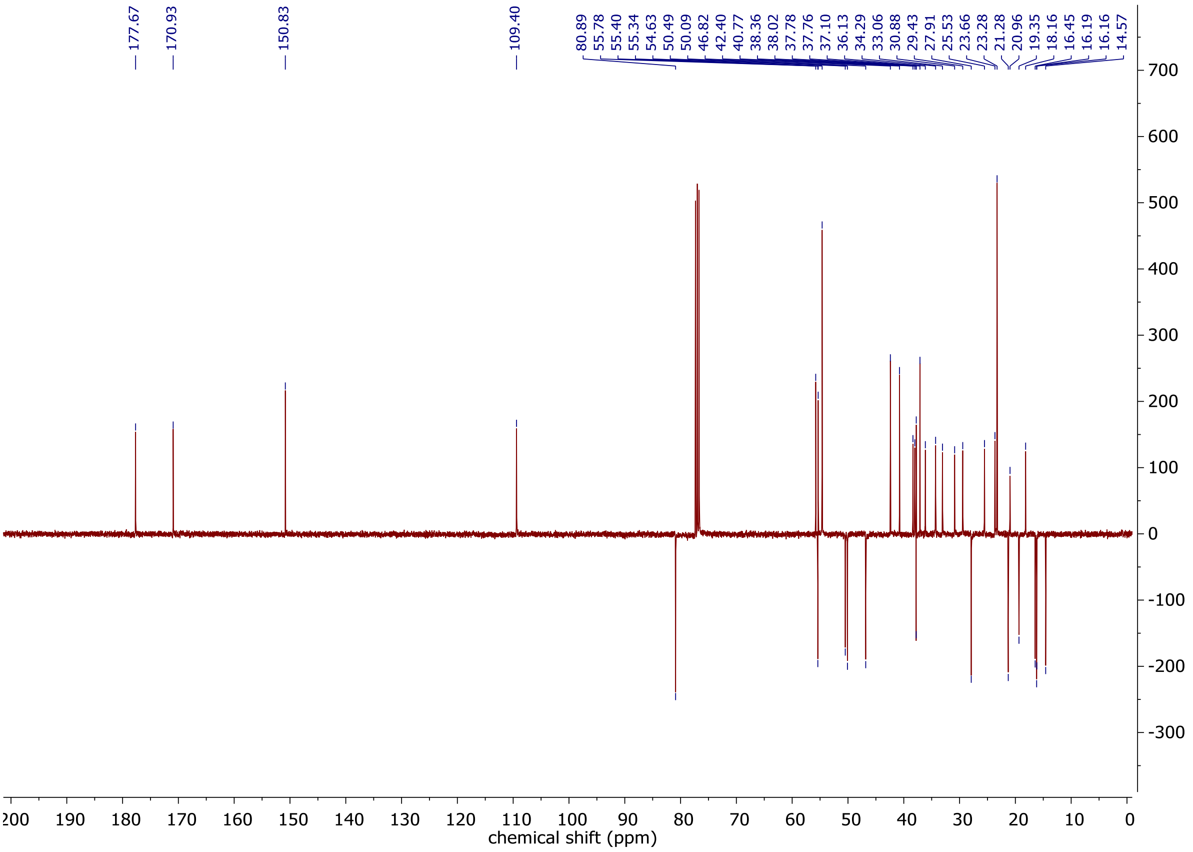

NMR spectra of 20

^{1}H NMR

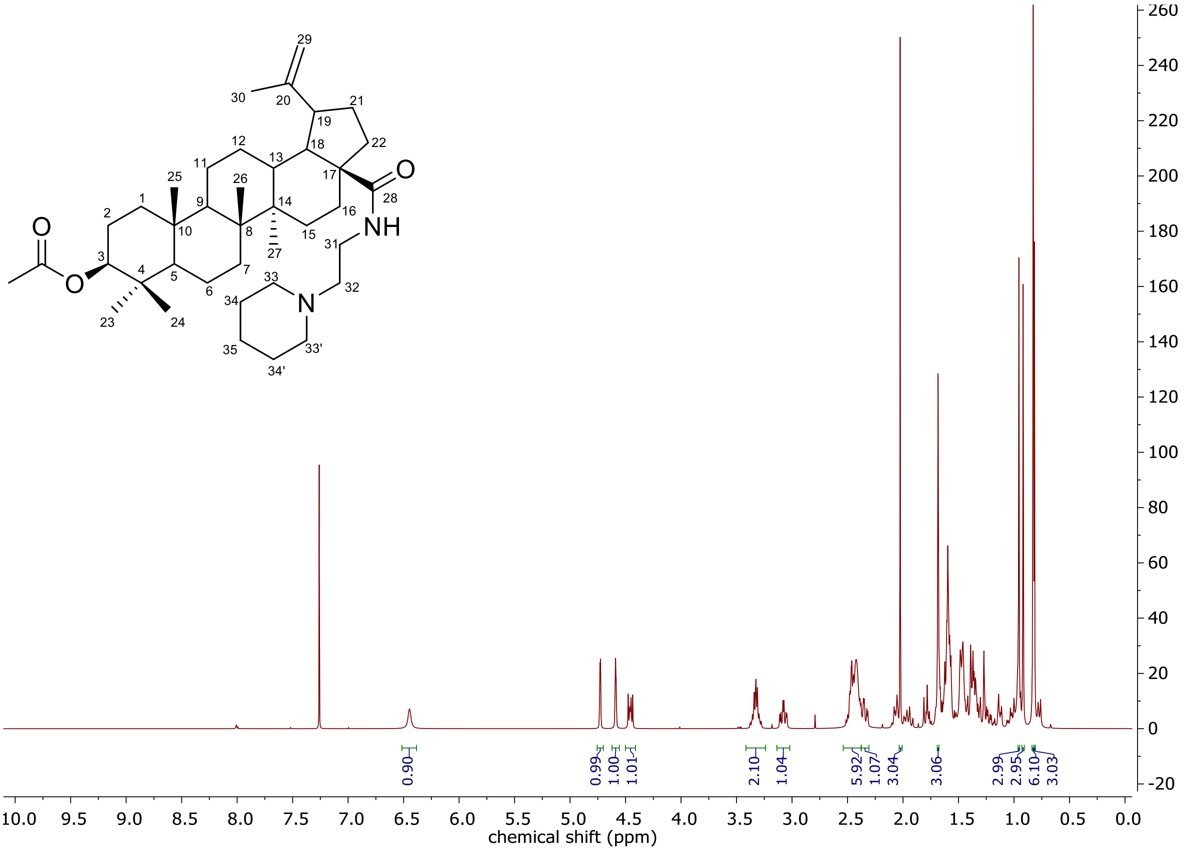

^{13}C NMR (APT)

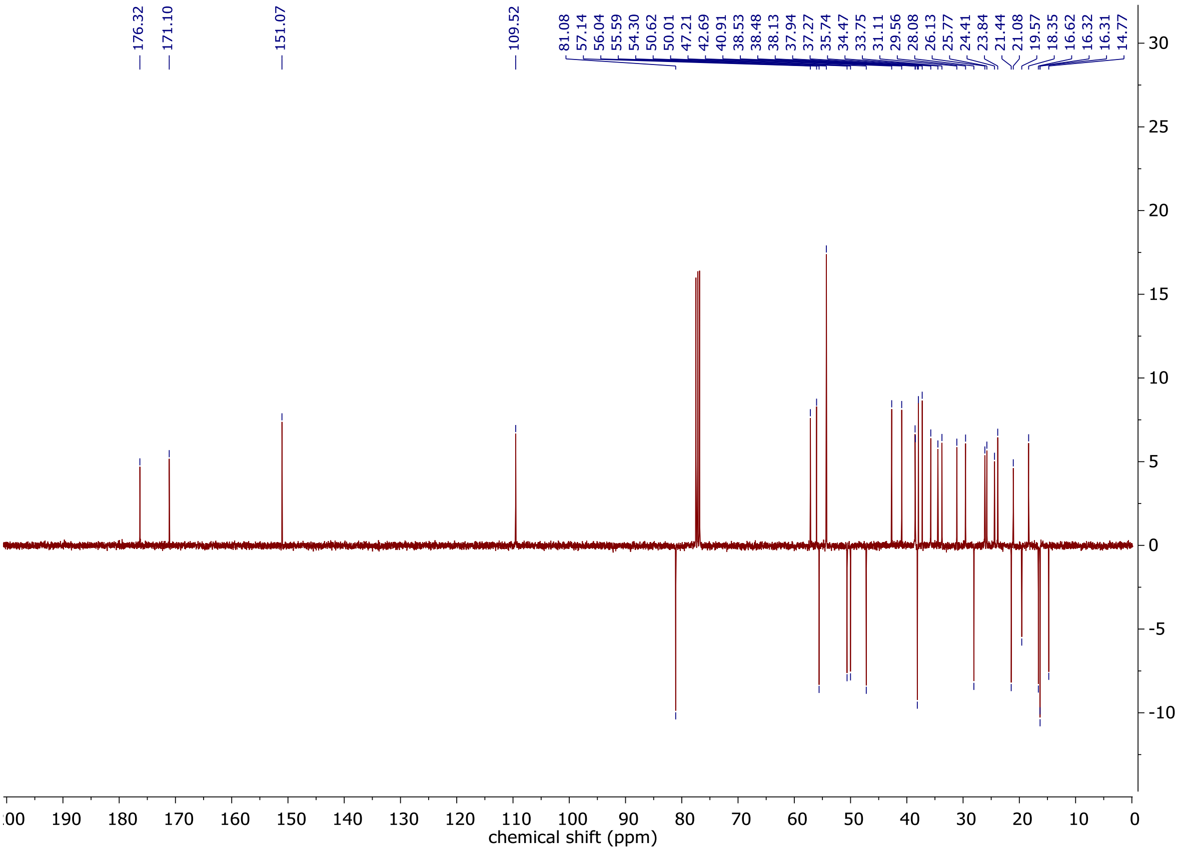

NMR spectra of 21

^{1}H NMR

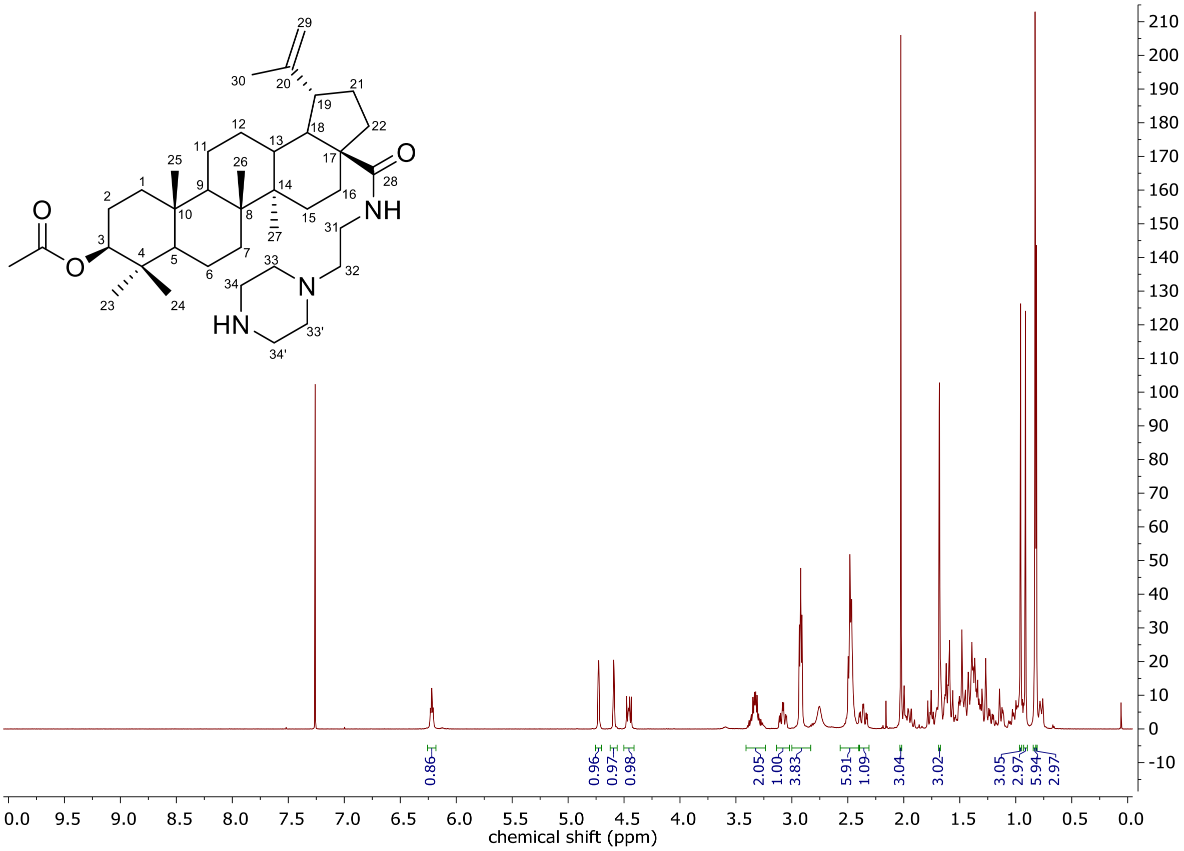

^{13}C NMR (APT)

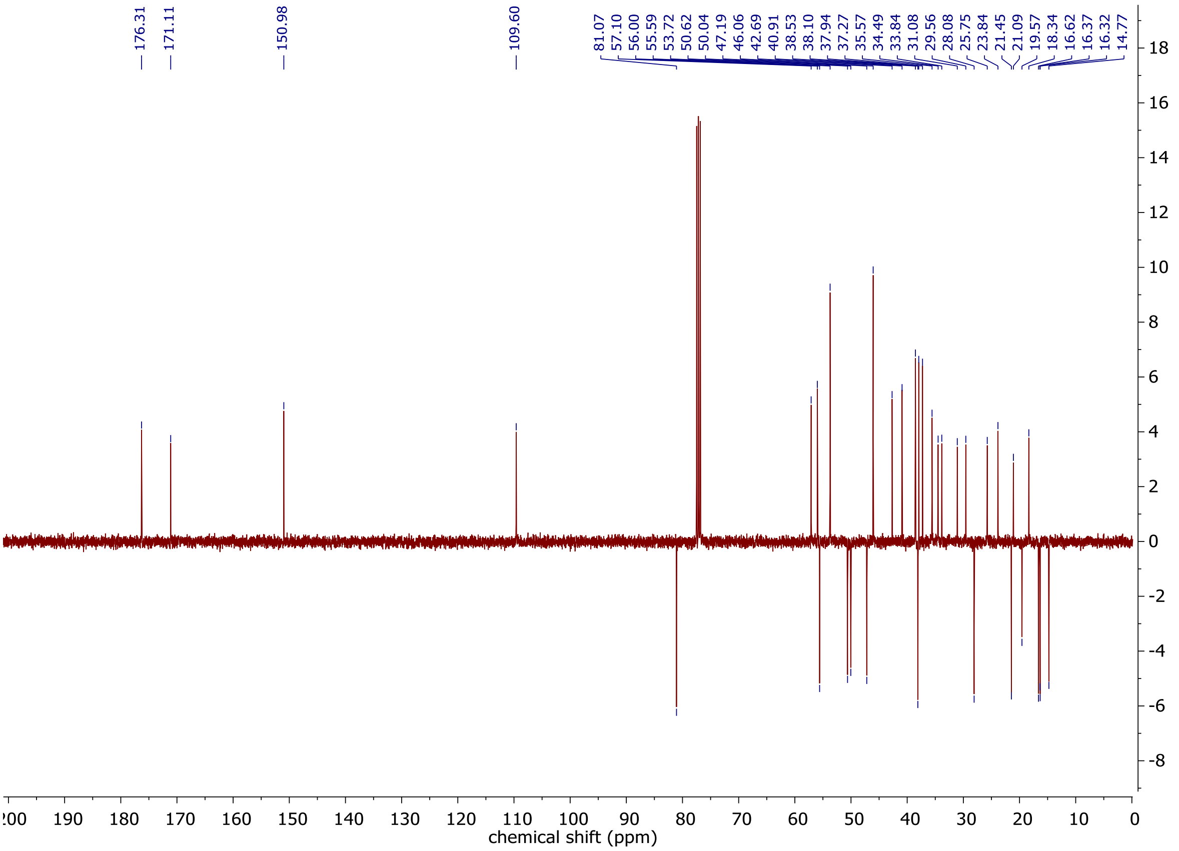

NMR spectra of 22

^{1}H NMR

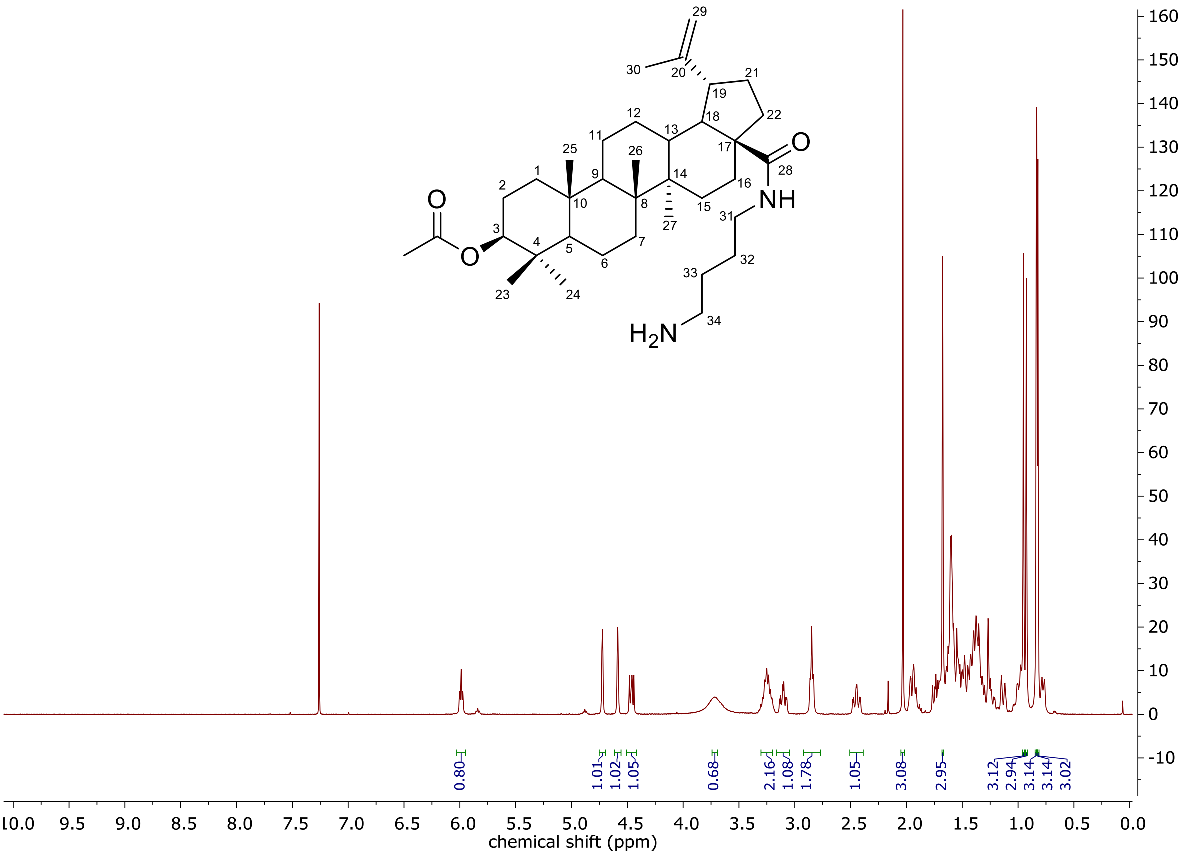

^{13}C NMR (APT)

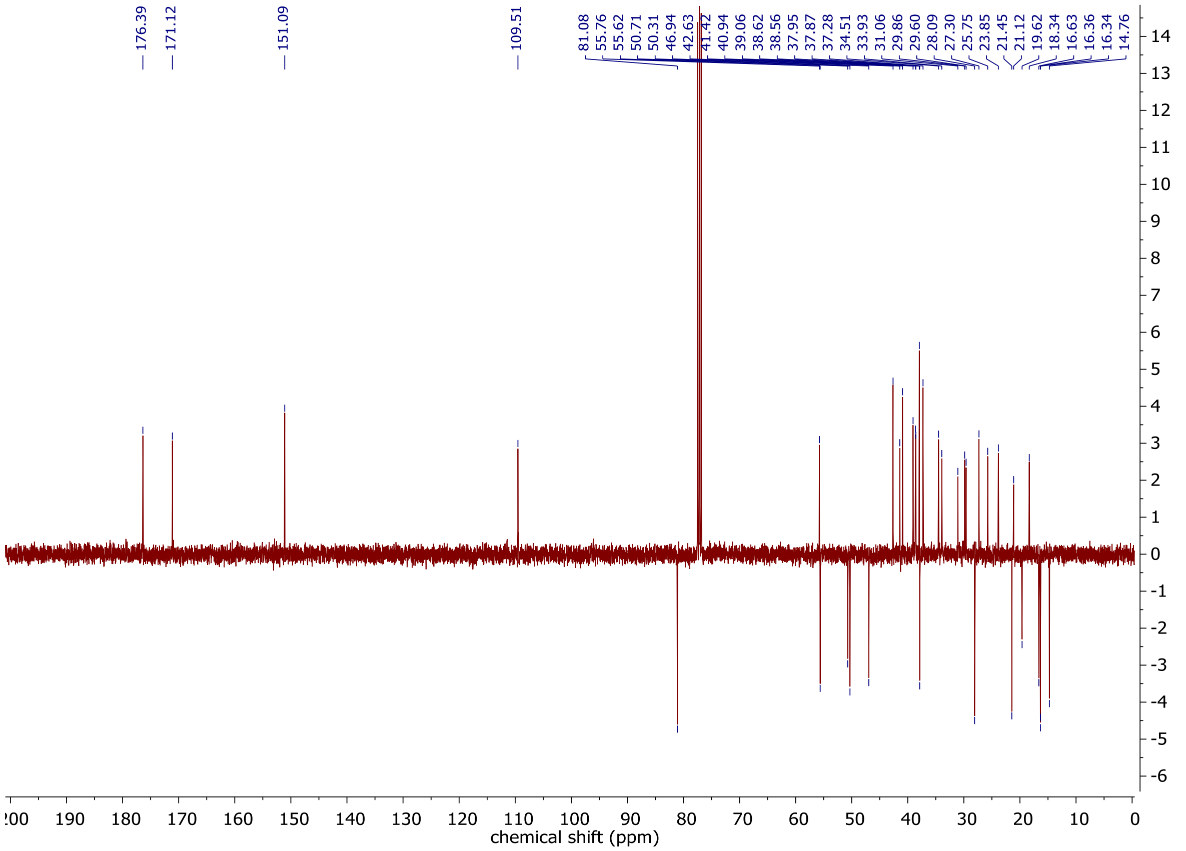

NMR spectra of 23

^{1}H NMR

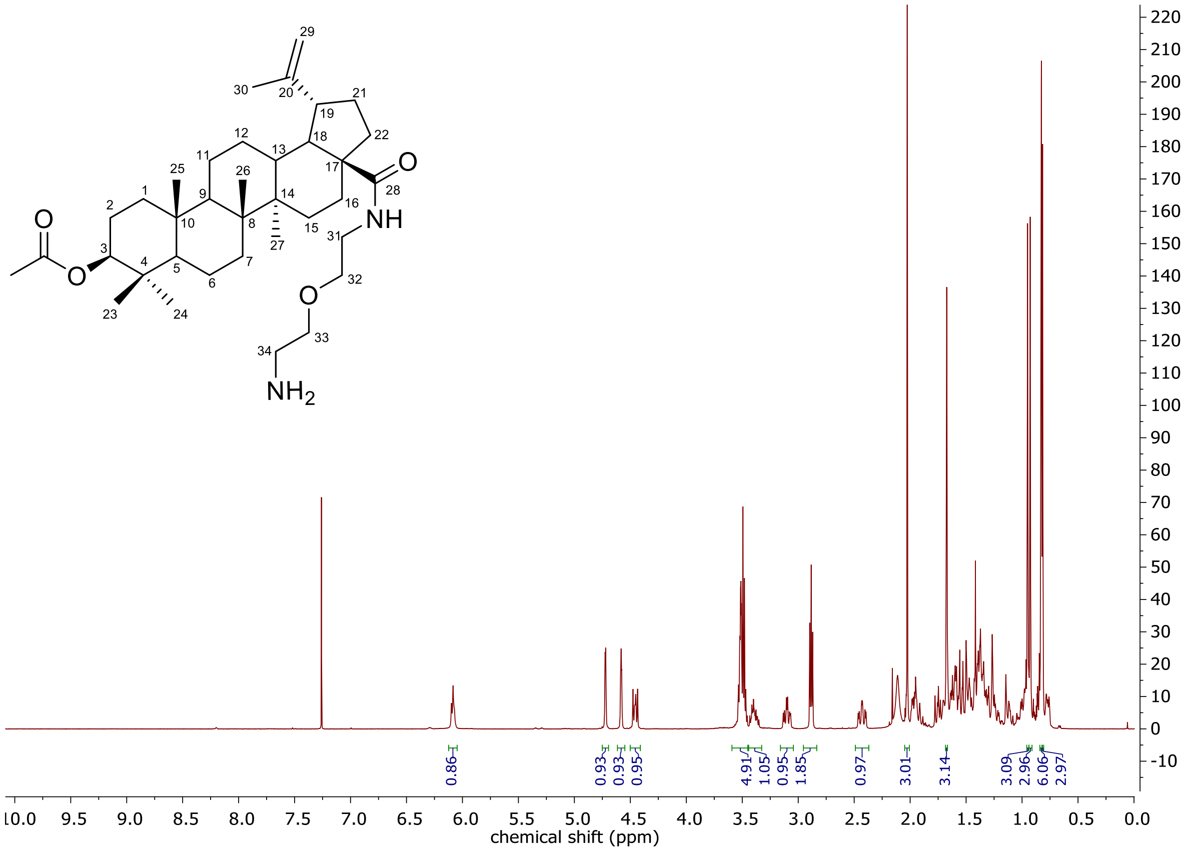

^{13}C NMR (APT)

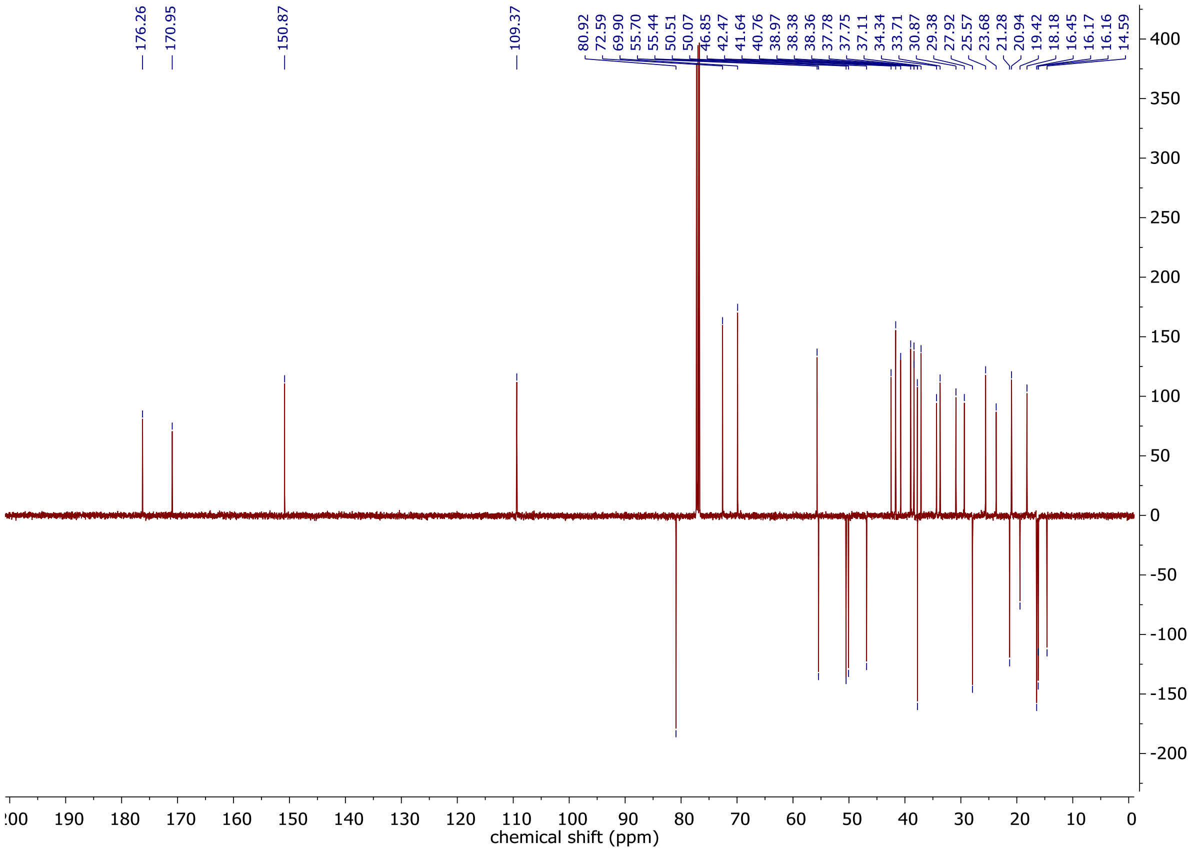

NMR spectra of 24

^{1}H NMR

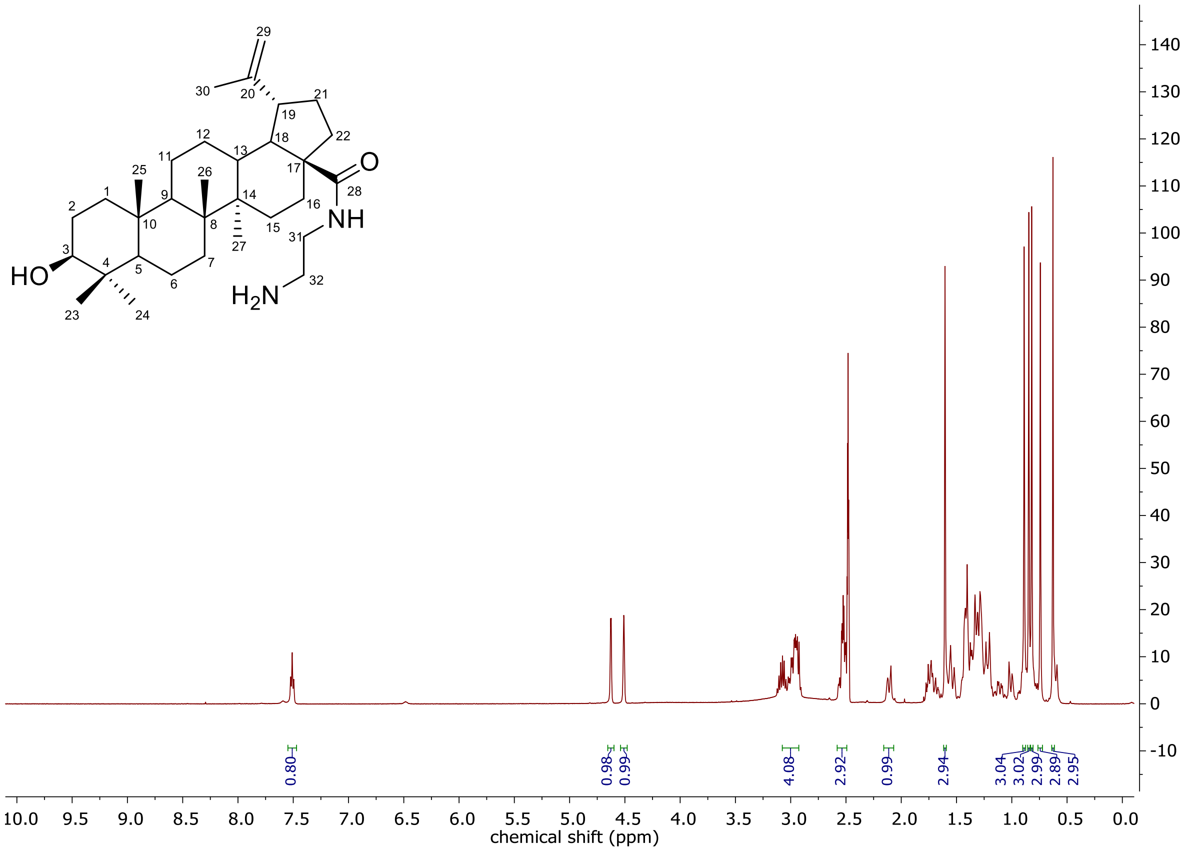

^{13}C NMR (APT)

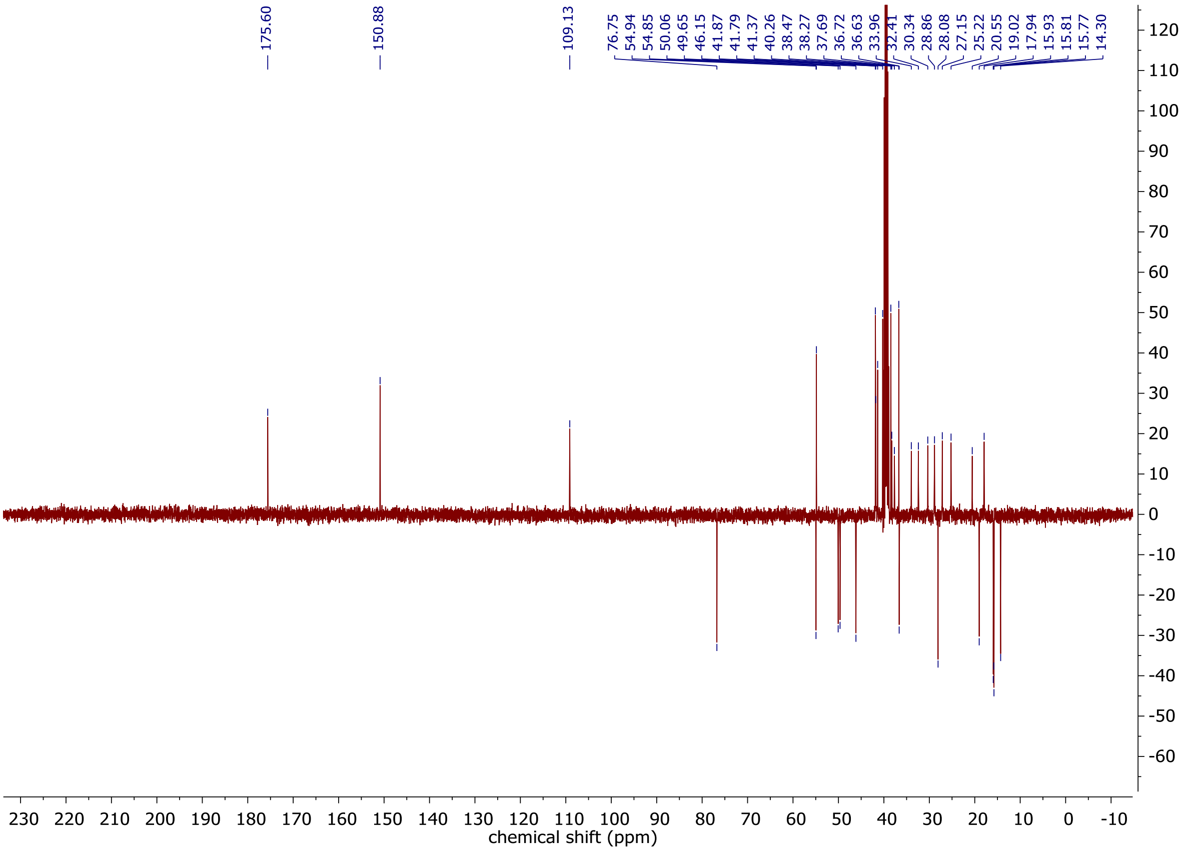

NMR spectra of 25

^{1}H NMR

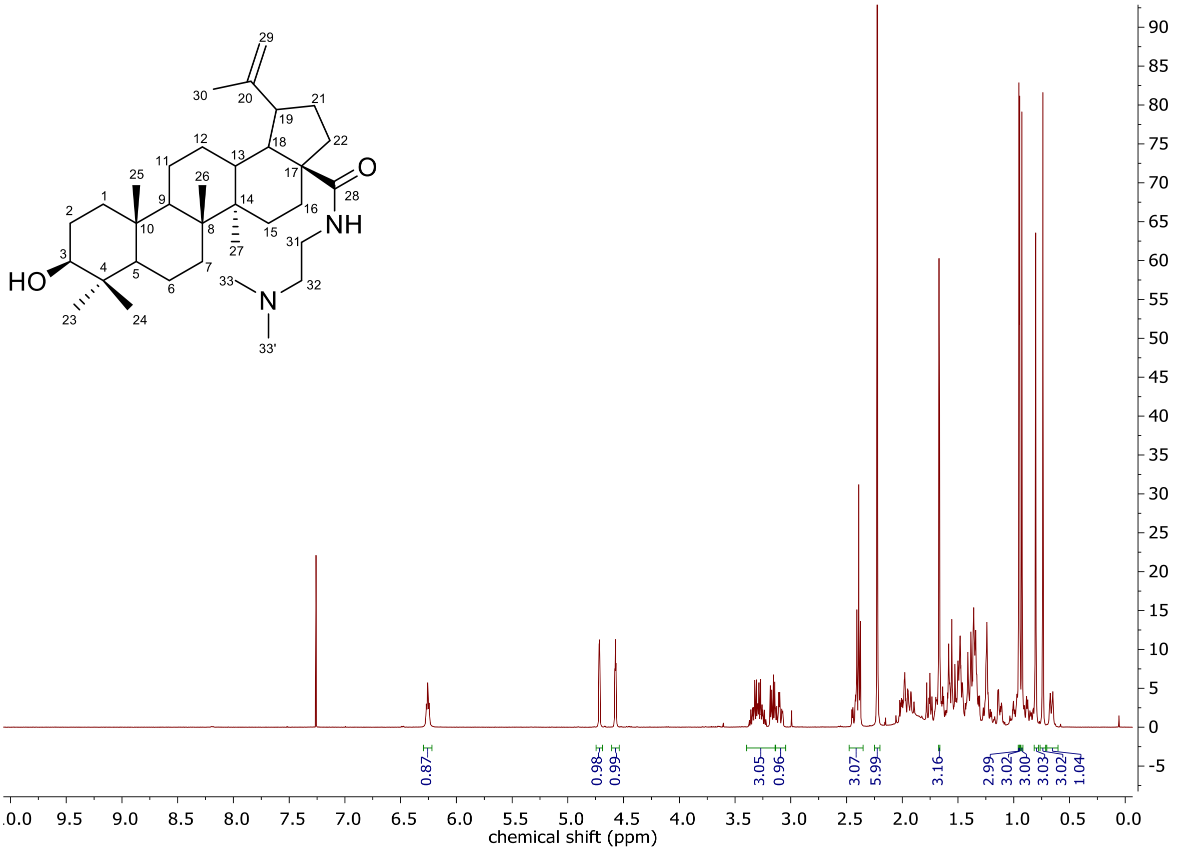

^{13}C NMR (APT)

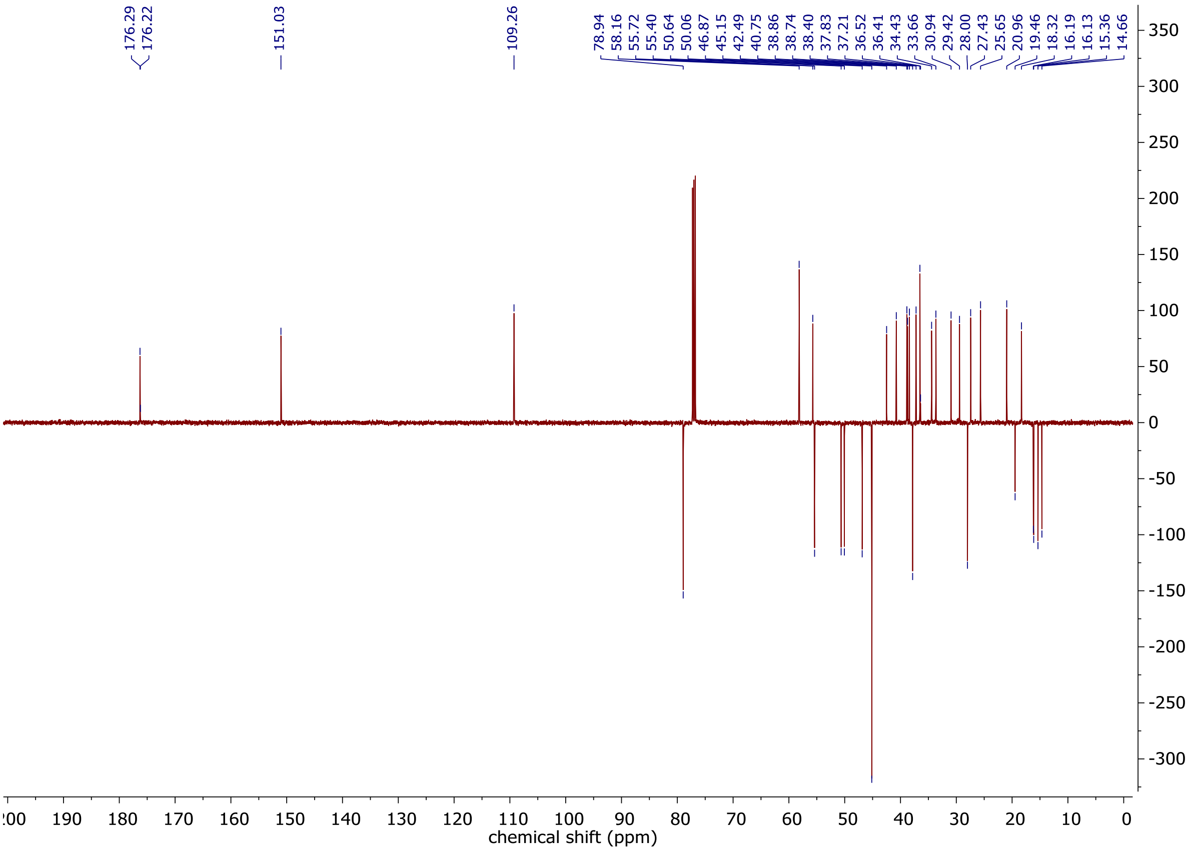

NMR spectra of 26

^{1}H NMR

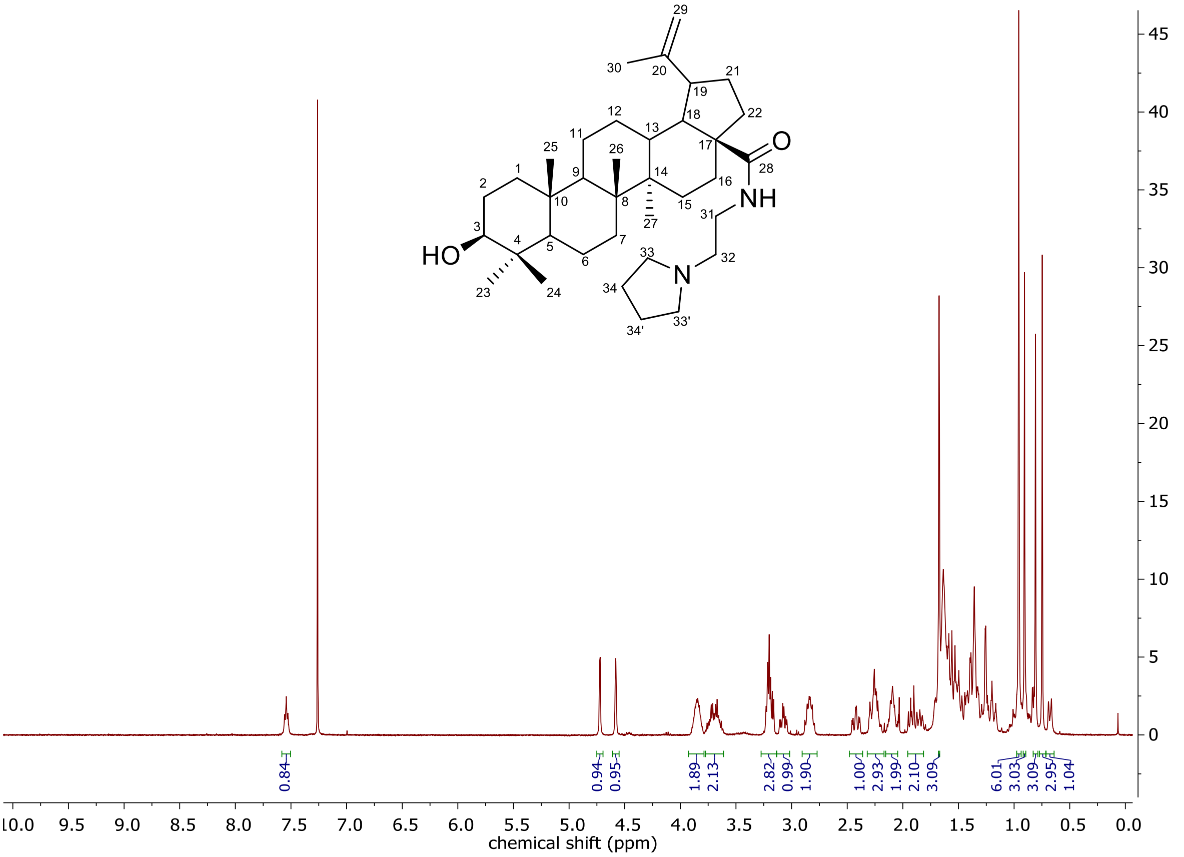

^{13}C NMR (APT)

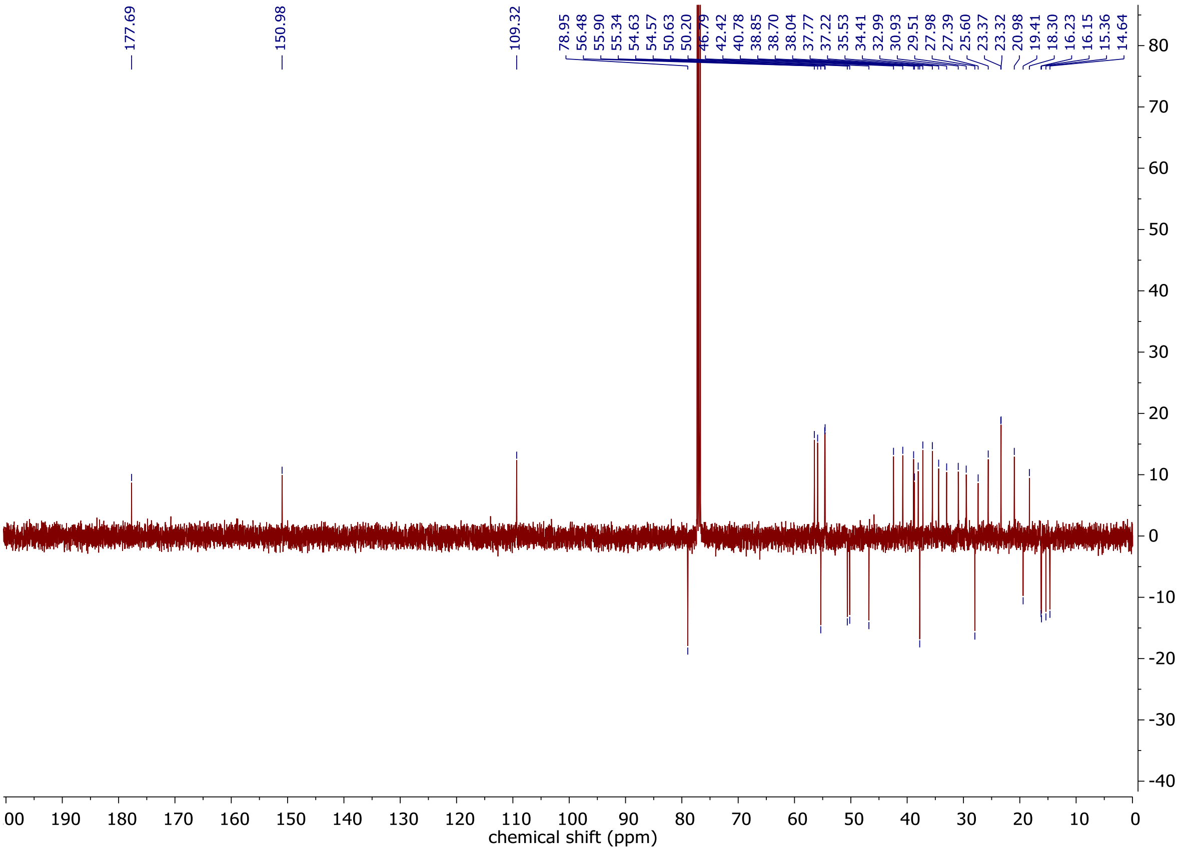

NMR spectra of 27

^{1}H NMR

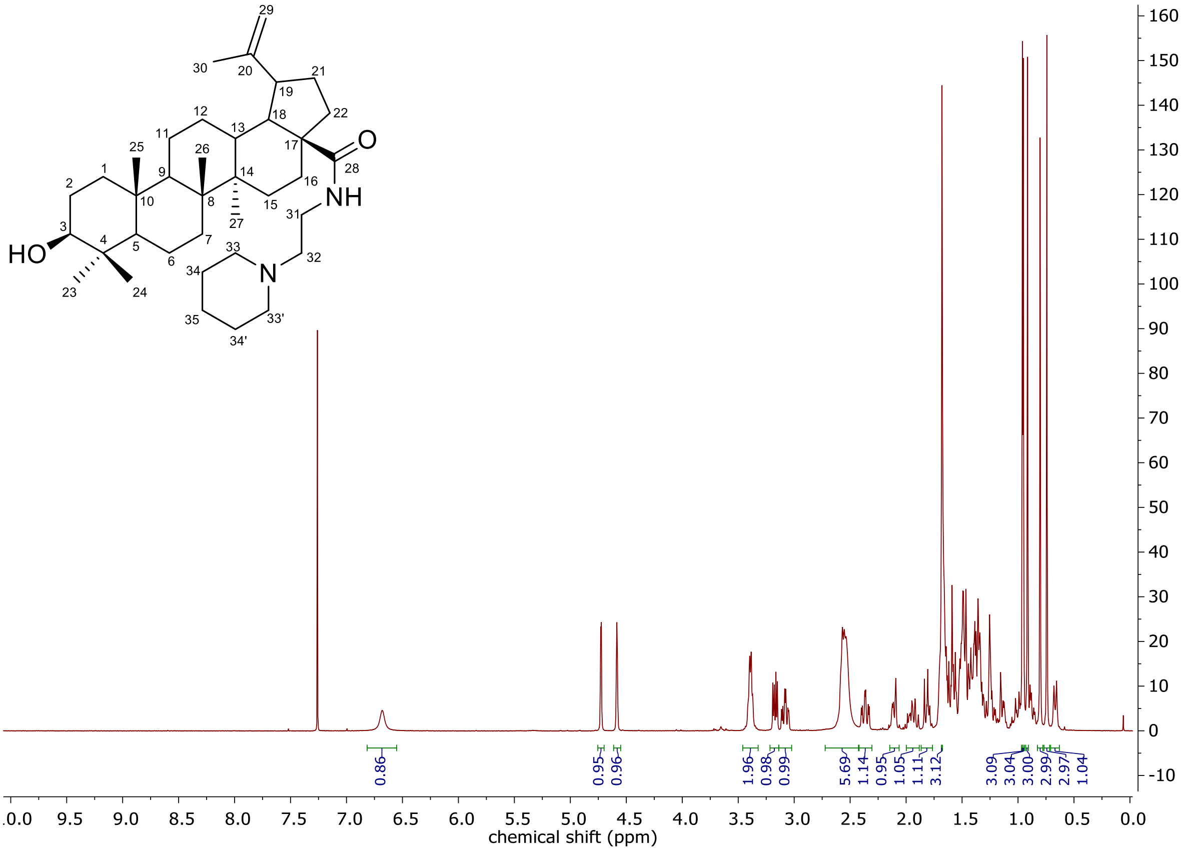

^{13}C NMR (APT)

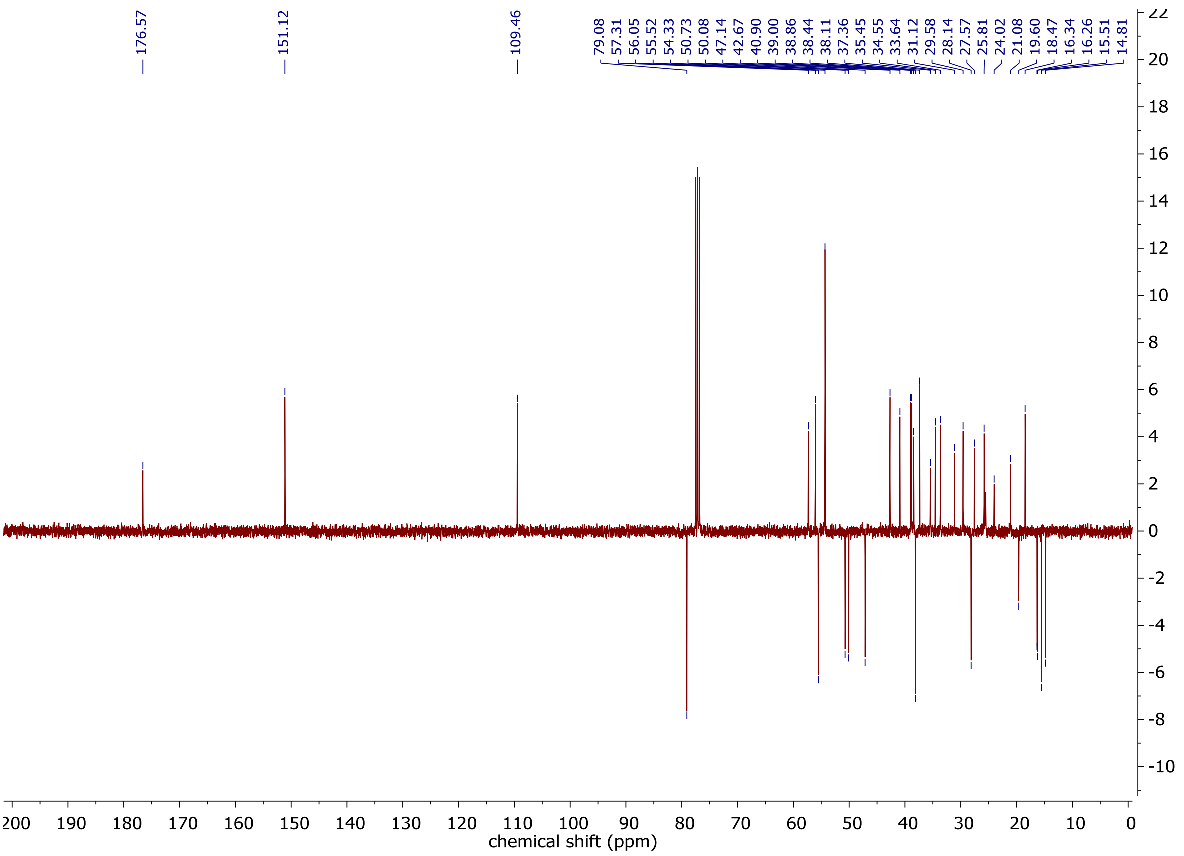

NMR spectra of 28

^{1}H NMR

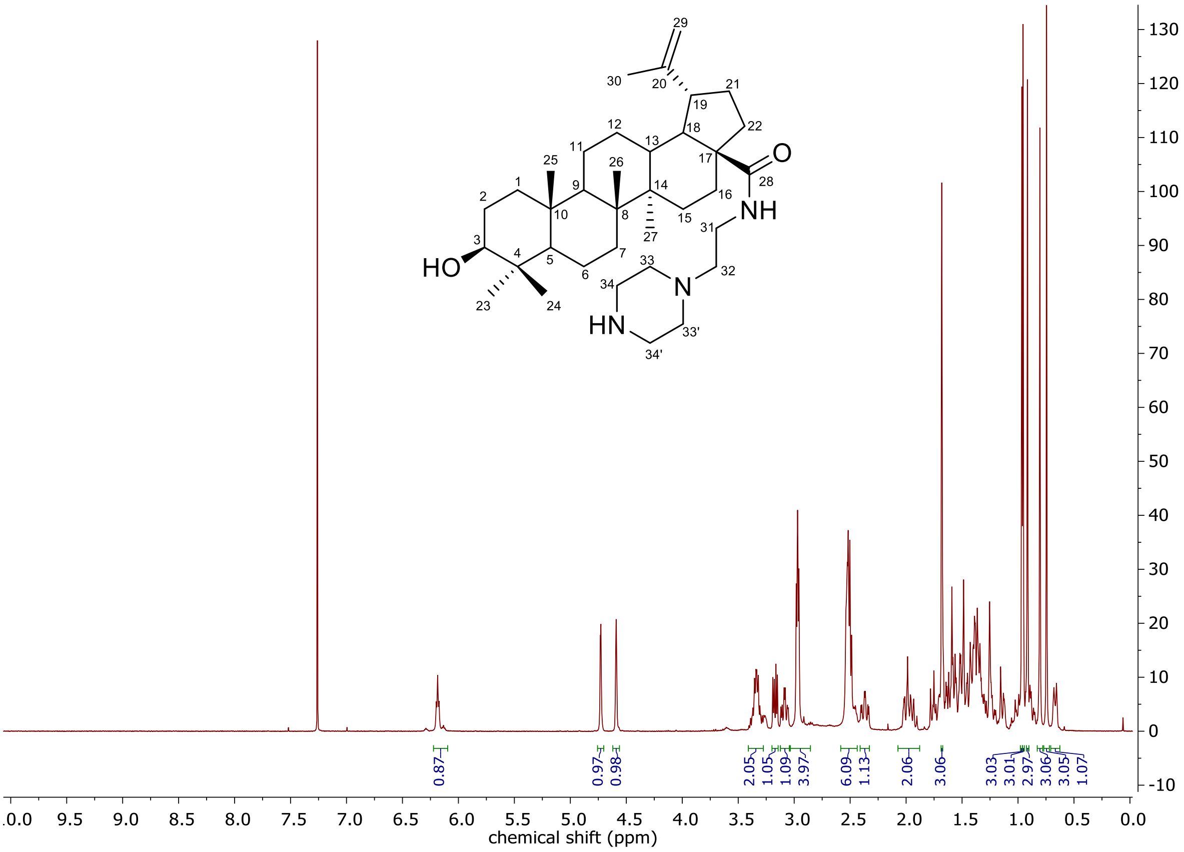

^{13}C NMR (APT)

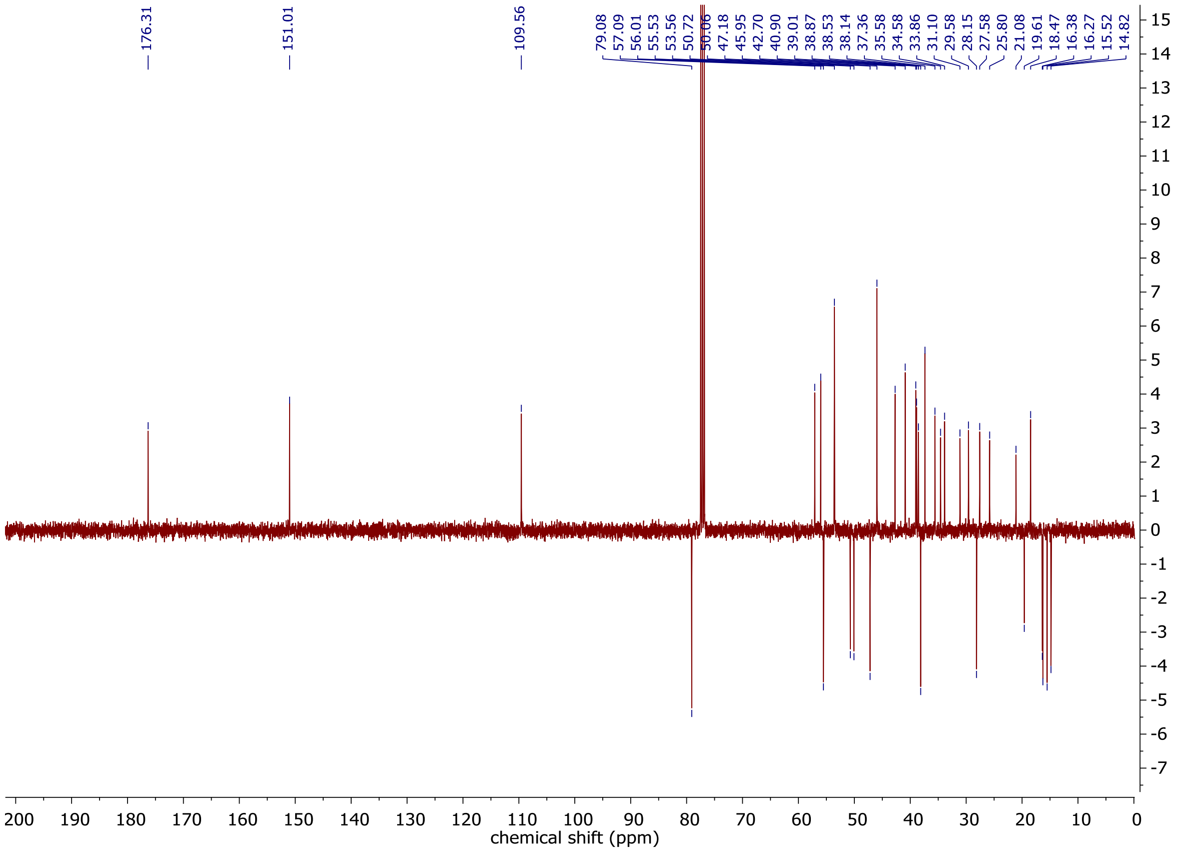

NMR spectra of 29

^{1}H NMR

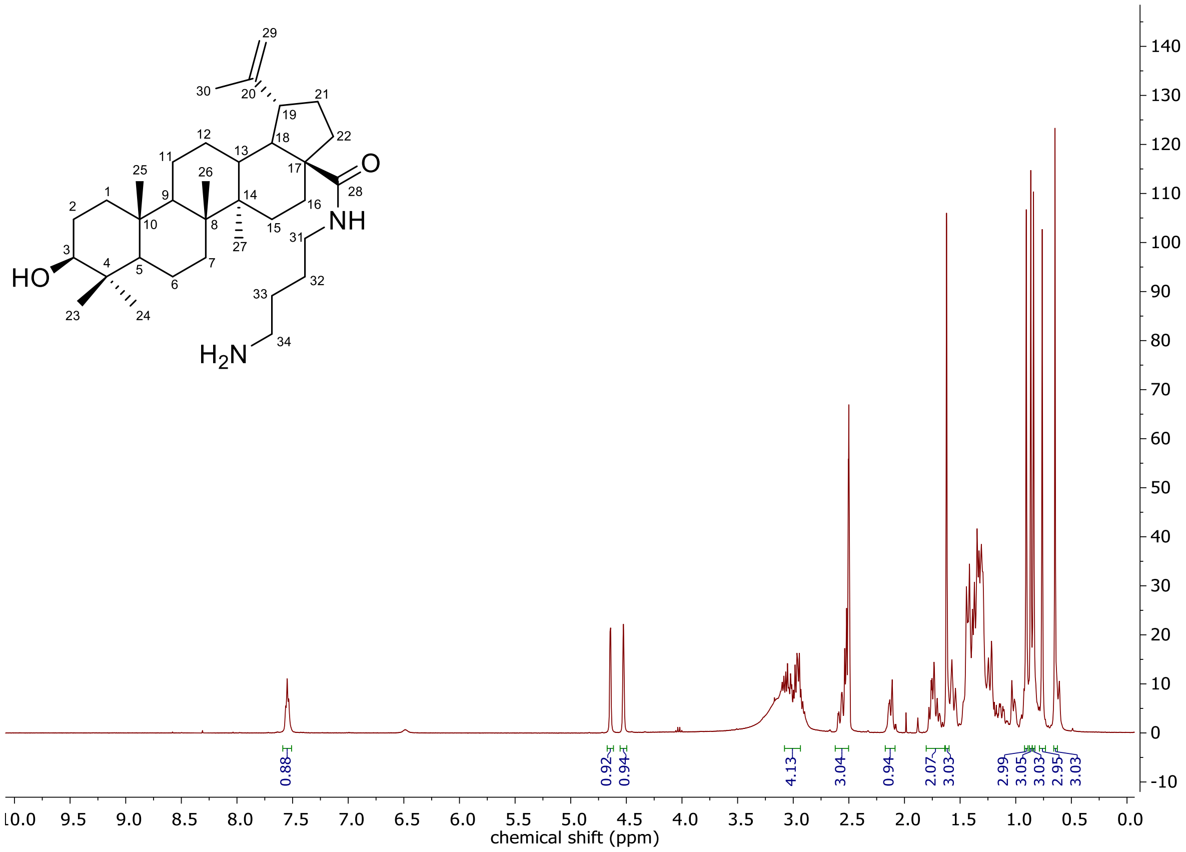

^{13}C NMR (APT)

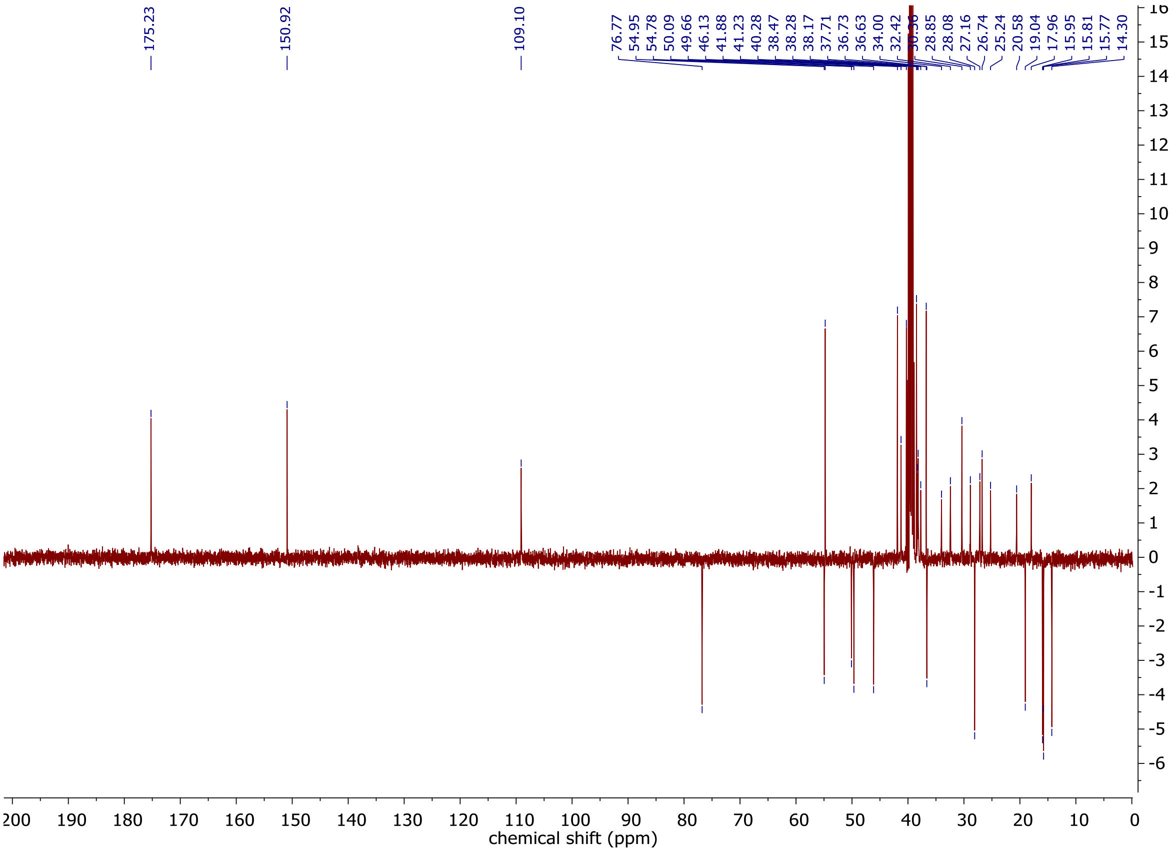

NMR spectra of 30

^{1}H NMR

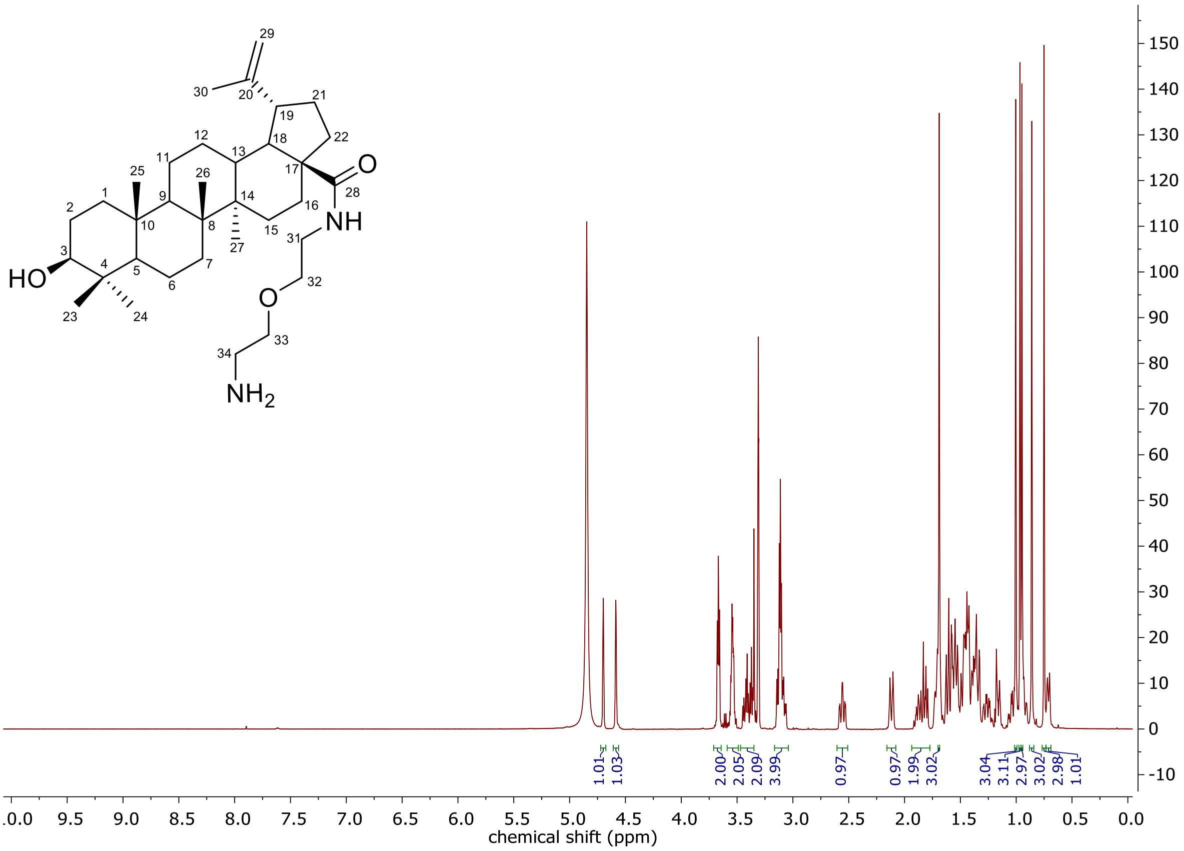

^{13}C NMR (APT)

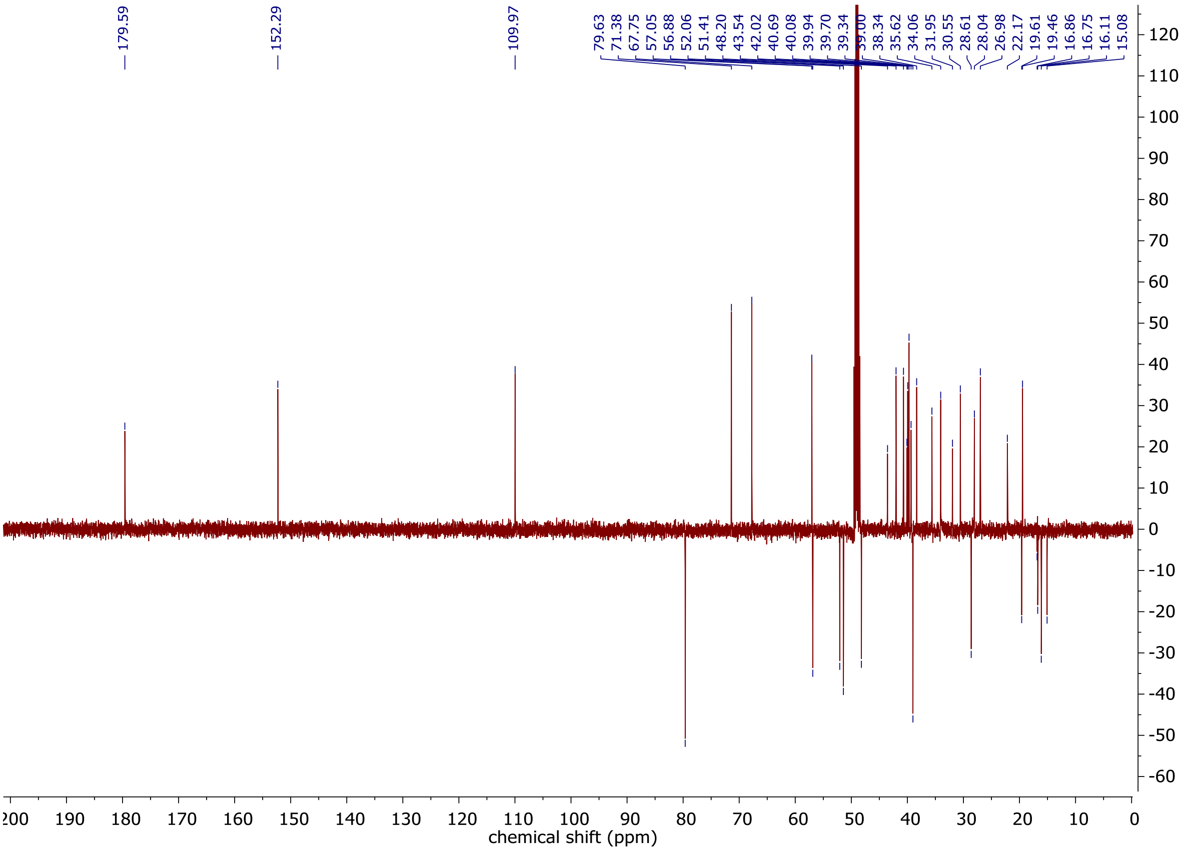
179.59
152.29
109.97
79.63
71.38
67.75
57.05
56.88
52.06
51.41
48.20
43.54
42.02
40.69
40.08
39.94
39.70
39.34
39.00
38.34
35.62
34.06
31.95
30.55
28.61
28.04
26.98
22.17
19.61
19.46
16.86
16.75
16.11
15.08
chemical shift (ppm)
